# Genotyping-by-sequencing provides the discriminating power to investigate the subspecies of *Daucus carota* (Apiaceae)

**DOI:** 10.1186/s12862-016-0806-x

**Published:** 2016-10-28

**Authors:** Carlos I. Arbizu, Shelby L. Ellison, Douglas Senalik, Philipp W. Simon, David M. Spooner

**Affiliations:** 1 Department of Horticulture, University of Wisconsin-Madison, 1575 Linden Drive, Madison, WI 53706-1590 USA; 2 USDA-Agricultural Research Service, Vegetable Crops Research Unit, University of Wisconsin-Madison, 1575 Linden Drive, Madison, WI 53706-1590 USA

**Keywords:** Carrot, *Daucus carota*, Genotyping-by-sequencing (GBS), Phylogeny, Single nucleotide polymorphisms (SNPs)

## Abstract

**Background:**

The majority of the subspecies of *Daucus carota* have not yet been discriminated clearly by various molecular or morphological methods and hence their phylogeny and classification remains unresolved. Recent studies using 94 nuclear orthologs and morphological characters, and studies employing other molecular approaches were unable to distinguish clearly many of the subspecies. Fertile intercrosses among traditionally recognized subspecies are well documented. We here explore the utility of single nucleotide polymorphisms (SNPs) generated by genotyping-by-sequencing (GBS) to serve as an effective molecular method to discriminate the subspecies of the *D. carota* complex.

**Results:**

We used GBS to obtain SNPs covering all nine *Daucus carota* chromosomes from 162 accessions of *Daucus* and two related genera. To study *Daucus* phylogeny, we scored a total of 10,814 or 38,920 SNPs with a maximum of 10 or 30% missing data, respectively. To investigate the subspecies of *D. carota*, we employed two data sets including 150 accessions: (i) rate of missing data 10% with a total of 18,565 SNPs, and (ii) rate of missing data 30%, totaling 43,713 SNPs. Consistent with prior results, the topology of both data sets separated species with 2*n* = 18 chromosome from all other species. Our results place all cultivated carrots (*D. carota* subsp. *sativus*) in a single clade. The wild members of *D. carota* from central Asia were on a clade with eastern members of subsp. *sativus*. The other subspecies of *D. carota* were in four clades associated with geographic groups: (1) the Balkan Peninsula and the Middle East, (2) North America and Europe, (3) North Africa exclusive of Morocco, and (4) the Iberian Peninsula and Morocco. *Daucus carota* subsp. *maximus* was discriminated, but neither it, nor subsp. *gummifer* (defined in a broad sense) are monophyletic.

**Conclusions:**

Our study suggests that (1) the morphotypes identified as *D. carota* subspecies *gummifer* (as currently broadly circumscribed), all confined to areas near the Atlantic Ocean and the western Mediterranean Sea, have separate origins from sympatric members of other subspecies of *D. carota*, (2) *D. carota* subsp. *maximus*, on two clades with some accessions of subsp. *carota*, can be distinguished from each other but only with poor morphological support, (3) *D. carota* subsp. *capillifolius*, well distinguished morphologically, is an apospecies relative to North African populations of *D. carota* subsp. *carota*, (4) the eastern cultivated carrots have origins closer to wild carrots from central Asia than to western cultivated carrots, and (5) large SNP data sets are suitable for species-level phylogenetic studies in *Daucus*.

**Electronic supplementary material:**

The online version of this article (doi:10.1186/s12862-016-0806-x) contains supplementary material, which is available to authorized users.

## Background

The center of diversity of the genus *Daucus* is in the Mediterranean region [1]. *Daucus* species also occur elsewhere, with one species (*D. glochidiatus* [Labill.] Fischer & al.) in Australia, four species in the American continent (*D. carota* L., *D. montanus* Humb. & Bonpl. ex Schütt., *D. montevidensis* Link ex Sprengel, *D. pusillus* Michx.); *D. carota* occurs in many continents worldwide. Wild and cultivated carrots (*D. carota* L. sensu lato) belong to the *D. carota* complex. Its constituent taxa all possess 2*n* = 18 chromosomes, have weak biological barriers to interbreeding, and some of these taxa are difficult to define taxonomically [2, 3], making the *D. carota* complex the most problematic species group in the Apiaceae family [4].

The classification of the members of the *D. carota* complex has attracted the interest of various researchers [3]. Germplasm curators have relied on local floras for identifying *Daucus* such as those from Algeria [5], the Azores [6], Europe [7], the Iberian Peninsula and Balearic Islands [8], Libya [9], Morocco [10], Palestine [11], Portugal [12], Syria [13], Tunisia [14, 15], and Turkey and the East Aegean Islands [16]. Currently, there is no consensus about the number of subspecies of *D. carota*. More than 60 species have been proposed for the phenotypic variants observed within the *D. carota* complex [3]. For instance, 11 wild subspecies were recognized by Heywood [2, 17], five by Arenas and García-Martin [18], and five in the latest comprehensive morphoanatomical classification of *Daucus* by Sáenz [1] (subsp. *carota*, subsp. *gummifer*, subsp. *hispanicus*, subsp. *maritimus*, and subsp. *maximus*). Pujadas Salvà [19] proposed nine subspecies for the Iberian Peninsula plus Balearic Islands (subsp. *carota,* subsp. *cantabricus*, subsp. *commutatus*, subsp. *gummifer,* subsp. *halophilus*, subsp. *hispanicus,* subsp. *majoricus,* subsp. *maximus,* and subsp. *sativus*).

Several molecular approaches have been used to examine the diversity and genetic relationships of *D. carota*. St. Pierre et al. [20] used isozymes to study 168 accessions of the *D. carota* complex from 32 countries and could not separate them into distinct groups. Random amplified polymorphic DNA (RAPD) and amplified fragment length polymorphisms (AFLP) were employed by Nakajima et al. [21] and showed that all accessions of *D. carota* group into a major clade. Vivek and Simon [22, 23] used restriction fragment length polymorphisms (RFLPs) of nuclear, plastid, and mitochondrial DNA and interpreted their results to be generally concordant with the classification proposed by Sáenz [1]. However, only one additional subspecies was studied (subsp. *drepanensis*). Using AFLPs, Shim and Jørgensen [24] showed wild and cultivated carrot clustered separately. Similar results were obtained by Bradeen et al. [25] with AFLPs and inter-simple sequence repeats (ISSR) and they concluded wild carrots had no substructure. Rong et al. [26] obtained a *Daucus* phylogeny using SNPs and found the subspecies of *D. carota* to be intermixed. Later, Lee and Park [27] mentioned *D. sahariensis*, *D. syrticus* and *D. gracilis* are probably the closest relatives to *D. carota*. In an attempt to characterize the populations of *D. carota* present in São Miguel Island (Azores, Portugal), Matias Vaz [28] used one nuclear ortholog, nuclear ribosomal DNA ITS, and morphological descriptors, and concluded that the classification of *D. carota* remained problematic. Other morphological studies [3, 29–32] did not distinguish the subspecies of *D. carota*. However, Iorizzo et al. [33] developed 3,326 single nucleotide polymorphisms (SNPs) to study the genetic structure and domestication of carrot. Using seven wild subspecies of *D. carota* (other than subsp. *carota*) as outgroups, they found a clear separation between wild (subsp. *carota*) and cultivated (subsp. *sativus*) accessions of *Daucus*.

Other studies distinguished *Daucus* species outside of the complex. Using an integrated approach consisting of morphology, together with the ribosomal internal transcribed spacers (ITS), the plastid *trnQ-rps16* intergenic spacer and plastid *rps16* intron sequences, the existence of a neglected species from North Africa, *D. mauritii* (Sennen ex Maire) Sennen, was confirmed [34]. More recently, Spooner et al. [35] demonstrated the utility of eight nuclear orthologs to infer the phylogeny of *Daucus*. This study revealed all the subspecies of *D. carota*, *D. capillifolius* (that they named as *D. carota* subsp. *capillifolius* (Gilli) Arbizu) and *D. syrticus* are closely related, but was ineffective at separating the subspecies. Arbizu et al. [36] used 94 nuclear orthologs obtained by next-generation sequencing technology to examine multiple accessions per species of *Daucus*, and could distinguish the species well, including the 2n = 18 species *D. syrticus*, but not the subspecies of *D. carota*. Similarly, a recent investigation [37] used ITS sequences and three plastid DNA sequences to study the phylogeny of subtribe Daucinae and showed accessions of the *D. carota* complex did not group the subspecies together.

The last decade has seen tremendous advances in genome-scale data collection and analysis, allowing researchers from various disciplines to address new questions. A major innovation for the plant systematics community is high-throughput DNA sequencing [38] to infer phylogenetic relationships among recently diverged species or populations [39, 40]. Challenges for taxonomic resolution at low taxonomic levels mainly arise due to biological events such as gene flow by hybridization and introgression [41], gene duplication [42], horizontal gene transfer [43], and incomplete lineage sorting [44]. To address the challenge of resolving the phylogenetic relationships among very closely related species, large genome-scale data sets can be used [45]. Reduced-representation methods provide powerful and cost-effective tools, producing abundant large-scale genomic data [46] and have been used in many phylogenetic studies [45, 47–51].

Genotyping-by-sequencing (GBS) is one such genome-wide reduced representation method that generates sequence variants by utilizing next-generation sequencing technology, producing a powerful and cost-effective genotyping procedure [52]. A large number of variants are generated by GBS [53] and have been applied to a wide range of crops such as barley [54], lentils [51], maize [55], potato [56], reed canarygrass [57], rice [58], soybean [59], switchgrass [60], and wheat [61]. GBS has been used to infer the classification of wild species like chickpea [62], lentils [51], sedges [45], and tomatoes [63].

*Daucus* would benefit from a modern taxonomic monograph, but currently there is no consensus about the number of species in the genus or the number of subspecies within the *D. carota* complex. We here explore the use of GBS to discover SNPs distributed along all nine chromosomes, and evaluate their phylogenetic utility within *Daucus*, through the examination of 164 accessions, and analyses using concatenated data and a coalescent model. We then integrate our GBS results with prior morphological data for a coordinated approach to distinguish subspecies within the *D. carota* complex.

## Methods

### Plant materials

We examined 162 accessions of *Daucus*, and two accessions of related outgroup genera, *Orlaya* and *Rouya* (164 accessions in total) (Additional file 1: Table S1). A previous study [36] confirmed that the phylogeny of *Daucus* is divided into two main clades, A (including A′) and B. Sub-clade A′ contains members of the *Daucus carota* complex. In the present study, the *Daucus carota* complex is represented by 144 accessions. All accessions were obtained from the United States National Plant Germplasm System, maintained at the North Central Regional Plant Introduction Station (NCRPIS) in Ames, Iowa. As indicated above, subspecies boundaries in *D. carota* are controversial and no two authors agree on how many there are. This issue was investigated by Arbizu et al. [29] and Spooner et al. [31] who recognized three wild subspecies (subsp. *capillifolius*, subsp. *carota*, subsp. *gummifer*) and one cultivated subspecies (subsp. *sativus*). We chose all accessions available as germplasm (collected in 35 countries), and used these names. Based on the present results (below, partly supporting *D. carota* subsp. *maximus*, although on a clade with some accessions of subsp. *carota*), we reevaluated the characters differentiating subsp. *carota* and subsp. *maximus* and reanalyzed the morphological data of Spooner et al. [31]. Further details of the accessions examined in this study are available at the Germplasm Resources Information Network - GRIN (https://npgsweb.ars-grin.gov/gringlobal/search.aspx).

### Genotyping-by-sequencing data set

All 164 accessions were planted in a greenhouse at the University of Wisconsin-Madison and genomic DNA was extracted using the CTAB method [64] from young leaves. Genomic DNA quality was initially assessed by visualizing 900 ng of each sample on a 1% agarose gel and twenty random samples were digested with *Eco*RI following the manufacturer′s protocol (Promega, Madison, WI). In addition, DNA quality was verified by measuring the optical density at a wavelength of 260 and 280 nm with a ND-1000 Nanodrop Spectrophotometer (NanoDrop Technologies, Wilmington, DE). Samples were sent to the UW-Madison Biotechnology Center for DNA sequencing. First, quantity of the genomic DNA was evaluated with Quant-iT™ PicoGreen® dsDNA kit (Life Technologies, Grand Island, NY). Then, the protocol described by Elshire et al. [52] was followed. In short, samples of genomic DNA were digested using a methylation-sensitive restriction enzyme, *Ape*KI (New England Biolabs, Ipswich, MA) with recognition site GCWGC, where W is A or T. Barcoded adapters amenable to Illumina sequencing were then added by ligation with T4 ligase (New England Biolabs, Ipswich, MA). Adapter-ligated samples were pooled and amplified to provide library quantities amenable for sequencing, and adapter dimers were removed by SPRI bead purification. Quality and quantity of the finished libraries were assessed using the Agilent Bioanalyzer High Sensitivity Chip (Agilent Technologies, Inc., Santa Clara, CA) and Qubit® dsDNA HS Assay Kit (Life Technologies, Grand Island, NY), respectively. Libraries were standardized to 2 μM. Cluster generation was performed using HiSeq SR Cluster Kit v3 cBot kits (Illumina Inc, San Diego, CA, USA). Finally, 100 bp single-end reads were sequenced using the HiSeq SBS Kit v3 (50 Cycle) (Illumina Inc.) on a HiSeq2000 sequencer. Images were analyzed using the standard Illumina Pipeline, version 1.8.2. A second set of libraries was sequenced using the same protocol described above. Here we included samples of the first libraries that produced a low number of reads, and additional accessions to increase the representation of the *D. carota* complex. To quantify the number of reads per library, we processed the raw GBS data (FASTQ format) using the program process_radtags.pl, which is part of the STACKS pipeline [65].

Data were then analyzed using the bioinformatics pipeline TASSEL-GBS version 4.3.13 [66, 67]. Briefly, GBS sequence tags were identified in all FASTQ files by indicating the restriction enzyme (*Ape*KI) and the DNA barcodes for each sample used for library preparation. Only reads having an intact barcode sequence were kept. Then, unique tags from each sequence were merged producing a master tag count file in FASTQ text format (argument –*t* was used), which was used as an input to the program Burrows-Wheeler Aligner (BWA) version 0.7.12 [68], to align all the tags against the carrot reference genome LNRQ01000000.1 [69]. The alignment file in SAM format was converted into a binary tagsOnPhysicalMap (.topm) file. We then used the SeqToTBTHDF5 plug-in to obtain a TagsByTaxa (TBT) file in HDF5 format containing the number of times each GBS tag was observed in each sample. The TBT file was transposed into a tag-optimized orientation, and together with the .topm file, they were used as input for the SNP calling plug-in. For each carrot chromosome, one HapMap genotype file was generated (nine in total), followed by the search of duplicate SNPs and their corresponding merging in each HapMap file (options -*misMat* = 0.1 and -*callHets* were used). GBSHapMapFilters plug-in was then used to discard samples with a SNP call rate and minimum R-square value for LD filter less than 0.1 (arguments -*mnTCov* = 0.1 and -*mnR2* = 0.1 were called). Further data curation was conducted using VCFtools version 0.1.14 [70] with the following criteria of retention: (i) minimum minor allele frequency of 0.1, (ii) maximum minor allele frequency of one, (iii) number of alleles less than or equal to two, and (iv) maximum missing data of 0.1 or 0.3. To impute missing genotypes, we used the program Beagle version 4.0 [71] with arguments *burnin-its* = 10, *phase-its* = 10, *impute-its* = 10. Beagle software uses a Hidden Markov model for inferring haplotype phase of the samples and then filling in missing genotypes.

### Phylogenetic analyses

All SNPs were concatenated into a single alignment. Maximum likelihood and Bayesian analyses were conducted via the CIPRES [72] portal at the San Diego Supercomputer Center (http://www.phylo.org) with the GTR + G nucleotide substitution model using RAxML version 8.2.4 [73] and MrBayes version 3.2.6 [74], respectively. Selection of the best-fit evolutionary model for our GBS data set was attempted using jModelTest version 2.1.4 [75] on CIPRES. However, this gave an error message indicating *Phyml* command line cannot run. *Phyml* might be crashing due to the elevated number of samples (164 and 150). The most common model of evolution for DNA analysis is general time-reversible (GTR) [76] and that is the main reason only GTR-based models are implemented in RAxML [77]. In addition, the RAxML manual [77] strongly suggests avoiding *Pinv* + *Gamma* model. Hence, we continued our phylogenetic analyses with GTR + G. We obtained the best-scoring ML tree from 100 independent ML tree searches, and then 1,000 nonparametric bootstrap inferences were performed with the same program. All ML analyses were performed using four data sets: (1) 164 accessions with 10% missing imputed genotypes, (2) 164 accessions with 30% missing imputed genotypes, (3) 150 accessions with 10% missing imputed genotypes, and (4) 150 accessions with 30% missing imputed genotypes. The data set with 164 accessions represents five sub-species of *Daucus carota*, eight *Daucus* species and two related genera (*Orlaya* and *Rouya*). On the other hand, the 150 accessions are comprised of five sub-species of *Daucus carota* and one species, *D. syrticus*, as outgroup. We first used all 164 samples to reconstruct the phylogeny of *Daucus* to confirm the sister group of the *D. carota* complex. To do so, we rooted our tree on *Orlaya daucoides* based on Arbizu et al. [36]. Because of this wider analysis, subsequent ML analyses of the *D. carota* complex were executed using *D. syrticus* as outgroup (150 accessions in total).

Bayesian analysis was carried out using only the data set of the *D. carota* complex (150 accessions) containing 10% missing imputed genotypes. We conducted two independent four-chain 50 million generation runs per input file, sampled every 1,000 generations. Tracer v1.6 (http://tree.bio.ed.ac.uk/software/tracer/) was used to analyze the convergence to the stationary distribution and the effective sample size (ESS) of each parameter of each input file. We discarded the first 25% of generations as burn-in.

A lineage tree was constructed considering only members of the *D. carota* complex, including *D. syrticus*, using the two data sets containing imputed genotypes of maximum missing rate of 0.1 or 0.3. The method we used was implemented in the software SVDquartets [78], which assumes each SNP has its own genealogical history and relationships among quartets of taxa are inferred under the coalescent model. In addition, we inferred a species level phylogeny by renaming the 150 accessions employed for the lineage tree construction with new proposed subspecies names determined in this study (Additional file 1: Table S1). The name for the plants initially identified as putative hybrids of subsp. *carota* and subsp. *capillifolius* is subsp. *carota*, as proposed by Spooner et al. [31]. To conduct these analyses, we evaluated all possible quartets including 100 nonparametric bootstrap replicates. SVDquartets was executed in the program PAUP* version 4.0a147 [79] and the resulting trees were viewed in FigTree version 1.4.0 (http://tree.bio.ed.ac.uk/software/figtree/).

### Population structure

The composition of population structure in the 150 accessions belonging to the *D. carota* complex, including *D. syrticus*, was determined using all original filtered SNPs in the two data sets comprising 10 or 30% missing imputed data. First, our VCF data sets were converted into PLINK PED [80] format using the *--plink* option in software VCFtools. Then, the PGDSpider program [81] was used to convert PLINK PED format into STRUCTURE format. We employed the Bayesian clustering program STRUCTURE version 2.3.4 [82] with populations (K value) ranging from 1 to 14, replicated ten times, with a burn−in length of 20,000 and 50,000 Monte Carlo iterations. An admixture model with no previous population information was considered; all other parameters were set to default values. STRUCTURE results were then processed in the software STRUCTURE HARVESTER [83], detecting the most likely number of clusters by using the rate of change in the log probability of our data between successive values of K, also known as Delta K [84]. To minimize variance across all the ten iterations of the selected K values, we used the program CLUMPP version 1.1.2 [85] utilizing the *LargeKGreedy* algorithm with 1,000 permutations. Population structure was then visualized using DISTRUCT software version 1.1 [86].

### Morphological analysis

We analyzed the data set of morphological characters previously obtained by Spooner et al. [31] for the *D. carota* complex. Accessions identified as *D. carota* subsp. *sativus* (cultivated carrot) were not included in the present analysis; only wild *Daucus carota* accessions. Samples of wild carrots were classified into five groups (*D. carota* subsp. *capillifolius*, subsp. *carota*, subsp. *gummifer*, subsp. *maximus*, and hybrids between subsp. *carota* with subsp. *capillifolius*) based on our molecular phylogenetic analyses, population structure results, and the previous study conducted by Spooner et al. [31], with most accessions examined in common among studies.

A data set consisting of 127 accessions of the *D. carota* complex (minus subsp. *sativus*) and 23 continuous characters (stem, leaf, flower and mericarp) was examined with a stepwise discriminant analysis (linear, common variance) in JMP® version 11.2 (SAS Institute Inc.) using a backward selection method. A model with significant variables in identifying accession structure was obtained by removing characters one at a time until the model F-test *p* value ≤ 0.05. We then performed a canonical variate analysis (CVA). A descriptive statistical analysis was conducted to verify the mean, median, standard deviation and range of values. Utilizing R software version 3.2.0 [87], box plots were constructed as a graphic tool to visualize comparisons across accessions of wild carrots.

## Results

The GBS analysis pipeline implemented in TASSEL-GBS version 4.3.13 [66, 67] identified a total of 16,291,308 and 14,548,150 unique 100-bp sequence tags for the following two data sets, respectively: (1) 162 accessions of *Daucus* and two accessions of related genera (*Orlaya* and *Rouya*), and (2) 144 accessions of the *D. carota* complex and six accessions of *D. syrticus*. Moreover, 889,445 and 789,311 SNPs were obtained, respectively for each data set. Then, after filtering out SNPs using different criteria (see Methods), our final data sets with a maximum missing data of 10% consisted of 10,814 or 18,565 SNPs, respectively (Additional file 2: Table S2).

### Phylogeny inference

The maximum likelihood (ML) phylogenetic reconstruction of *Daucus* with 10 or 30% missing imputed genotypes allowed us to determine the outgroup of the *D. carota* complex to be *D. syrticus* (Additional file 3: Figure S1 and Additional file 4: Figure S2), fully in agreement with prior molecular studies in *Daucus* [27, 35, 36, 88–90]*.* Our ML trees, using varying numbers of SNPs, are highly resolved with two main clades, A and B, both with more than 97% bootstrap support (Additional file 3: Figure S1 and Additional file 4: Figure S2). Members of the *D. carota* complex together with *D. syrticus* possess 2*n* = 18 chromosomes, while the remaining taxa in clades A and B possess 2*n* = 20 and 22 chromosomes. Maximum likelihood trees show that species *Rouya polygama* (corrected from *Margotia gummifera* in Spooner et al. [35] and Arbizu et al. [36]) is placed within a monophyletic *Daucus* clade and is sister to a clade formed by the *D. carota* complex, and *D. syrticus*. In addition, *D. aureus* is sister to the clade mentioned above, and sister to them is *D. crinitus*. Members of the *D. guttatus* complex were placed within clade B. *Daucus involucratus* is sister to a clade formed by *D. guttatus* and *D. littoralis* (Additional file 3: Figure S1 and Additional file 4: Figure S2). Using 10,814 SNPs (10% missing imputed genotypes), *D. setulosus* is sister to *D. pusillus*. On the other hand, the ML tree using a data set containing 38,920 SNPs (30% missing imputed genotypes) places *D. pusillus* as sister to a clade formed by *D. guttatus*, *D. littoralis*, *D. involucratus*, and *D. setulosus*. For both data sets of missing imputed genotypes (10 or 30%), bootstrap values of most clades were higher than 90%. However, the data set containing 38,920 SNPs showed higher bootstrap support for the following three clades of *D. carota*: (i) Balkan Peninsula and Middle East, wild, (ii) North America and Europe, wild; and (iii) North Africa, wild (Additional file 3: Figure S1 and Additional file 4: Figure S2).

**Fig. 1:**
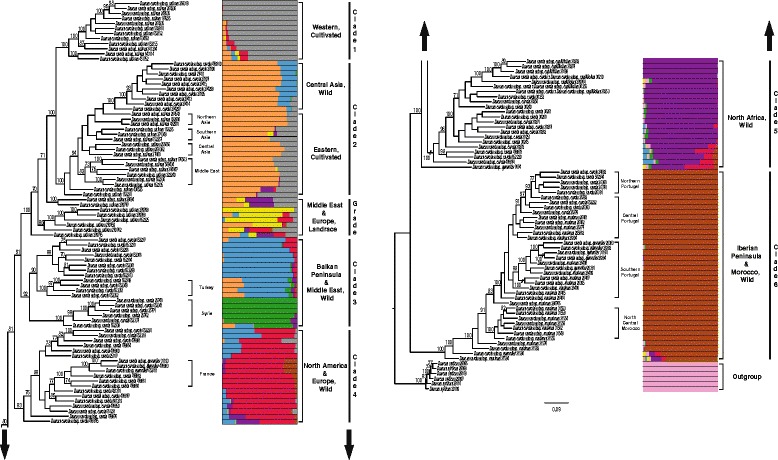
Maximum likelihood reconstruction and structure of the genetic diversity of 144 accessions of the *Daucus carota* complex and outgroups using 18,565 SNPs (10% missing imputed genotypes) obtained by GBS. Each accession is represented by a horizontal bar, and each color corresponds to a population (nine in total). Numbers above the branches represent bootstrap values, with only values higher than 70% shown. Names given to clades refer to the geographic origin and improvement status of the accessions of the *D. carota* complex. The outgroup taxon is *D. syrticus*

Employing data sets including only accessions of the *D. carota* complex, with *D. syrticus* as outgroup and using 18,565 and 43,713 SNPs with a maximum of 10% (Fig. 1) and 30% (Additional file 5: Figure S3) missing imputed genotypes, respectively, ML analyses revealed that within clade A′, members of the *D. carota* complex were placed in six clades (labeled clades 1–6 in Fig. 1 and Additional file 5: Figure S3) according to their cultivation type and geographic locality: (1) Western, cultivated, (2) central Asia, wild and Eastern, cultivated, (3) Balkan Peninsula and Middle East, wild, (4) North America and Europe, wild (5) North Africa, wild, and (6) Iberian Peninsula and Morocco, wild. The landrace accessions from the Middle East and Europe form a grade between clades 2 and 3.

We followed the same nomenclature used by Iorizzo et al. [33, 69] for western cultivated carrots (Europe and American continent) and eastern cultivated carrots (Middle East and Asia). All cultivated carrots (*D. carota* subsp. *sativus*) were monophyletic (clades 1,2 + grade; Fig. 1), but wild members of *D. carota* from central Asia were placed together with eastern members of subsp. *sativus*. Consequently, our study does not recover a subsp. *sativus* monophyletic clade as was reported in Iorizzo et al. [33], but it is concordant with the phylogenetic result of Iorizzo et al. [69]. Interestingly, four geographically coherent sub-clades (northern Asia, southern Asia, central Asia and Middle East) of clade 2 containing cultivated accessions were recovered (Fig. 1 and Additional file 5: Figure S3). However, two accessions, PI 430525 (Afghanistan) and PI 163234 (India), depicting extensive allelic admixture were not placed in those four sub-clades.

Maximum likelihood analyses using both data sets of 10 or 30% missing imputed genotypes recovered, with 100% bootstrap, two individual sub-clades of clade 3 containing accessions collected in Turkey and Syria (Fig. 1 and Additional file 5: Figure S3). Wild accessions of *D. carota* from France were also placed with 100% bootstrap support in a single sub-clade within the North America and Europe, wild clade (clade 4), depicting a separation between accessions of subspecies *carota* and *gummifer* (Fig. 1 and Additional file 5: Figure S3). All accessions of *D. carota* subsp. *capillifolius* collected in Tunisia and immediately adjacent western Libya are in the clade of subsp. *carota* from North Africa, wild.

Accessions PI 478873 and PI 478874 collected in Sardinia and Sicily (Italy), respectively, were on the North Africa, wild clade (clade 5). Both islands are located close to North Africa. Moreover, accession Ames 7674, donated from Tuscany (Italy) is not placed within the North America and Europe, wild clade (like in Additional file 3: Figure S1 and Additional file 4: Figure S2). Rather, it is sister to a clade formed by all members of the *D. carota* complex, except the clade placing those *D. carota* accessions from the Iberian Peninsula and Morocco, wild (Fig. 1 and Additional file 5: Figure S3).

All accessions from Portugal and Morocco that were initially named by GRIN as *D. carota* subsp. *maximus* and subsp. *maritimus* were on the Iberian Peninsula and Morocco, wild clade (clade 6); except PI 652225 which is in the Middle East and Europe, landrace grade. In addition, all subsp. *gummifer* from Portugal (except A26381) were on a sub-clade with 100 and 98% bootstrap support using 10 or 30% missing imputed genotypes, respectively (Fig. 1 and Additional file 5: Figure S3). Accessions from Portugal were placed, according to their geographic areas, in three sub-clades: northern Portugal, central Portugal and southern Portugal. There are, however, a few exceptions: (i) accession PI 652222, which was collected in Vila Real (north of Portugal) was not contained in the northern Portugal sub-clade but in the central Portugal sub-clade, and (ii) accession PI 295862 (collected in Spain) was placed in the central Portugal sub-clade for ML tree obtained using 10% missing imputed genotypes. The northern Portugal sub-clade has a bootstrap support of 77 and 99% for data set using 18,565 and 43,713 SNPs, respectively. (Fig. 1 and Additional file 5: Figure S3). Accessions collected in the north central region of Morocco were on a sub-clade with 100% bootstrap support for both data sets used in our ML analyses. Accession PI 280706 collected in Chile was on the Iberian Peninsula and Morocco, wild clade (Fig. 1 and Additional file 5: Figure S3). Bootstrap supports were higher than 90% for both data sets for the six clades mentioned above (Fig. 1 and Additional file 5: Figure S3).

As expected, with almost all posterior probability values of one, our Bayesian tree (Additional file 6: Figure S4) has the same overall topology as the ML tree (Fig. 1), including the cultivation status and geographic locality of the accessions.

The SVDquartets analyses using 18,565 and 43,713 SNPs with a maximum of 10 or 30% missing imputed genotypes, respectively, also recovered the same six clades of the *D. carota* complex (Additional file 7: Figure S5 and Additional file 8: Figure S6). There are, however, some differences with the trees obtained using a concatenated approach: (1) clades North Africa, wild and North America, wild are sisters, (2) the lineage tree using 43,713 SNPs (Additional file 8: Figure S6) shows wild carrots from central Asia and cultivated carrots (subsp. *sativus*) from eastern did not form a clade. Rather, with a low bootstrap value of support of 41%, the clade of western cultivated carrots was sister to the members of wild carrots from central Asia, (3) using data sets with 43,713 and 18,565 SNPs, wild carrots from central Asia form an individual clade with 97 and 54%, respectively. However, the data set using 43,713 SNPs did not include accession PI 274297 (collected in Pakistan), and (4) lineage trees using the two data sets misplaced accessions relative to the dominant tree topologies obtained by ML analyses. The most notable case is that the subsp. *gummifer* clade from France is sister to the clade containing other accessions of the *D. carota* complex. Both data sets show that bootstrap values are higher than 90% for most of the clades of the *D. carota* complex. In addition, accessions from Turkey were in a clade with 100 and 94% bootstrap support on lineage tree using 18,565 and 43,713 SNPs, respectively (Additional file 7: Figure S5 and Additional file 8: Figure S6). With a bootstrap support of 50%, the Syria sub-clade is recovered using 18,565 SNPs, but it is not fully recovered when 43,713 SNPs were used since accession PI 652338 is not contained in the Syria sub-clade. Rather, this accession was sister to the Turkey sub-clade (Additional file 8: Figure S6). Wild accessions of *D. carota* subsp. *carota* collected in France form a clade in both data sets. The three sub-clades containing accessions collected in Portugal named north, central and south were also obtained in the SVDquartets analyses, but accessions PI 26404 and PI 26405, both collected in southern Portugal, were not placed in the clade expected.

Utilizing a coalescent approach consisting of 18,565 SNPs, the species tree (Additional file 9: Figure S7) indicates that *D. carota* subsp. *capillifolius* was sister to a clade formed by subsp. *carota* and subsp. *sativus;* and sister to them, a clade formed by subsp. *gummifer* and subsp. *maximus*. The species tree using 43,713 SNPs (figure not shown in this study) demonstrated the same topology as mentioned above, but with a higher bootstrap value for the clade containing subsp. *carota* and *sativus:* 100 vs. 53%.

### Population structure

The Evanno method [84] indicated the best K value (number of populations) is two for both data sets. However, the next largest peak is at K = 9 and K = 3 for data matrix of 18,565 SNPs and 43,713 SNPs, respectively. Further, the third highest peak for data set with 43,713 SNPs (30% missing imputed genotypes) was obtained at K = 9 (Additional file 10: Figure S8 and Additional file 11: Figure S9). Waples and Gaggiotti [91] and Frantz et al. [92] indicate that the Evanno method tends to underestimate the number of genetic clusters. Other studies [33, 93] similarly obtained a false highest peak at K = 2 with the Evanno method due to a strong rejection of the null hypothesis of no structure (K = 1). Hence, the second highest peak obtained with 43,713 SNPs (K = 3) is likely caused by a strong rejection of the hypothesis of two clusters only. On the contrary, the highest likelihood value was obtained at K = 9 for both data sets (Additional file 10: Figure S8 and Additional file 11: Figure S9), which is concordant with the ML analyses of the *Daucus carota* complex (Fig. 1 and Additional file 5: Figure S3); so further discussion follows K = 9. STRUCTURE analyses show abundant admixture (Fig. 1 and Additional file 5: Figure S3), except for all accessions placed in cluster Iberian Peninsula and Morocco wild, which exhibit very low admixture. The *D. carota* complex is clustered according to wild vs. cultivated and its geographic origin. In addition, eastern members of subsp. *sativus* (cultivated carrot) are clustered with wild carrots from central Asia, rather than with all cultivated carrots. Furthermore, wild carrots from Syria also form a single cluster. All accessions of *D. syrticus* also cluster together and show no intraspecific allelic admixture (Fig. 1 and Additional file 5: Figure S3).

### Morphological analysis

Graphical analyses of the 23 character state distributions used in our morphological study are shown in Additional file 12: Figure S10. This demonstrates tremendous variation and overlap of character states across all taxa, similar to the results obtained by Spooner et al. [31] and Arbizu et al. [29]. Hence, only a multivariate analysis using a combination of morphological characters distinguishes members of the *D. carota* complex (except subsp. *capillifolius*). The following 11 out of the 23 continuous morphological characters were significant in the *F* test for this analysis of *D. carota*, *P* ≤ 0.05 in the stepwise discriminant analysis (from most to least significant): plant height, mericarp length, number of spines on the four secondary mericarp ribs, secondary umbel diameter, number of bract lobe pairs, stem diameter, peripheral petal length, petiole diameter, number of umbel rays, leaf-sheath width, and leaf length including petiole.

**Fig. 2:**
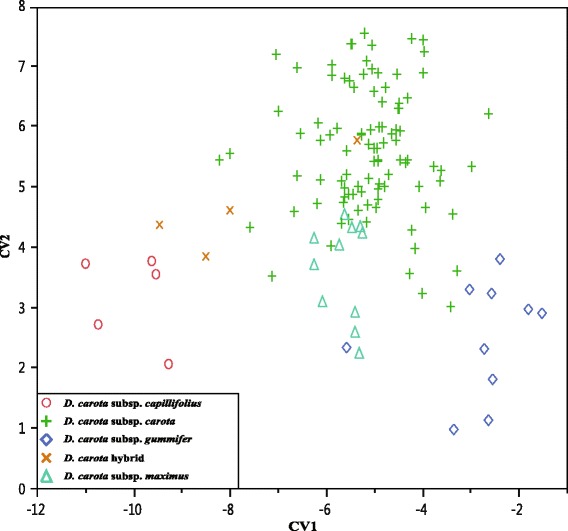
Canonical variate analysis of the *Daucus carota* complex (subsp. *sativus* not considered) using 23 continuous morphological characters from the stem, leaf, flower, and mericarp structures

**Fig. 3:**
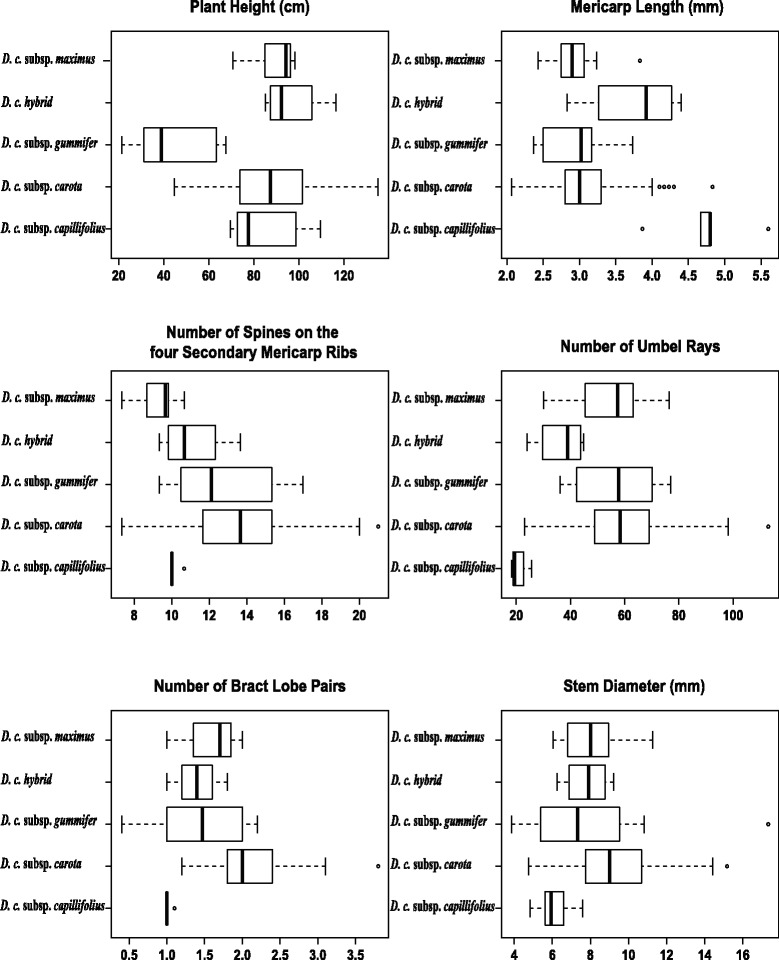
Box plots of six significant morphological characters obtained by canonical variate analysis that distinguish subspecies of the *Daucus carota* complex (subsp. *sativus* not considered). The box plots display individual plant values for median, 25 and 75% percentile, range, and outliers

Canonical variate analysis shows that all taxa are separated into four groups (*D. carota* subsp. *capillifolius*, subsp. *carota*, subsp. *gummifer*, and subsp. *maximus*). Accessions of *D. carota* hybrid are intermingled within the subsp. *carota* and subsp. *capillifolius* group. In addition, there are one and three accessions of subsp. *gummifer* and subsp. *carota*, respectively mixed with the subsp. *maximus* group (Fig. 2). Character state distributions of six significant discriminators are presented in Fig. 3, and character state overlap within these discriminators was very common.

### Key to *Daucus carota* subsp. *capillifolius,* subsp. *carota*, subsp. *gummifer*, subsp. *maximus* and subsp. *sativus*

Note: As discussed in the text, there is tremendous overlap in the ranges of these key morphological data. These character state ranges are from Spooner et al. [31] reanalyzed here, considering subsp. *maximus* as a valid subspecies (with the caveat that a few accessions of subsp. *carota* are on this clade 6), and using only the interquartile ranges of the data (Fig. 3 and Additional file 12: Figure S10).

1. Mature root not woody and edible, and highly pigmented (rarely white), root branching generally absent.........................subsp. *sativus*.
2. Mature root woody and inedible, white to white-yellow, roots commonly branched.
  2. Petal color yellow, number umbel rays generally 18–25, mericarp length 4.5–5.5 mm...............................subsp. *capillifolius*.
  2. Petal color white, number of umbel rays generally 40–70, mericarp length 2–4 mm.
    3. Plant height 25–60 cm, mature primary umbel shape generally flat, mainly growing in maritime environments, leaves shiny and thick with broad segments................................subsp. *gummifer*.
    3. Plant height 70–110 cm, mature primary umbel shape generally convex, mainly growing in continental environments, leaves dull and thin with narrow segments.
      4. Leaf-sheath width 7.5–10 mm, bract 30–45 mm x 30–45 mm, number of spines on the secondary mericarp ribs 11–15, length of secondary mericarp spines 1.0–1.5 mm ...........subsp. *carota*.
      4. Leaf-sheath width 5.5–7.0 mm, bract 20–35 mm x 20–35 mm, number of spines on the secondary mericarp ribs 8–10, length of secondary mericarp spines 1.5–2.0 mm.........subsp. *maximus*.

## Discussion

### Genotyping-by-sequencing data sets

Genome-wide data sets provide an opportunity to resolve difficult phylogenetic problems on species groups [94] that have failed using gene sequence phylogenies [45, 95, 96]. We here investigated the utility of SNPs obtained through GBS to resolve the classification of the *D. carota* complex. As expected, GBS identified abundant genome-wide SNPs (Additional file 2: Table S2), with varying levels of missing genotype calls. Due to low coverage sequencing, missing data has been reported as a problem in GBS [51, 97]. The impact of missing data on phylogenetic analyses has been extensively analyzed. Some studies [98–100] concluded that as long as the number of characters is abundant, phylogenetic inference is not sensitive to missing data. Other studies [101–107] concluded that missing data might affect phylogenetic reconstruction. Missing data can be addressed with imputation [108], a statistical method that replaces missing data by estimated values [97]. Yang et al. [109], using simulated data, reported that the accuracy of Beagle imputation software is 0.99 for a missing rate of 10 or 30%. Therefore, to explore further the utility of missing imputed genotypes on phylogenetic analysis, we employed missing rates of 10 or 30%.

### Phylogenomics of *Daucus*

The phylogenetic relationships of *Daucus* were previously reported by Spalik and Downie [88], Spalik et al. [89], Spooner et al. [35], Arbizu et al. [36, 90], Lee and Park [27], and Banasiak et al. [37]; all findings supported two main clades. Our two data sets with 10 or 30% missing imputed genotypes also reconstructed the two main clades of *Daucus*, A and B (Additional file 3: Figure S1 and Additional file 4: Figure S2). Significant differences were not observed between the two *Daucus* topologies using two data sets (10,914 SNPs vs. 38,920 SNPs), even though the data set with 10% missing imputed genotypes had 72% fewer SNPs. Arbizu et al. [36] also mentioned that a reduction in the amount of DNA sequence data did not influence the topology of *Daucus*. However, bootstrap values of some clades using 30% missing imputed genotypes (38,920 SNPs) were higher. Kimball and Braun [110] also indicated that they obtained a modestly greater bootstrap support by increasing their number of sites approximately 3-fold.

Similar to the consensus trees of *Daucus* obtained by Spooner et al. [35] and Arbizu et al. [36, 90], clade A in our ML tree consists of the *D. carota* complex, *D. syrticus*, *D. aureus*, *D. crinitus* and a related ingroup genus (*Rouya*). Concordant with these results, *D. syrticus* is the appropriate outgroup for the study of the *D. carota* complex. In addition, clade B consists of species of the *D. guttatus* complex and close relatives. Maximum likelihood analyses confirmed accessions of *D. guttatus* were recovered as a monophyletic clade as similarly reported in Arbizu et al. [90]. Originally, accession numbers A25731, A25732 and A25778 were identified by the GRIN database as *D. carota*. However, based on our ML results and morphological analysis of members of the *D. guttatus* complex [90, 111], these three accessions were re-identified as *D. guttatus*. The paraphyly of the genus *Daucus* (circumscribed containing *Rouya polygama*) was extensively documented using plastid or ribosomal DNA characters [35, 37]. Recent studies [37, 112], using nrDNA ITS sequences and plastid sequences also showed that *Rouya polygama* groups within the *Daucus* clade.

### Classification of the *D. carota* complex

*Daucus carota* sensu lato is a taxonomically complicated group that has attracted the attention of many investigators. Previous molecular and morphological studies have failed to distinguish many of the subspecies of the *D. carota* complex. Our present GBS data using imputed data for missing rates of 10 or 30% recovered a clade containing the members of the *D. carota* complex and *D. syrticus,* all consisting of 2*n* = 18 chromosomes, matching the results of prior investigators. Furthermore, concordant with previous work conducted by Iorizzo et al. [33, 69], our Bayesian and ML trees partitioned accessions of *D. carota* according to their improvement status (cultivated, landrace, wild) and geographic origin. We here identified one grade and one extra clade, respectively: (1) Middle East and Europe, landrace, and (2) Iberian Peninsula and Morocco, wild (Fig. 1 and Additional file 5: Figure S3). Using 3,326 SNPs in expressed genes, Iorizzo et al. [33] indicated that eastern wild accessions of carrots (i.e., collected in the Middle East, central Asia and eastern Asia) are most closely related to cultivated carrots. Our present data, using additional samples from more diverse geographic origins and all genomic regions rather than only expressed genes, revealed that eastern members of subsp. *sativus* (cultivated carrot) are in a clade with wild carrots from central Asia, rather than with all cultivated carrots as shown in the ML tree of Iorizzo et al. [33]. Considerable allelic admixture shown by STRUCTURE results is in agreement with the cross-fertilization habit within carrots and the introgression between wild populations and cultivated carrots [113–115]. Even though China and Pakistan geographically do not belong to the central Asia region, our study placed two accessions collected in northern Pakistan (Parachinar) and one in northwestern China (Xinjiang) in the central Asia wild clade, yet these collections are very close to the central Asia region. Future work will benefit from extra samples from neighboring countries of the central Asia geographic region. Rong et al. [26] also used SNPs and samples of wild carrots from different geographic origins except central Asia, and their Bayesian tree showed that wild carrots from western Asia are the closest relatives to eastern cultivated carrots.

Extensive morphological studies conducted by Small [3], Reduron [116], Arbizu et al. [29] and Spooner et al. [31] identified two comprehensive morphological groups of wild *D. carota* (*carota* and *gummifer*). The latter authors proposed two morphologically supported taxa for the *D. carota* complex, *D. carota* subsp. *carota* and subsp. *gummifer*. However, these subspecies have not been recognized by a recent molecular study [36]. Similarly, the present study failed to identify two unique groups, even though we used genome-wide SNPs and 144 samples of *D. carota*.

Using fruit morphological descriptors, Mezghani et al. [30] separated subsp. *maximus* from the other subspecies of *D. carota*. An investigation [32] attempting to distinguish the subspecies of *D. carota* native to Portugal provided further evidence to separate subsp. *maximus* from other taxa by morphometric analysis of the fruits and chemical characterization of essential oils, concluding, as Sáenz de Rivas and Heywood [117], that subsp. *maximus* should be considered a species (i.e., *D. maximus*) rather than a subspecies of *D. carota*. Our discriminant analysis also separated subsp. *maximus* from the other subspecies *of D. carota*, but there are no individual characters that alone affect this separation and they extensively overlapped with other subspecies (Additional file 12: Figure S10). These facts, and the intercrossability of subsp. *maximus* with the other subspecies, and all cladistics analyses to date, lead us to retain ‘*maximus*′ as a provisional subspecies of *D. carota*, awaiting further studies using additional collections. Arbizu et al. [29] made the same case to recognize *D. capillifolius* as a subspecies of *D. carota*.

A single clade exclusively containing *D. carota* subsp. *carota* was not obtained. Rather, at 100% bootstrap value or posterior probability of 1, we observed a single clade containing cultivated carrot (subsp. *sativus*), and the wild subspecies *capillifolius*, *carota* (except those from northern and central Portugal), and *gummifer* from France. Similar to Iorizzo et al. [33], subsp. *carota* clade was subdivided according to their geographic origins as follows: (i) central Asia, wild, (ii) the Balkan Peninsula and the Middle East, wild (iii) North America and Europe, wild, (iv) North Africa, wild, and (v) Iberian Peninsula and Morocco, wild. Our ML and STRUCTURE results demonstrate that eastern cultivated carrots originated from populations of *D. carota* from central Asia.

A strict concept for monophyly failed for the subspecies of the *D. carota* complex. *Daucus carota* subsp. *gummifer*, used here is a broad sense to include other potential subspecies, as outlined in Spooner et al. [31] is a morphologically coherent set of morphotypes restricted to areas within a few km of the Atlantic Ocean and the Mediterranean Sea [31], and subsp. *capillifolius* is a morphologically distinctive taxon from Tunisia and Libya. Subspecies *capillifolius* occurs within the clade containing subsp. *carota*. Moreover, subsp. *gummifer* occurs in different clades containing geographically isolated populations of subsp. *carota* in Europe (France, Portugal, and Italy) and subsp. *maximus* in northern Africa (Morocco). GBS results, therefore, do not support the subspecies of *D. carota* to form monophyletic lineages, except subsp. *capillifolius*, which is an apospecies (sensu Olmstead [118]).

Accessions originally labeled as *D. carota* subsp. *maritimus* were placed in the Iberian Peninsula and Morocco, wild clade. Arbizu et al. [36], using 94 nuclear orthologs, also reported that accessions of *D. carota* from Portugal, Spain and Morocco were placed in an individual sub-clade with 100% bootstrap support. Rong et al. [26] marked one group of their Bayesian tree containing subspecies of *D. carota* as “Mediterranean, southern Europe”. Within this group, there is a sub-clade consisting of samples from Portugal and Spain labeled subsp. *carota*, subsp. *maritimus* and subsp. *maximus.* However, our results do not distinguish them from other subspecies (subsp. *azoricus* and subsp. *hispanicus* not examined here). Spooner et al. [31] considered subsp. *maritimus* and subsp. *major* in the *D. carota* subsp. *carota* group. Similarly, Bolòs and Vigo [119] and Pujadas Salvà [19] considered that subsp. *maritimus* corresponded to subsp. *carota*. Consequently, we suggest that morphotypes labeled as subsp. *carota,* subsp. *major* and subsp. *maritimus* from the Iberian Peninsula are subsp. *carota*. In addition, we consider that accessions from the Iberian Peninsula and Morocco originally labeled as subsp. *maximus* represent this subspecies since our morphological analysis distinguish them from subsp. *carota*. Within Portugal, it is very likely that subsp. *carota* predominates in the northern area of the country, while subsp. *maximus* is restricted to parts of central and southern Portugal and Morocco.

Using nuclear ribosomal DNA ITS, Lee and Park [27] grouped subsp. *halophilus*, subsp. *azoricus*, subsp. *gadecaei*, subsp. *drepanensis* and subsp. *gummifer* into a single clade. The last two subspecies correspond to subsp. *gummifer* [31] and in the present study accession Ames 31194, initially labeled as subsp. *halophilus*, is placed together with other accessions of subsp. *gummifer*. Matias Vaz [28] also reported that samples from São Miguel Island are more closely related to subsp. *gummifer* and subsp. *halophilus*. Therefore, we propose subsp. *azoricus* should be considered as *D. carota* subsp. *gummifer*. Another subspecies of *D. carota*, subsp. *majoricus* (A. Pujadas) is distributed in the Mallorca, Cabrera and surrounding islands [19], and based on the detailed morphological description provided by Martínez-Flores et al. [120], we consider it very likely this subspecies also corresponds to the subsp. *gummifer*.

Lineage trees, obtained using 10 or 30% missing imputed genotypes and a very fast quartet-based method under the coalescent model implemented in SVDquartets software [78], show differences in the topology compared to Bayesian and ML trees. Discordance between ML and quartet phylogenies of *Carex* sect. *Racemosae* also were reported by Massatti et al. [49]. A primary concordance tree indicated that the subspecies of *D. carota* have low concordance factors [36], suggesting high discordance between genes in wild carrots; probably caused by recombination, hybridization and introgression [41], and incomplete lineage sorting (ILS) [44]. Concatenation methods are often more accurate than coalescent-based methods if ILS is low [121–123]. Therefore, perhaps the main cause of discordance between genes of the subspecies of *D. carota* is not high ILS, but other factors. A species tree using HKY model of evolution reported by Arbizu et al. [36] shows that subsp. *carota* is sister subsp. *gummifer*, and sister to them is subsp. *capillifolius*. In the present study, we obtained a similar species tree (Additional file 9: Figure S7). The difference is that subsp. *maximus* was considered as a distinct subspecies in the present study.

Cultivated carrot (*D. carota* subsp. *sativus*) is the most important member in the Apiaceae family in terms of economy and nutrition [115, 124, 125], and is considered the second most popular vegetable in the world after potato [126]. Genetic diversity of wild carrots can be exploited to meet future challenges such as nutraceutical food for a growing population, cultivars adapted to climate change, among others. Wild *Daucus* species possessing 2*n* = 18 chromosomes represent the main gene pool for breeding work in carrots. Here we also propose that wild carrots (subsp. *maximus*) from the Iberian Peninsula and Morocco may be used as a new source of genes for the development of new carrot cultivars. Unique essential oils are present in *D. carota* subsp. *maximus* [32, 127] and are considered to confer insecticidal properties. Indeed, this compound may be useful in new carrot commercial hybrids for organic production system.

## Conclusions

Single nucleotide polymorphisms (SNPs) provide robust markers for the study of population structure and phylogenetics of *Daucus carota*. Genotyping-by-sequencing identified numerous SNPs distributed along the nine chromosomes of *D. carota*. Our data sets with 10 or 30% missing data with imputed data additions tested the utility of genome-wide SNPs in clarifying the phylogenetics and population structure of *D. carota* and related species. The phylogenomics of *Daucus* was well resolved, showing higher bootstrap support for most clades using the GBS data set with a larger number of SNPs, 38,920 sites (30% missing imputed data). However, both data sets used to study the *D. carota* complex (18,565 SNPs vs. 43,713 SNPs) revealed equal topology and very similar bootstrap values for most clades. One new clade and one grade were detected (Iberian Peninsula and Morocco wild, and Middle East and Europe landrace). The Bayesian structure results are concordant with the maximum likelihood tree, except that an extra cluster consisting of wild carrots from Syria was detected (nine in total). Wild carrots from central Asia were more related to eastern cultivars further supporting the hypothesis that cultivated carrot originated in central Asia. Low allelic admixture was shown for wild carrots belonging to the Iberian Peninsula and Morocco clade. The morphologically distinct wild carrot subsp. *capillifolius*, from Tunisia and immediately adjacent Libya, was placed in a sub-clade of subsp. *carota* from North Africa and is supported as an apospecies. *Daucus carota* subsp. *gummifer* is a morphologically coherent set of morphotypes restricted to areas within a few km of the Atlantic Ocean and the Mediterranean Sea. It is here supported as a series of apospecies with separate and independent origins from subsp. *carota* and subsp. *maximus* in similar maritime habitats; the nomenclature of these awaits further study. Discriminant analysis distinguished 11 significant morphological descriptors that best distinguish the subspecies of the *D. carota* complex. Phylogenies obtained by Bayesian and ML method were very similar. However, these concatenation approaches showed disagreement with a coalescent-based method probably due to varying ILS levels in the genome of the subspecies of *D. carota*. Our study demonstrates the ability of GBS to distinguish population and subspecies structure in *D. carota*. Further studies using additional collections of *D. carota* from a wider geographic area and other potential subspecies (these field collections are in progress), and examination of relevant type material, are needed to provide a better understanding of taxonomic variation and nomenclature in the *D. carota* complex. This was the very process by which Arbizu et al. [90] and Martínez-Flores et al. [111] solved the species boundaries in another problematical group in *Daucus*, a procedure we are pursuing here.

## Additional files

Additional file 1: Table S1. The 162 accessions of *Daucus*, and two accessions of related genera characterized in this study, improvement status, locality information and new identification. (PDF 308 kb)
Additional file 2: Table S2. Summary of processing GBS data of *Daucus*. (PDF 98 kb)
Additional file 3: Figure S1. Phylogenomics of *Daucus* from a maximum likelihood analysis using 164 accessions and 10,814 SNPs (10% missing imputed genotypes) obtained by GBS. Numbers above branches represent bootstrap values, with only values higher than 70% shown. Names given to clades refer to the geographic origin and improvement status of the accessions of the *D. carota* complex. Clades A and B corresponds to the two main groups of the *Daucus* phylogeny. (PDF 1.39 mb)
Additional file 4: Figure S2. Phylogenomics of *Daucus* from a maximum likelihood analysis using 164 accessions and 38,920 SNPs (30% missing imputed genotypes) obtained by GBS. Numbers above branches represent bootstrap values, with only values higher than 70% shown. Names given to clades refer to the geographic origin and improvement status of the accessions of the *D. carota* complex. Clades A and B corresponds to the two main groups of the *Daucus* phylogeny. (PDF 1.34 mb)
Additional file 5: Figure S3. Maximum likelihood reconstruction and structure of the genetic diversity of 144 accessions of the *Daucus carota* complex and outgroups using 43,713 SNPs (30% missing imputed genotypes) obtained by GBS. Each accession is represented by a horizontal bar, and each color corresponds to a population (nine in total). Numbers above branches represent bootstrap values, with only values higher than 70% shown. Names given to clades refer to the geographic origin and improvement status of the accessions of the *D. carota* complex. The outgroup taxon is *D. syrticus*. (PDF 1.46 mb)
Additional file 6: Figure S4. Bayesian phylogenetic tree of 144 accessions of the *Daucus carota* complex and outgroups using 18,565 SNPs (10% missing imputed genotypes) obtained by GBS. Numbers above the branches represent posterior probabilities, with only values higher than 0.7 shown. Names given to clades refer to the geographic origin and improvement status of the accessions of the *D. carota* complex. The outgroup taxon is *D. syrticus*. (PDF 1.33 mb)
Additional file 7: Figure S5. Relationships among 144 accessions of the *Daucus carota* complex and outgroups from an exhaustive quartet sampling inference using 18,565 SNPs (10% missing imputed genotypes) obtained by GBS. Numbers above the branches represent bootstrap values, with only values higher than 70% shown. Names given to clades refer to the geographic origin and improvement status of the accessions of the *D. carota* complex. ME & E refers to Middle East & Europe. Accessions designated by double stars are misplaced relative to the maximum likelihood topology of the *Daucus carota* complex using the same number of SNPs. The outgroup taxon is *D. syrticus*. (PDF 1.30 mb)
Additional file 8: Figure S6. Relationships among 144 accessions of the *Daucus carota* complex and outgroups from an exhaustive quartet sampling inference using 43,713 SNPs (30% missing imputed genotypes) obtained by GBS. Numbers above branches represent bootstrap values, with only values higher than 70% shown. Names given to clades refer to the geographic origin and improvement status of the accessions of the *D. carota* complex. ME & E refers to Middle East & Europe. Accessions designated by double stars are misplaced relative to the maximum likelihood topology of the *Daucus carota* complex using the same number of SNPs. The outgroup taxon is *D. syrticus*. (PDF 142 kb)
Additional file 9: Figure S7. Species tree of the *Daucus carota* complex based on a coalescent model using an exhaustive quartet sampling inference and 18,565 SNPs (10% missing imputed genotypes) obtained by GBS. Numbers above the branches represent bootstrap values. The outgroup taxon is *D. syrticus*. (PDF 104 kb)
Additional file 10: Figure S8. Number of populations. A. Plot of Delta K (ΔK). B. Plot of the log likelihood; internal plot corresponds to the log likelihood (thousands) for K ranging from 1 to 9. All values were obtained from STRUCTURE HARVESTER analysis. Fourteen populations were considered in a data set of 18,565 SNPs (10% missing imputed genotypes) and 150 samples. (PDF 893 kb)
Additional file 11: Figure S9. Number of populations. A. Plot of Delta K (ΔK). B. Plot of the log likelihood; internal plot corresponds to the log likelihood (thousands) for K ranging from 1 to 9. All values were obtained from STRUCTURE HARVESTER analysis. Fourteen populations were considered in a data set of 43,713 SNPs (30% missing imputed genotypes) and 150 samples. (PDF 167 kb)
Additional file 12: Figure S10. Box plot analyses of the 23 morphological characters examined for members of *Daucus carota* complex (subsp. *sativus* not included) in this study. The box plot displays individual plant values for median, 25 and 75% percentile, range, and outliers. (PDF 17.1 mb)
