# Supplementary material for: Genotyping-by-sequencing provides the discriminating power to investigate the subspecies of Daucus carota (Apiaceae)
Source: BMC Evol Biol. 2016 Oct 28;16:234. doi: 10.1186/s12862-016-0806-x (PMC5084430; doi:10.1186/s12862-016-0806-x)
Supplement: Additional file 1: Table S1. — The 162 accessions of Daucus, and two accessions of related genera characterized in this study, improvement status, locality information and new identification. (PDF 308 kb) [file 12862_2016_806_MOESM1_ESM.pdf]

Additional file 1: Table S1. The 162 accessions of *Daucus*, and two accessions of related genera characterized in this study, improvement status, locality information and new identification.

Accessions[a]	Taxon and 2*n* Chromosome Number[b]	Improvement Status	Location or Source[c]	Geographic Region	New Identification
PI 319403	*Daucus aureus* Desf. (22) +	Wild	Israel. Mediterranean Region.		
Ames 25731	*D. carota* L. (18, all subspecies) +	Wild	Syria. Al-Qastal: 30 km northeast of Al Ladhiqiyah.		*D. guttatus*
Ames 25732	*D. carota* +	Wild	Syria. Al-Qastal: 30 km northeast of Al Ladhiqiyah.		*D. guttatus*
Ames 25771	*D. carota*	Wild	Syria. Kfarya: Near Salma.	Middle East	*D. carota* subsp. *carota*
Ames 25778	*D. carota* +	Wild	Syria. Kasab.		*D. guttatus*
Ames 26404	*D. carota*	Wild	Portugal. Evora: Near Portel.	Iberian Peninsula	*D. carota* subsp. *maximus*
Ames 26405	*D. carota*	Wild	Portugal. Beja: Near Baleizão.	Iberian Peninsula	*D. carota* subsp. *maximus*
Ames 26406	*D. carota*	Wild	Portugal. Beja: Near Ourique.	Iberian Peninsula	*D. carota* subsp. *maximus*
Ames 26409	*D. carota*	Wild	Portugal. Faro: Near Salema.	Iberian Peninsula	*D. carota* subsp. *maximus*
Ames 27396	*D. carota*	Wild	Uzbekistan. On main road from Village of Sijjak.	Central Asia	*D. carota* subsp. *carota*
Ames 27400	*D. carota*	Cultivated	Uzbekistan. Tashkent.	Eastern	*D. carota* subsp. *sativus*
Ames 27411	*D. carota*	Wild	Uzbekistan.	Central Asia	*D. carota* subsp. *carota*
Ames 27413	*D. carota*	Wild	Uzbekistan. Chetsuv Village.	Central Asia	*D. carota* subsp. *carota*
Ames 27414	*D. carota*	Wild	Uzbekistan. Chetsuv Village.	Central Asia	*D. carota* subsp. *carota*
Ames 27415	*D. carota*	Wild	Uzbekistan. Chetsuv Village: Approximately 50 meters from Z10.	Central Asia	*D. carota* subsp. *carota*
Ames 29084	*D. carota*	Landrace	Tunisia.		*D. carota* subsp. *sativus*
Ames 30250	*D. carota*	Wild	Tunisia. Nabuel: along Route 28 at junction of road to Takelsa.	North Africa	*D. carota* subsp. *carota*
Ames 30252	*D. carota*	Wild	Tunisia. Nabuel: Sidi Daoud, 1 km from Route 27.	North Africa	*D. carota* subsp. *carota*

Ames 30260	*D. carota*	Wild	Tunisia. Bizerte: along Route 51, west of Ghzab.	North Africa	*D. carota* subsp. *carota*
Ames 30261	*D. carota*	Wild	Tunisia. Bizerte: grounds of Direction Regionale Mogods, Khroumerie Sejnane.	North Africa	*D. carota* subsp. *carota*
Ames 31548	*D. carota*	Wild	Morocco. Sefrou: Along Route N8, 24 miles north of Azrou, Oulad Mkoudou Region.	Morocco	*D. carota* subsp. *maximus*
Ames 31553	*D. carota*	Wild	Morocco. Fes: Along Route N6 (road to Taza from Fes), just east of deviation with Route N8, Sidi Harazem.	Morocco	*D. carota* subsp. *maximus*
Ames 31554	*D. carota*	Wild	Morocco. Fes: Along Route N6 (road to Taza), 13 kilometers from deviation with Route N8, 28 kilometers east of downtown Fes, Sidi Harazem Region.	Morocco	*D. carota* subsp. *maximus*
Ames 31555	*D. carota*	Wild	Morocco. Sefrou: Along Route N6, 28 kilometers from deviation with Route N8, 58 kilometers east of downtown Fes, Bir Tam Tam Region.	Morocco	*D. carota* subsp. *maximus*
Ames 31558	*D. carota*	Wild	Morocco. Sefrou: Along Route R503, south side of Fes, 6 kilometers north of Sefrou, Aghbalou Aqorar Region.	Morocco	*D. carota* subsp. *maximus*
Ames 31559	*D. carota*	Wild	Morocco. Meknes: Along Route N4, 36 kilometers west of Fes, east of Sidi Kacem, Mhaya Region.	Morocco	*D. carota* subsp. *maximus*
Ames 31562	*D. carota*	Wild	Morocco. Sidi Kacem: Along Route 410, 4 kilometers south of Mechra Belqsiri, Sefsaf Region.	Morocco	*D. carota* subsp. *maximus*
Ames 31570	*D. carota*	Wild	Morocco. Larache: approximately 10 kilometers south of Larache, Laouamra Region.	Morocco	*D. carota* subsp. *maximus*
Ames 31580	*D. carota*	Wild	Morocco. Nador: Along Route N16, 41 kilometers west of Nador, approximately 0.5 kilometer from Mediterranean	Morocco	*D. carota* subsp. *gummifer*

			Sea, Amejjaou Region.		
Ames 31584	*D. carota*	Wild	Morocco. Berkane: Along Route S403, approximately 5 kilometers northwest of road to Sidi Bouhria, Rislane Region.	Morocco	*D. carota* subsp. *maximus*
Ames 31592	*D. carota*	Wild	Morocco. Safi: 11 kilometers east of Safi on Route 204, Khatazakane Region.	Morocco	*D. carota* subsp. *maximus*
PI 163234	*D. carota*	Cultivated	India. Madhya Pradesh.	Eastern	*D. carota* subsp. *sativus*
PI 163235	*D. carota*	Cultivated	Pakistan. Punjab.	Eastern	*D. carota* subsp. *sativus*
PI 176563	*D. carota*	Cultivated	Turkey. Kutahya.	Eastern	*D. carota* subsp. *sativus*
PI 187235	*D. carota*	Cultivated	Belgium.	Western	*D. carota* subsp. *sativus*
PI 222249	*D. carota*	Cultivated	Iran. Tehran.	Eastern	*D. carota* subsp. *sativus*
PI 249535	*D. carota*	Cultivated	Spain.	Western	*D. carota* subsp. *sativus*
PI 256066	*D. carota*	Cultivated	Afghanistan. Kabul.	Eastern	*D. carota* subsp. *sativus*
PI 264235	*D. carota*	Cultivated	France.	Western	*D. carota* subsp. *sativus*
PI 264236	*D. carota*	Cultivated	France.	Western	*D. carota* subsp. *sativus*
PI 264543	*D. carota*	Cultivated	Japan. Osaka.	Eastern	*D. carota* subsp. *sativus*
PI 268382	*D. carota*	Cultivated	Afghanistan. Kabul.	Eastern	*D. carota* subsp. *sativus*
PI 269319	*D. carota*	Cultivated	Sweden.	Western	*D. carota* subsp. *sativus*
PI 271348	*D. carota*	Cultivated	India. Delhi.	Eastern	*D. carota* subsp. *sativus*
PI 279759	*D. carota*	Landrace	Spain. Madrid (Botanic Garden).		*D. carota* subsp. *sativus*
PI 279762	*D. carota*	Landrace	Denmark. Copenhagen.		*D. carota* subsp. *sativus*
PI 279798	*D. carota*	Landrace	Spain. Madrid (Botanic Garden).		*D. carota* subsp. *sativus*
PI 280706	*D. carota*	Wild	Chile. Concepcion.	South America	*D. carota* subsp. *maximus*
PI 341204	*D. carota*	Cultivated	France.	Western	*D. carota* subsp. *sativus*
PI 502914	*D. carota*	Cultivated	Germany.	Western	*D. carota* subsp. *sativus*
PI 634651	*D. carota*	Cultivated	USA. Minnesota.	Western	*D. carota* subsp. *sativus*
PI 643114	*D. carota*	Cultivated	USA. California.	Western	*D. carota* subsp. *sativus*
PI 652222	*D. carota*	Wild	Portugal. Vila Real: S. Joao da Pesqueira, Tras-os-Montes (Alto Douro) Province.	Iberian Peninsula	*D. carota* subsp. *carota*
PI 652237	*D. carota*	Wild	Bulgaria. Lovech: Balkan Mountains.	Balkan Peninsula	*D. carota* subsp. *carota*

PI 652257	*D. carota*	Cultivated	India. Delhi.	Eastern	*D. carota* subsp. *sativus*
PI 652301	*D. carota*	Wild	Greece. Ionian Islands: Englouvi.	Balkan Peninsula	*D. carota* subsp. *carota*
PI 652335	*D. carota*	Cultivated	Syria. Damascus.	Eastern	*D. carota* subsp. *sativus*
PI 652336	*D. carota*	Cultivated	Syria. Damascus.	Eastern	*D. carota* subsp. *sativus*
PI 652337	*D. carota*	Wild	Syria. Sweida.	Middle East	*D. carota* subsp. *carota*
PI 652338	*D. carota*	Wild	Syria. Sweida.	Middle East	*D. carota* subsp. *carota*
PI 652348	*D. carota*	Wild	Turkey. Izmir: West of Izmir.	Middle East	*D. carota* subsp. *carota*
PI 661219	*D. carota*	Wild	USA. Washington: South side of Route 507 (old Highway 99), just southwest of Tenino Avenue junction with Mina Acres Drive, Thurston County	North America	*D. carota* subsp. *carota*
PI 661231	*D. carota*	Wild	USA. California: Crescent City, Del Norte County.	North America	*D. carota* subsp. *carota*
Ames 26395	*D. carota*	Wild	Portugal. Faro: Near Fuseta.	Iberian Peninsula	*D. carota* subsp. *maximus*
Ames 27395	*D. carota*	Wild	Uzbekistan. Northeast of Gazelkent on main road to Tashkent.	Central Asia	*D. carota* subsp. *carota*
Ames 30267	*D. carota*	Wild	Tunisia. Jendouba: Road 11 between Ain Draham to Beja, near km 47.5.	North Africa	*D. carota* subsp. *carota*
PI 274298	*D. carota*	Wild	Pakistan. Four miles west of Parachinar.	Central Asia	*D. carota* subsp. *carota*
PI 344447	*D. carota*	Cultivated	Iran. Hamadan.	Eastern	*D. carota* subsp. *sativus*
PI 478874	*D. carota*	Wild	Italy. Sicily: Catania.	North Africa	*D. carota* subsp. *carota*
PI 652224	*D. carota*	Wild	Poland. Lomza.	Europe	*D. carota* subsp. *carota*
PI 652299	*D. carota*	Wild	Greece. Ionian Islands: Vasiliki.	Balkan Peninsula	*D. carota* subsp. *carota*
PI 652304	*D. carota*	Wild	Greece. Peloponnese: 10 km south of Patrai, toward Pyrgos.	Balkan Peninsula	*D. carota* subsp. *carota*
PI 652306	*D. carota*	Wild	Greece. Peloponnese: Near Koklas village, toward Sparti, Messenia Prefecture.	Balkan Peninsula	*D. carota* subsp. *carota*
PI 652349	*D. carota*	Wild	Turkey. Izmir: West of Izmir.	Middle East	*D. carota* subsp. *carota*

PI 652352	*D. carota*	Wild	Turkey. Izmir: Near Bağarasi.	Middle East	*D. carota* subsp. *carota*
Ames 30198	*D. carota* subsp. *capillifolius* (Gilli) Arbizu	Wild	Tunisia. Medenine: Djerba Island, Boumellel, 2 km north of Houmt Souk.	North Africa	
Ames 30202	*D. carota* subsp. *capillifolius*	Wild	Tunisia. Medenine: Djerba Island, just outside of south end of El May.	North Africa	
Ames 30207	*D. carota* subsp. *capillifolius*	Wild	Tunisia. Medenine: Road to El Kantara, south of Djerba Island.	North Africa	
PI 279764	*D. carota* subsp. *capillifolius*	Wild	Libya. Near Jefren.	North Africa	
Ames 25740	*D. carota* subsp. *carota*	Wild	Syria. As Samra.	Middle East	
Ames 25762	*D. carota* subsp. *carota*	Wild	Syria. Deir Mama.	Middle East	
Ames 26374	*D. carota* subsp. *carota*	Wild	Portugal. Porto: Near Vila do Conde.	Iberian Peninsula	*D. carota* subsp. *carota*
Ames 26377	*D. carota* subsp. *carota*	Wild	Portugal. Portalegre: Near Monforte.	Iberian Peninsula	*D. carota* subsp. *maximus*
Ames 26379	*D. carota* subsp. *carota*	Wild	Portugal. Portalegre: Near Marvao.	Iberian Peninsula	*D. carota* subsp. *carota*
Ames 27397	*D. carota* subsp. *carota*	Wild	Uzbekistan. Along main road between Yalangoch and Sobir Raximova.	Central Asia	
Ames 30245	*D. carota* subsp. *carota*	Wild	Tunisia. Zaghouan: Along road between Fahs and El Magrane.	North Africa	
Ames 30249	*D. carota* subsp. *carota*	Wild	Tunisia. Nabeul: Along Route 26 between Saliman and Sidi Aissa.	North Africa	
Ames 30251	*D. carota* subsp. *carota*	Wild	Tunisia. Nabuel: Route 26, between Takelsa and El Haouaria, 26 km from El Haouaria.	North Africa	
Ames 30262	*D. carota* subsp. *carota*	Wild	Tunisia. Beja: road from Route 7, just west of Sejnane to Cap Negro.	North Africa	
Ames 30271	*D. carota* subsp. *carota*	Wild	Tunisia. Beja: Road between Beja and Nefza.	North Africa	
Ames 30272	*D. carota* subsp. *carota*	Wild	Tunisia. Jendouba: South of Hotel Tabarka beach.	North Africa	
PI 274297	*D. carota* subsp.	Wild	Pakistan. Northern areas.	Central	

	carota			Asia	
PI 279775	*D. carota* subsp. *carota*	Landrace	Hungary. Pest (Botanic Garden).		*D. carota* subsp. *sativus*
PI 279788	*D. carota* subsp. *carota*	Landrace	Austria. Vienna (Museum of Natural History).		*D. carota* subsp. *sativus*
PI 295862	*D. carota* subsp. *carota*	Wild	Spain.	Iberian Peninsula	*D. carota* subsp. *maximus*
PI 421301	*D. carota* subsp. *carota*	Wild	USA. Kansas: Elk County.	North America	
PI 430525	*D. carota* subsp. *carota*	Cultivated	Afghanistan. Zardek.	Eastern	*D. carota* subsp. *sativus*
PI 478369	*D. carota* subsp. *carota*	Wild	China. Xinjiang: near Chou En Lai Monument Stone River, Sinkiang.	Central Asia	
PI 478859	*D. carota* subsp. *carota*	Wild	Italy. Misano Adriatico: Near Rimini.	Europe	
PI 478860	*D. carota* subsp. *carota*	Wild	France. Paris: Versailles Park, Seine et Oise.	Europe	
PI 478861	*D. carota* subsp. *carota*	Wild	France. Paris: Versailles Park, Seine et Oise.	Europe	
PI 478862	*D. carota* subsp. *carota*	Wild	France. Mere (Seine et Oise).	Europe	
PI 478864	*D. carota* subsp. *carota*	Wild	Germany (Botanic Garden).	Europe	
PI 478869	*D. carota* subsp. *carota*	Wild	Germany. Near Jüterbog.	Europe	
PI 478873	*D. carota* subsp. *carota*	Wild	Italy. Sardinia: St. Elia Beach, 50 m from sea, Cagliari	North Africa	
PI 478875	*D. carota* subsp. *carota*	Wild	Italy. Molise: Larino Province (Campobasso).	Europe	
PI 478876	*D. carota* subsp. *carota*	Wild	Italy. Latium (Botanic Garden).	Europe	
PI 478877	*D. carota* subsp. *carota*	Wild	Switzerland. Geneva: Vandœuvres.	Europe	
PI 478881	*D. carota* subsp. *carota*	Wild	USA. Oregon: roadside between Echo and Pendleton.	North America	
PI 652218	*D. carota* subsp. *carota*	Wild	Hungary. Near Bekes.	Europe	
PI 652296	*D. carota* subsp. *carota*	Wild	Greece. Epirus: 7 km from Ioannina toward Igoumenitsa.	Balkan Peninsula	
PI 652303	*D. carota* subsp. *carota*	Wild	Greece. Central Greece: Near Agrinion, toward Patrai.	Balkan Peninsula	

PI 652341	*D. carota* subsp. *carota*	Wild	Syria. Ash Sheik Hasan.	Middle East	
PI 652351	*D. carota* subsp. *carota*	Wild	Turkey. Izmir: Near Cesme.	Middle East	
Ames 30219	*D. carota* subsp. *carota* x *D. carota* subsp. *capillifolius*	Wild	Tunisia. Sidi Bouzid: along road to Sidi Bouzid (Route 125), north of Bir El Hfey, at El M'Zara.	North Africa	
Ames 30253	*D. carota* subsp. *carota* x *D. carota* subsp. *capillifolius*	Wild	Tunisia. Nabuel: Between El Haouarcae and Dor Allouche.	North Africa	
Ames 30255	*D. carota* subsp. *carota* x *D. carota* subsp. *capillifolius*	Wild	Tunisia. Nabuel: along road between Korba and Beni Khalled.	North Africa	
Ames 7674	*D. carota* subsp. *commutatus* (Paol.) Thell.	Wild	Italy. Tuscany (Botanic Garden).	Europe	*D. carota* subsp. *gummifer*
PI 478883	*D. carota* subsp. *commutatus*	Wild	France. Finistere: maritime turf, Le Conquet.	Europe	*D. carota* subsp. *gummifer*
Ames 26381	*D. carota* subsp. *fontanesii* Thell.	Wild	Portugal. Faro: Near Portimao.	Iberian Peninsula	*D. carota* subsp. *gummifer*
Ames 31193	*D. carota* subsp. *gadecaei* (Rouy & E. G. Camus) Heywood	Wild	France.	Europe	*D. carota* subsp. *gummifer*
Ames 26382	*D. carota* subsp. *gummifer* (Syme) Hook. f.	Wild	Portugal. Faro: Near Sagres.	Iberian Peninsula	
Ames 26383	*D. carota* subsp. *gummifer*	Wild	Portugal. Faro: Near Aljezur.	Iberian Peninsula	
Ames 26384	*D. carota* subsp. *gummifer*	Wild	Portugal. Beja: Near Cavaleiro	Iberian Peninsula	
PI 652411	*D. carota* subsp. *gummifer*	Wild	France. Finistere: Pointe de Rospico, Navez.	Europe	
Ames 25898	*D. guttatus* Sm. (20) +	Wild	Turkey. Konya: Konya, toward Beysehir.		
PI 286611	*D. guttatus* +	Wild	Source: Lebanon. American University of Beirut: Faculty of Agricultural Sciences.		
PI 652387	*D. guttatus* +	Wild	Turkey. Antalya.		

Ames 31194	*D. carota* subsp. *halophilus* (Brot.) A. Pujadas	Wild	Unknown	Iberian Peninsula	*D. carota* subsp. *gummifer*
Ames 24682	*D. carota* subsp. *major* (Vis.) Arcang.	Wild	Portugal. Coimbra: Souselas.	Iberian Peninsula	*D. carota* subsp. *carota*
Ames 25017	*D. carota* subsp. *major*	Wild	Germany. Saxony-Anhalt.	Europe	*D. carota* subsp. *carota*
PI 652229	*D. carota* subsp. *major*	Wild	Tunisia. Aryanah (Institut National de la Recherche Agronomique de Tunisie).	North Africa	*D. carota* subsp. *carota*
Ames 26391	*D. carota* subsp. *maritimus* (Lam.) Batt.	Wild	Portugal. Coimbra: Near Buarcos.	Iberian Peninsula	*D. carota* subsp. *carota*
Ames 26392	*D. carota* subsp. *maritimus*	Wild	Portugal. Leiria: Near Batalha.	Iberian Peninsula	*D. carota* subsp. *carota*
Ames 26393	*D. carota* subsp. *maritimus*	Wild	Portugal. Castelo Branco: Near Perdiagao	Iberian Peninsula	*D. carota* subsp. *carota*
Ames 26394	*D. carota* subsp. *maritimus*	Wild	Portugal. Portalegre near Monforte.	Iberian Peninsula	*D. carota* subsp. *maximus*
Ames 26390	*D. carota* subsp. *maritimus*	Wild	Portugal. Coimbra: Near Mesura.	Iberian Peninsula	*D. carota* subsp. *carota*
PI 502244	*D. carota* subsp. *maritimus*	Wild	Portugal. Coimbra: Lousa.	Iberian Peninsula	*D. carota* subsp. *carota*
PI 652225	*D. carota* subsp. *maritimus*	Landrace	France. Paris (Museum National d'Histoire Naturelle).		*D. carota* subsp. *sativus*
Ames 26402	*D. carota* subsp. *maximus* (Desf.) Ball	Wild	Portugal. Portalegre: Near Sao Vicente e Ventosa.	Iberian Peninsula	
Ames 26407	*D. carota* subsp. *maximus*	Wild	Portugal. Faro: Near Olhao.	Iberian Peninsula	
Ames 26408	*D. carota* subsp. *maximus*	Wild	Portugal. Beja: Near Monte Velho.	Iberian Peninsula	
Ames 26401	*D. carota* subsp. *maximus*	Wild	Portugal. Portalegre: Near Monforte.	Iberian Peninsula	
PI 652230	*D. carota* subsp. *maximus*	Wild	Albania. Lushnjë: Along road going to field plots of Wheat Institute.	Balkan Peninsula	*D. carota* subsp. *carota*
PI 279777	*D. carota* subsp. *sativus* (Hoffm.) Schubl. & G.	Landrace	Egypt. Giza (Orman Botanic Garden.).		

	Martens				
PI 432900	*D. carota* subsp. *sativus*	Cultivated	China.	Eastern	
PI 432901	*D. carota* subsp. *sativus*	Cultivated	China.	Eastern	
PI 451752	*D. carota* subsp. *sativus*	Cultivated	Netherlands.	Western	
PI 509434	*D. carota* subsp. *sativus*	Cultivated	Turkey. Sivas.	Eastern	
PI 652152	*D. carota* subsp. *sativus*	Cultivated	United Kingdom.	Western	
PI 652155	*D. carota* subsp. *sativus*	Cultivated	Hungary. Pest.	Western	
PI 652414	*D. crinitus* Desf. (22) +	Wild	Portugal. Faro: Near Bengado.		
PI 652355	*D. involucratus* Sm. (22) +	Wild	Turkey. Izmir: 5 km north of Kusadasi.		
PI 295857	*D. littoralis* Sibth. & Sm. (20) +	Wild	Israel. Beit Alpha.		
PI 661242	*D. pusillus* Michx. (22) +	Wild	USA. Oregon: near Hunters River Cove, Curry.		
Ames 29096	*D. sahariensis* Murb. (18)	Wild	Tunisia.		*D. syrticus*
Ames 29097	*D. sahariensis*	Wild	Tunisia.		*D. syrticus*
Ames 29098	*D. sahariensis*	Wild	Tunisia.		*D. syrticus*
PI 652329	*D. setulosus* Guss. (not reported) +	Wild	Greece. Peloponnese: 4 km from Skoura, toward Leonidion, Laconia Prefecture.		
Ames 29108	*D. syrticus* Murb. (18)	Wild	Tunisia.		
Ames 29109	*D. syrticus*	Wild	Tunisia.		
Ames 29110	*D. syrticus*	Wild	Tunisia.		
PI 649477	*Orlaya daucoides* (L.) Greuter (20) +	Wild	Turkey. Aydin: Dilek Peninsula Reserve.		
PI 674284	*Rouya polygama* (Desf.) Coincy (20) +	Wild	Tunisia. Jendouba: Road to Tabarka, near Tabarka airport.		

[a] Plant Introduction (PI) numbers are permanent numbers designated to germplasm accessions maintained at the

National Plant Germplasm System (NPGS) in Ames, Iowa, USA. Ames numbers are assigned for carrots and other Apiaceae in the NPGS temporarily to newly acquired germplasm until passport data of an accession and taxonomy is verified. Also, accessions with Ames numbers have to be determined they are not duplicate accessions, and that they can be successfully maintained by the USDA. It is not guaranteed these accessions may be assigned a PI number after the assessment period.

[b] These names correspond to those in the Germplasm Resources Information Network (GRIN) database (https://npgsweb.ars-grin.gov/gringlobal/search.aspx?). The 14 accessions designated with a plus sign were used to demonstrate the utility of GBS data to obtain the dominant topology of *Daucus* (Arbizu et al. [36]).

[c] Location refers to where the germplasm was collected in the wild. If the accession was obtained through another entity (vendor, genebank, botanic garden), the source is indicated within parenthesis.
