# Supplementary material for: Genotyping-by-sequencing provides the discriminating power to investigate the subspecies of Daucus carota (Apiaceae)
Source: BMC Evol Biol. 2016 Oct 28;16:234. doi: 10.1186/s12862-016-0806-x (PMC5084430; doi:10.1186/s12862-016-0806-x)
Supplement: Additional file 2: Table S2. — Summary of processing GBS data of Daucus. (PDF 98 kb) [file 12862_2016_806_MOESM2_ESM.pdf]

Additional file 2: Table S2. Summary of processing GBS data.

Number of accessions[a]	Unique Sequence Tags[b]	Raw Marker Sites[c]	Filter 1[d]	Filter 2[e]	Filter 3A[f]	Filter 3B[f]
164	16,291,308	889,445	735,501	114,148	38,920	10,814
150	14,548,150	789,311	719,918	111,215	43,713	18,565

[a]Number of accessions used in the present study. One hundred sixty four accessions were used to determine the outgroup for the *D. carota* complex. On the other hand, 150 accessions were employed to determine the classification of the *D. carota* complex.

[b]GBS tags are DNA sequences. Number of reads having a barcode and a restriction site.

[c]Number of SNPs obtained after executing the discovery SNP caller.

[d]Number of SNPs after executing a filtering option that merges duplicate SNPs occurring in overlapping tags from opposite strands. A threshold genotypic mismatch of 0.1 was also considered as a filtering option.

[e]Number of SNPs obtained after removing SNPs not in statistically LD with at least one neighboring marker. A minimum R-square value for LD filter was 0.1.

[f]Filtering options for SNPs were: (i) minimum minor allele frequency of 0.1, (ii) maximum minor allele frequency of one, (iii) number of alleles less than or equal to two, and (iv) maximum missing data of 0.3 (filter 3A) and 0.1 (filter 3B).
