# Supplementary figures and images for: Genotyping-by-sequencing provides the discriminating power to investigate the subspecies of Daucus carota (Apiaceae)
Source: BMC Evol Biol. 2016 Oct 28;16:234. doi: 10.1186/s12862-016-0806-x (PMC5084430; doi:10.1186/s12862-016-0806-x)

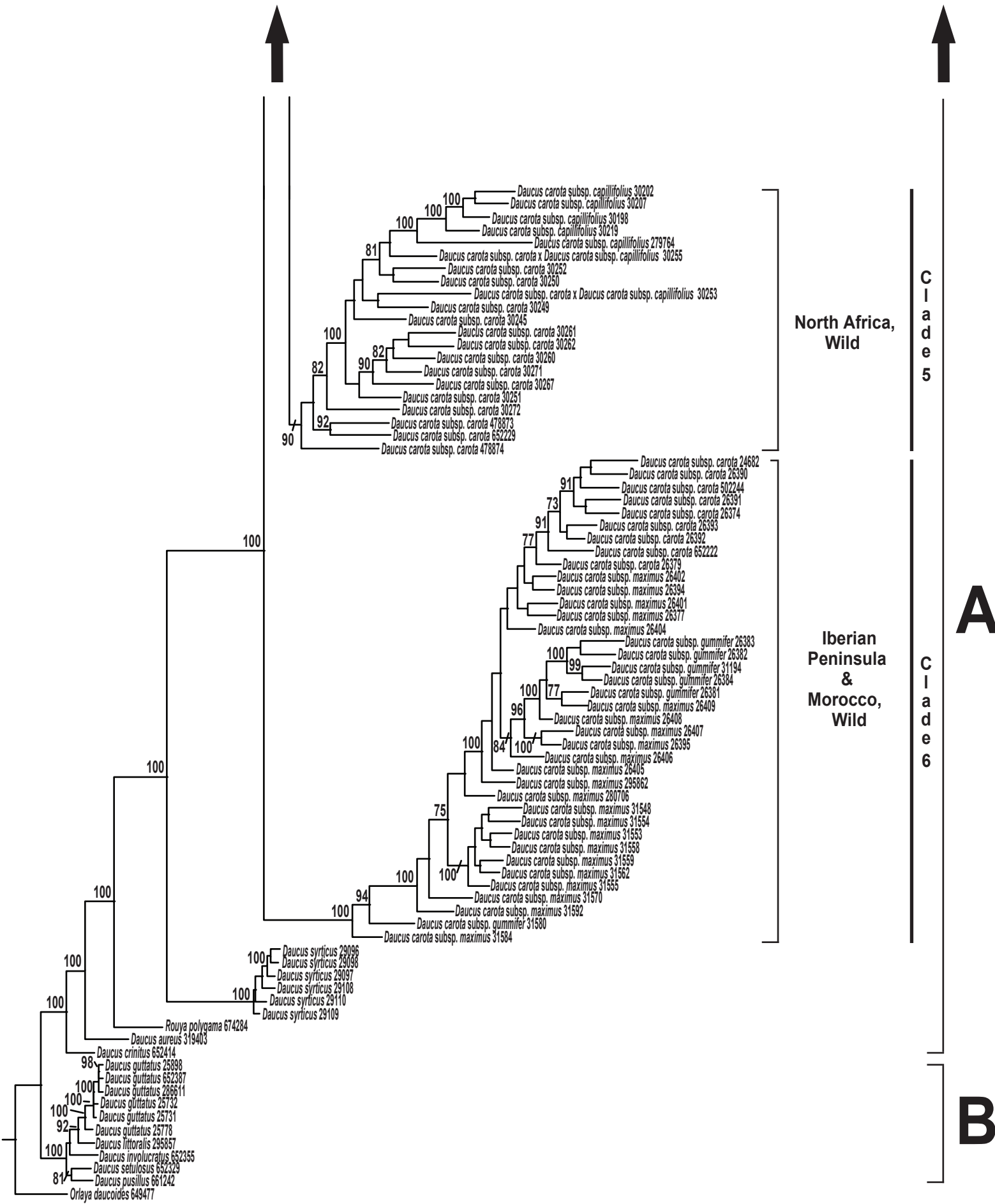

Supplement: Additional file 3: Figure S1. — Phylogenomics of Daucus from a maximum likelihood analysis using 164 accessions and 10,814 SNPs (10% missing imputed genotypes) obtained by GBS. Numbers above branches represent bootstrap values, with only values higher than 70% shown. Names given to clades refer to the geographic origin and improvement status of the accessions of the D. carota complex. Clades A and B corresponds to the two main groups of the Daucus phylogeny. (PDF 1.39 mb) [file 12862_2016_806_MOESM3_ESM.pdf]

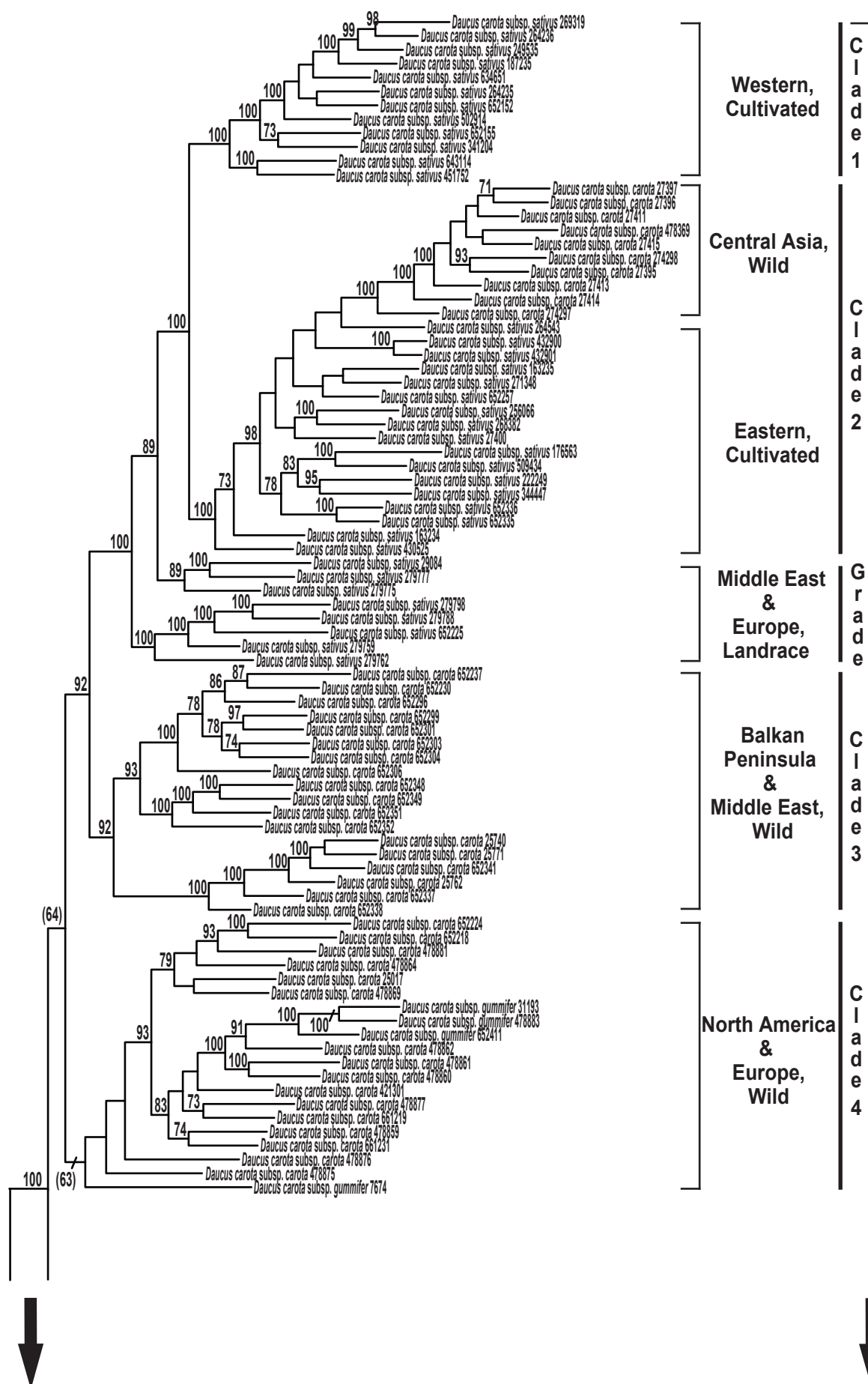

# A

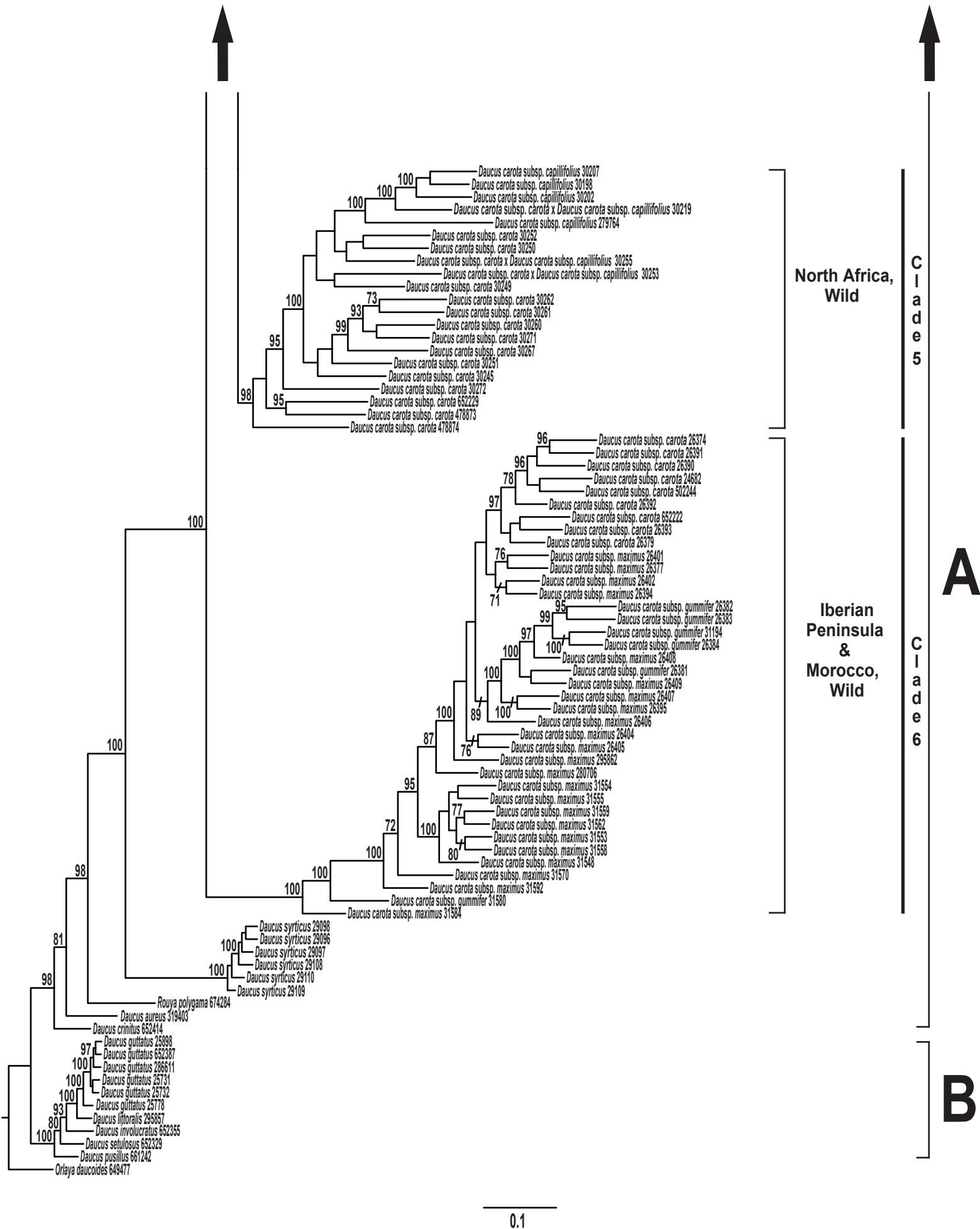

0.1

Supplement: Additional file 4: Figure S2. — Phylogenomics of Daucus from a maximum likelihood analysis using 164 accessions and 38,920 SNPs (30% missing imputed genotypes) obtained by GBS. Numbers above branches represent bootstrap values, with only values higher than 70% shown. Names given to clades refer to the geographic origin and improvement status of the accessions of the D. carota complex. Clades A and B corresponds to the two main groups of the Daucus phylogeny. (PDF 1.34 mb) [file 12862_2016_806_MOESM4_ESM.pdf]

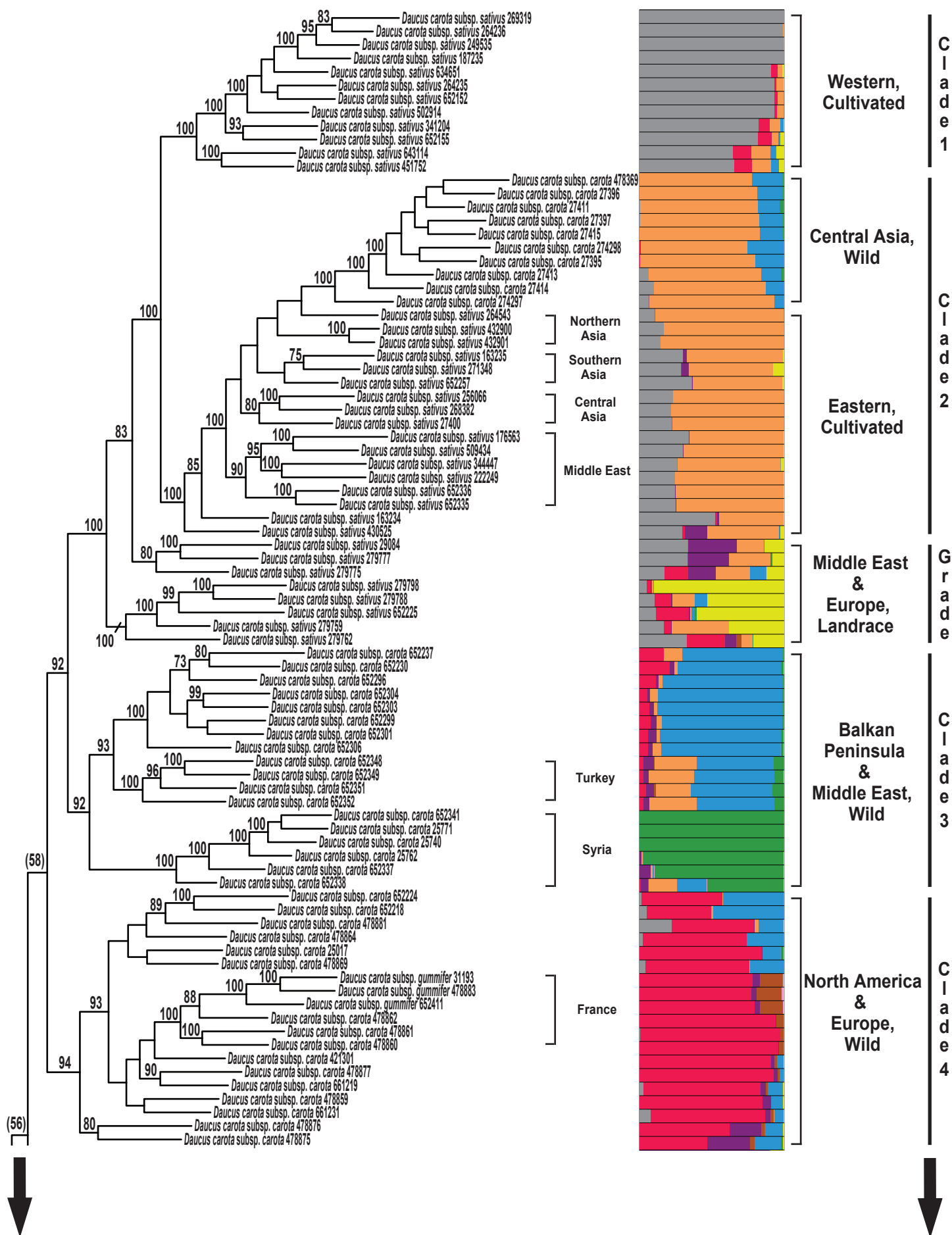

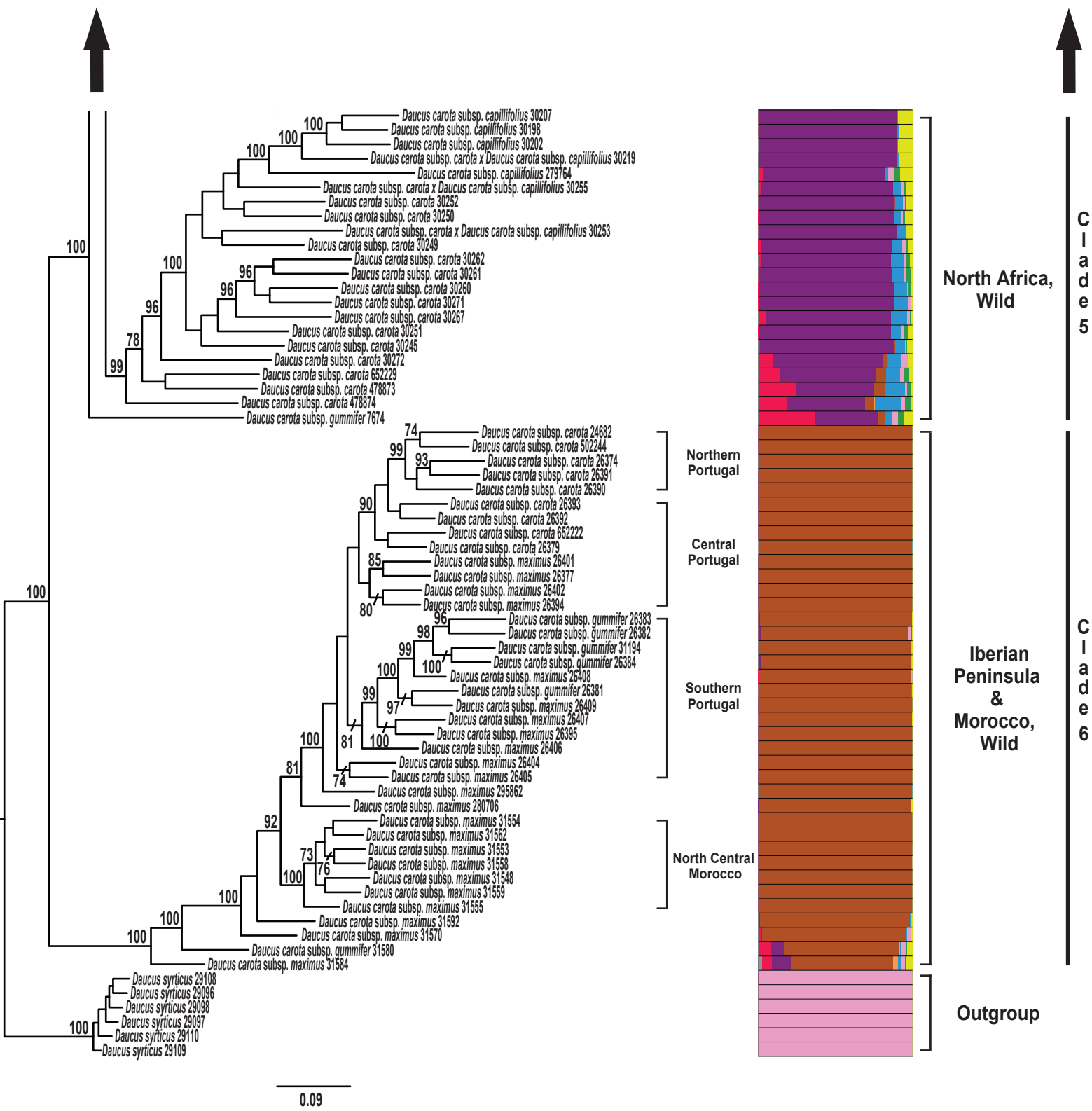

Supplement: Additional file 5: Figure S3. — Maximum likelihood reconstruction and structure of the genetic diversity of 144 accessions of the Daucus carota complex and outgroups using 43,713 SNPs (30% missing imputed genotypes) obtained by GBS. Each accession is represented by a horizontal bar, and each color corresponds to a population (nine in total). Numbers above branches represent bootstrap values, with only values higher than 70% shown. Names given to clades refer to the geographic origin and improvement status of the accessions of the D. carota complex. The outgroup taxon is D. syrticus. (PDF 1.46 mb) [file 12862_2016_806_MOESM5_ESM.pdf]

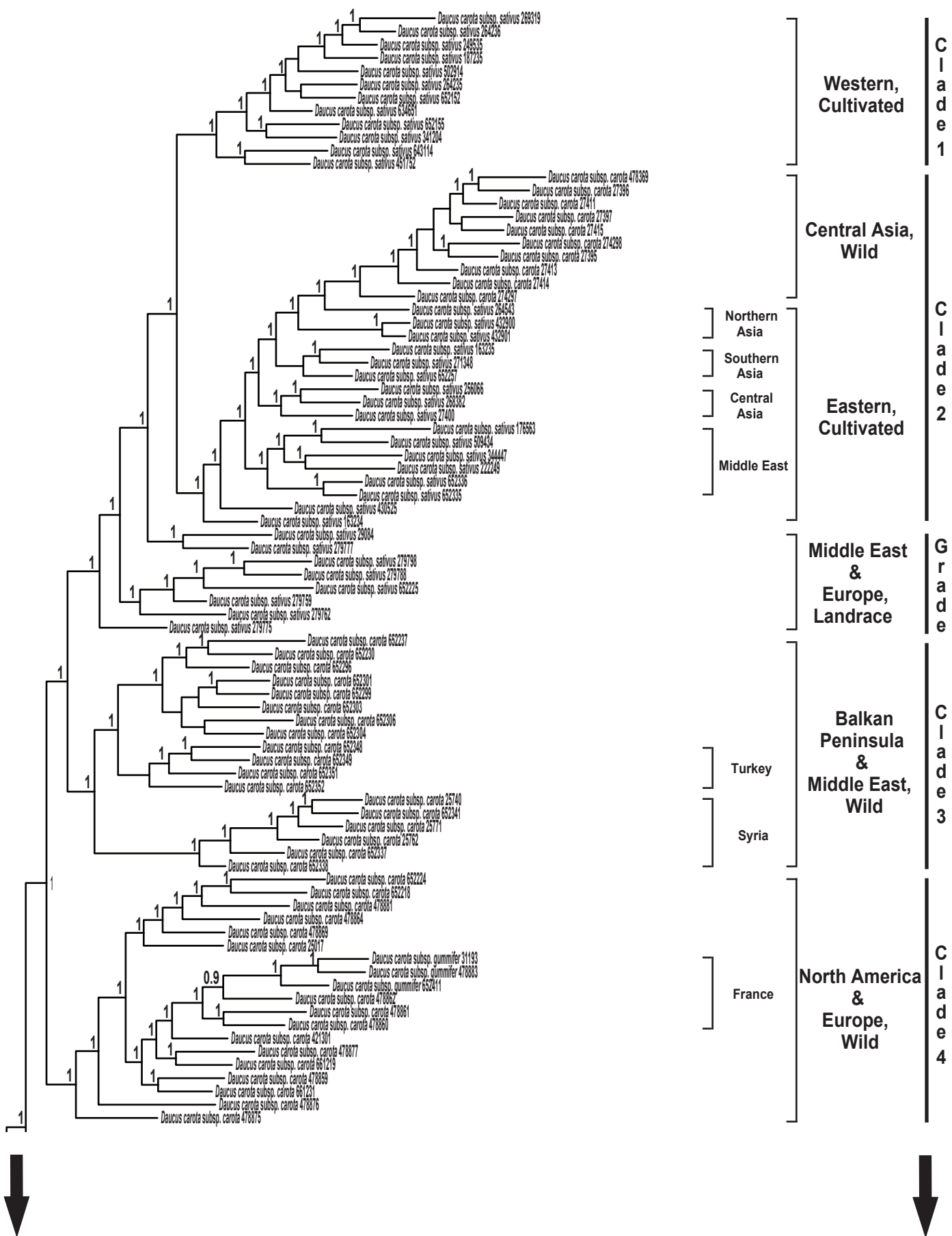

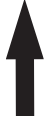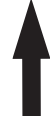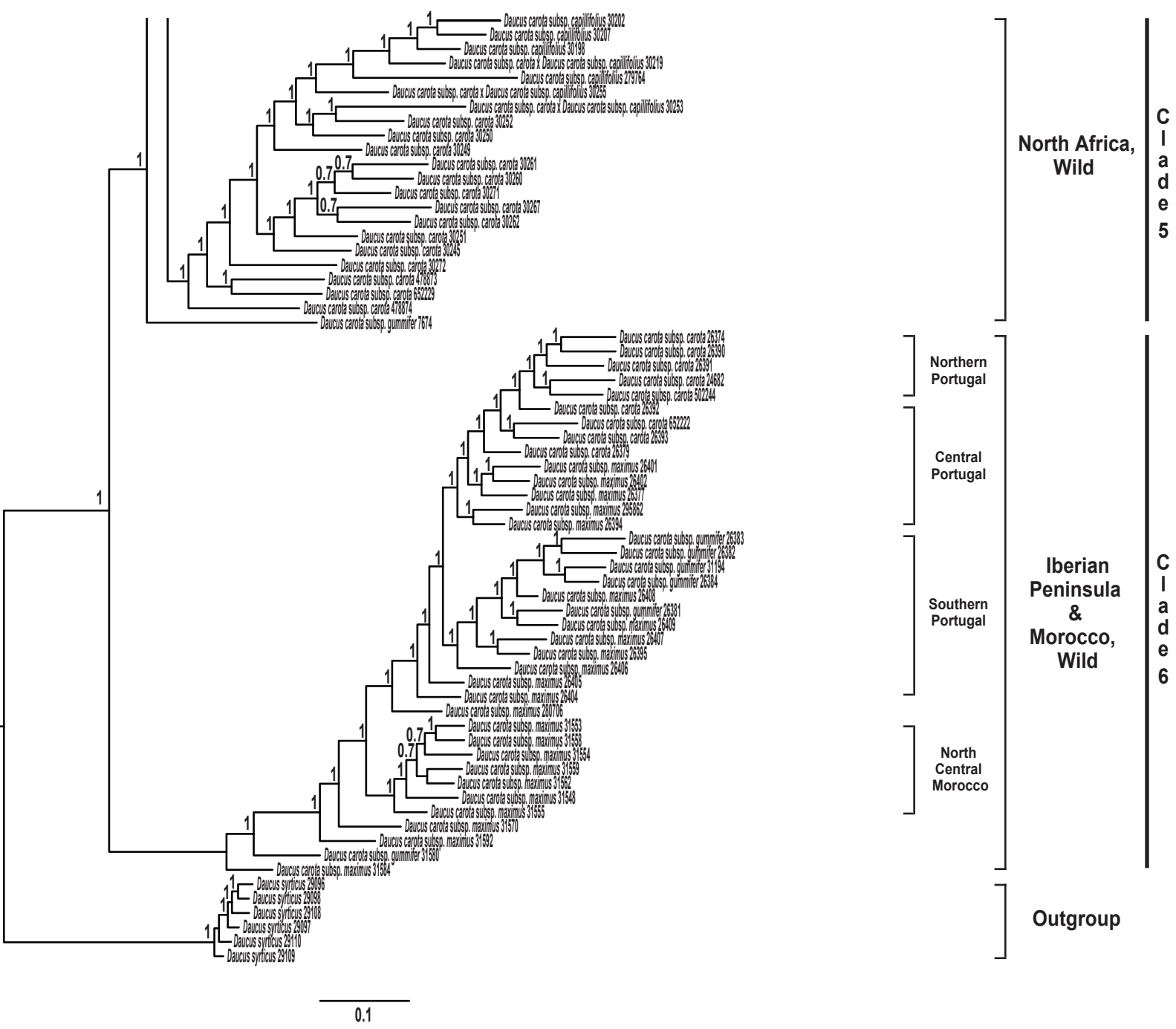

Supplement: Additional file 6: Figure S4. — Bayesian phylogenetic tree of 144 accessions of the Daucus carota complex and outgroups using 18,565 SNPs (10% missing imputed genotypes) obtained by GBS. Numbers above the branches represent posterior probabilities, with only values higher than 0.7 shown. Names given to clades refer to the geographic origin and improvement status of the accessions of the D. carota complex. The outgroup taxon is D. syrticus. (PDF 1.33 mb) [file 12862_2016_806_MOESM6_ESM.pdf]

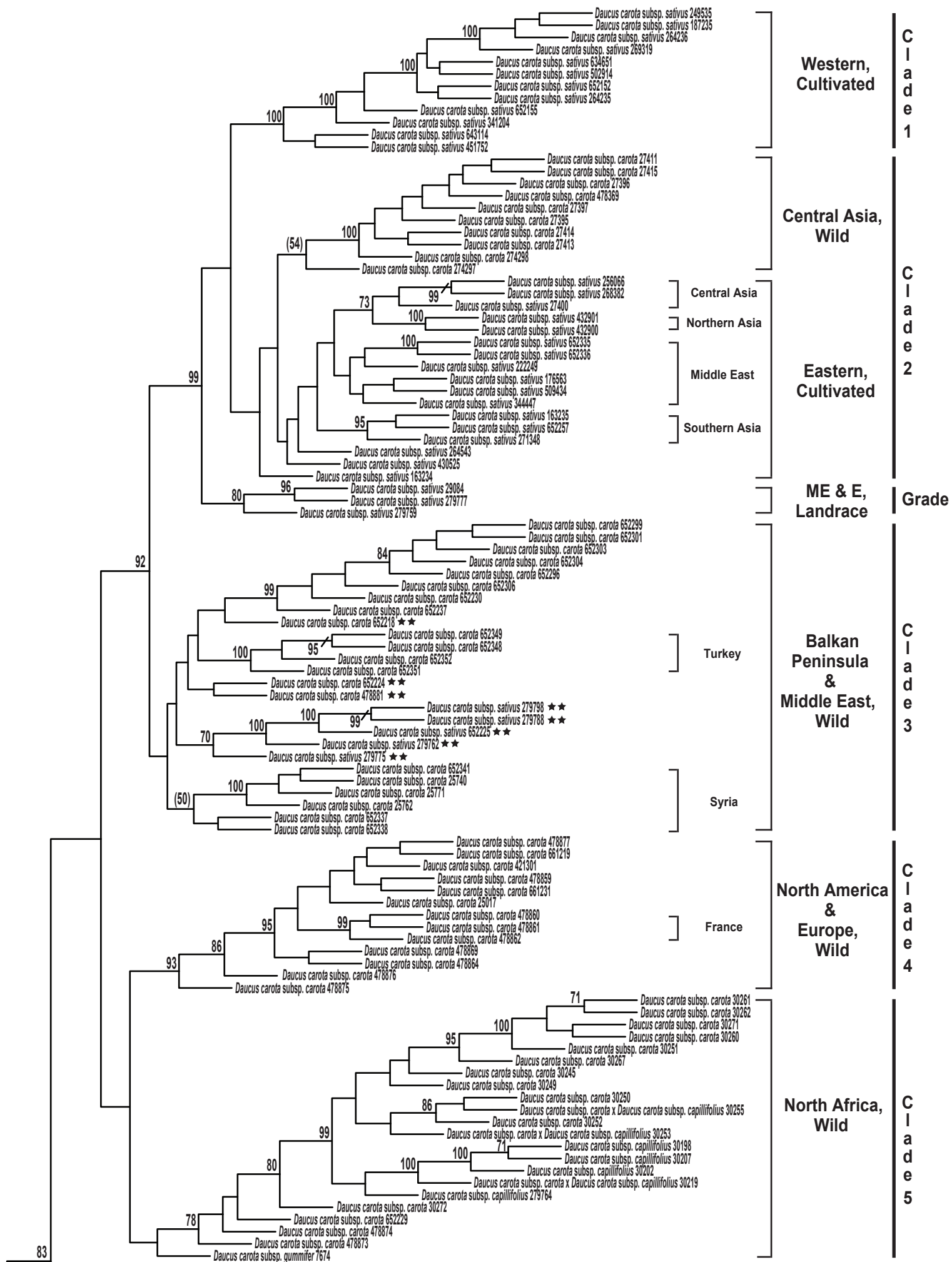

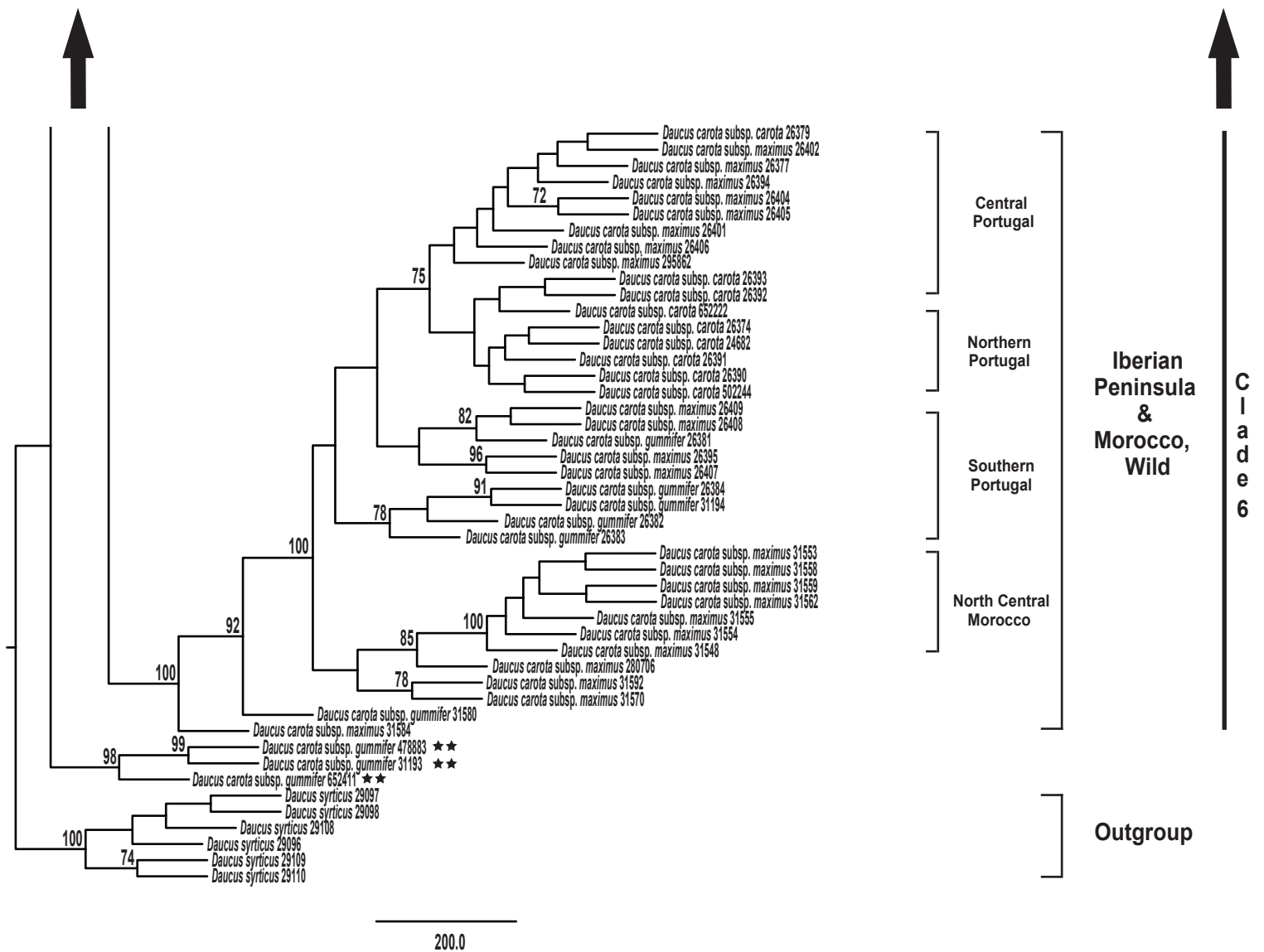

Supplement: Additional file 7: Figure S5. — Relationships among 144 accessions of the Daucus carota complex and outgroups from an exhaustive quartet sampling inference using 18,565 SNPs (10% missing imputed genotypes) obtained by GBS. Numbers above the branches represent bootstrap values, with only values higher than 70% shown. Names given to clades refer to the geographic origin and improvement status of the accessions of the D. carota complex. ME & E refers to Middle East & Europe. Accessions designated by double stars are misplaced relative to the maximum likelihood topology of the Daucus carota complex using the same number of SNPs. The outgroup taxon is D. syrticus. (PDF 1.30 mb) [file 12862_2016_806_MOESM7_ESM.pdf]

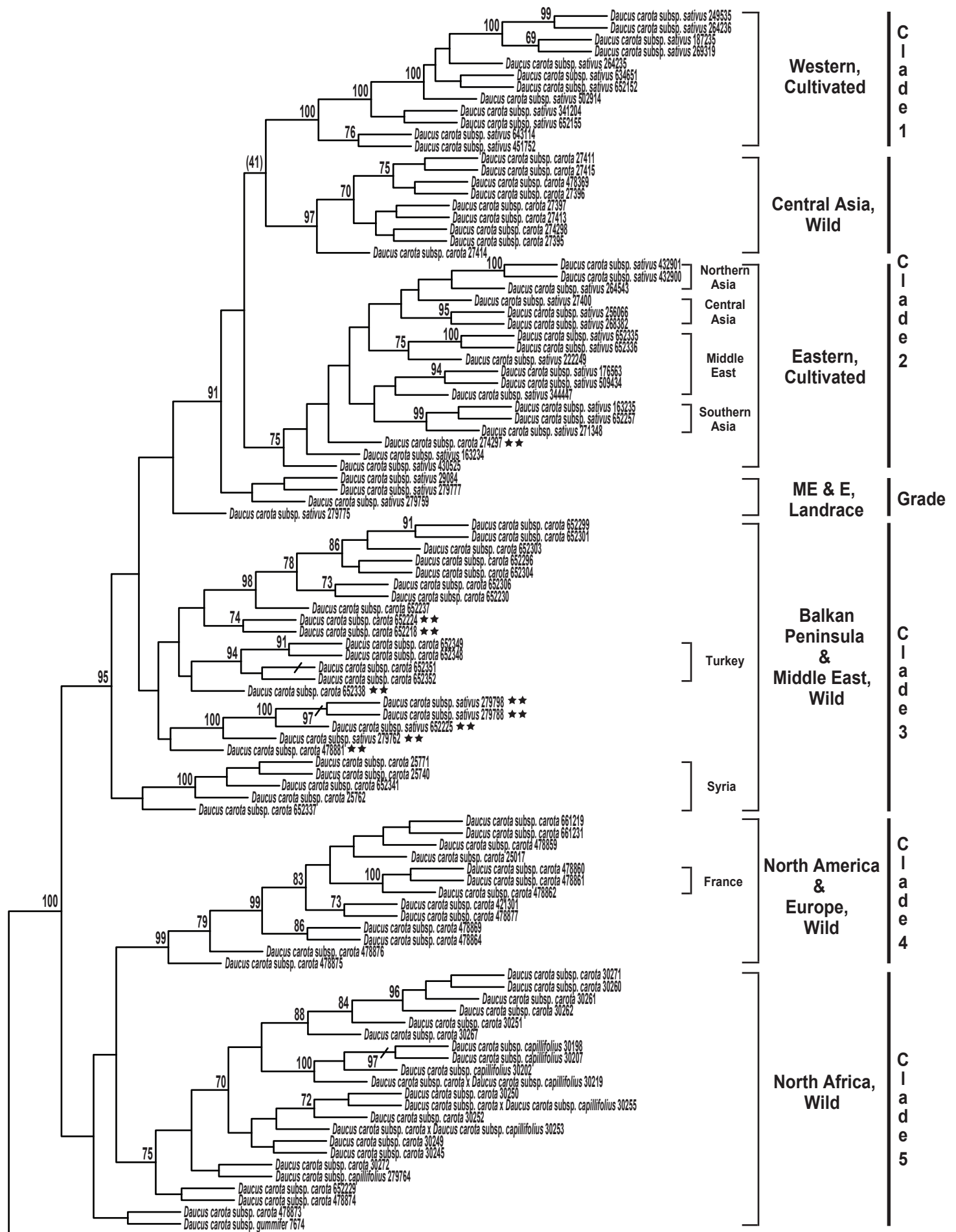

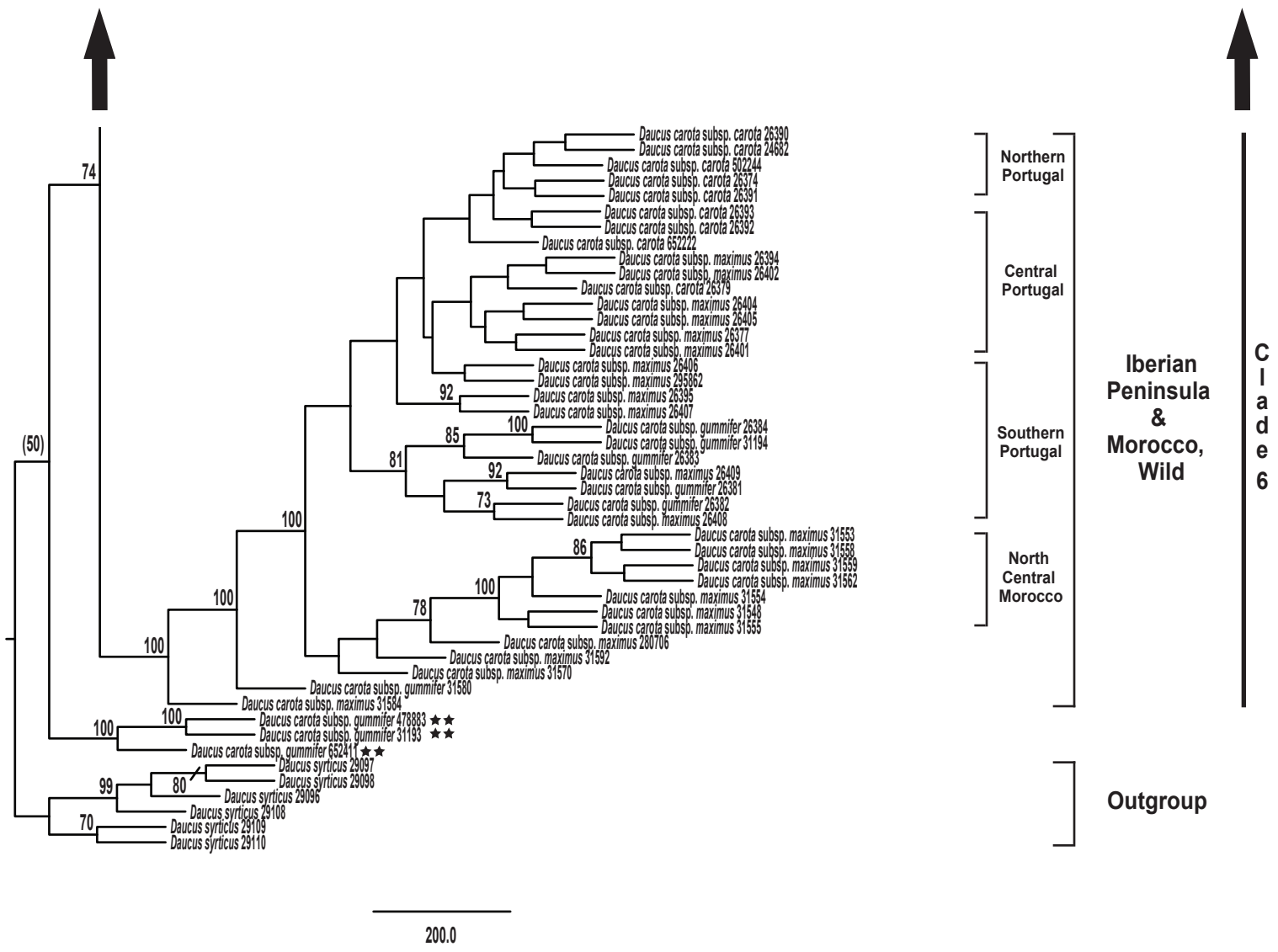

Supplement: Additional file 8: Figure S6. — Relationships among 144 accessions of the Daucus carota complex and outgroups from an exhaustive quartet sampling inference using 43,713 SNPs (30% missing imputed genotypes) obtained by GBS. Numbers above branches represent bootstrap values, with only values higher than 70% shown. Names given to clades refer to the geographic origin and improvement status of the accessions of the D. carota complex. ME & E refers to Middle East & Europe. Accessions designated by double stars are misplaced relative to the maximum likelihood topology of the Daucus carota complex using the same number of SNPs. The outgroup taxon is D. syrticus. (PDF 142 kb) [file 12862_2016_806_MOESM8_ESM.pdf]

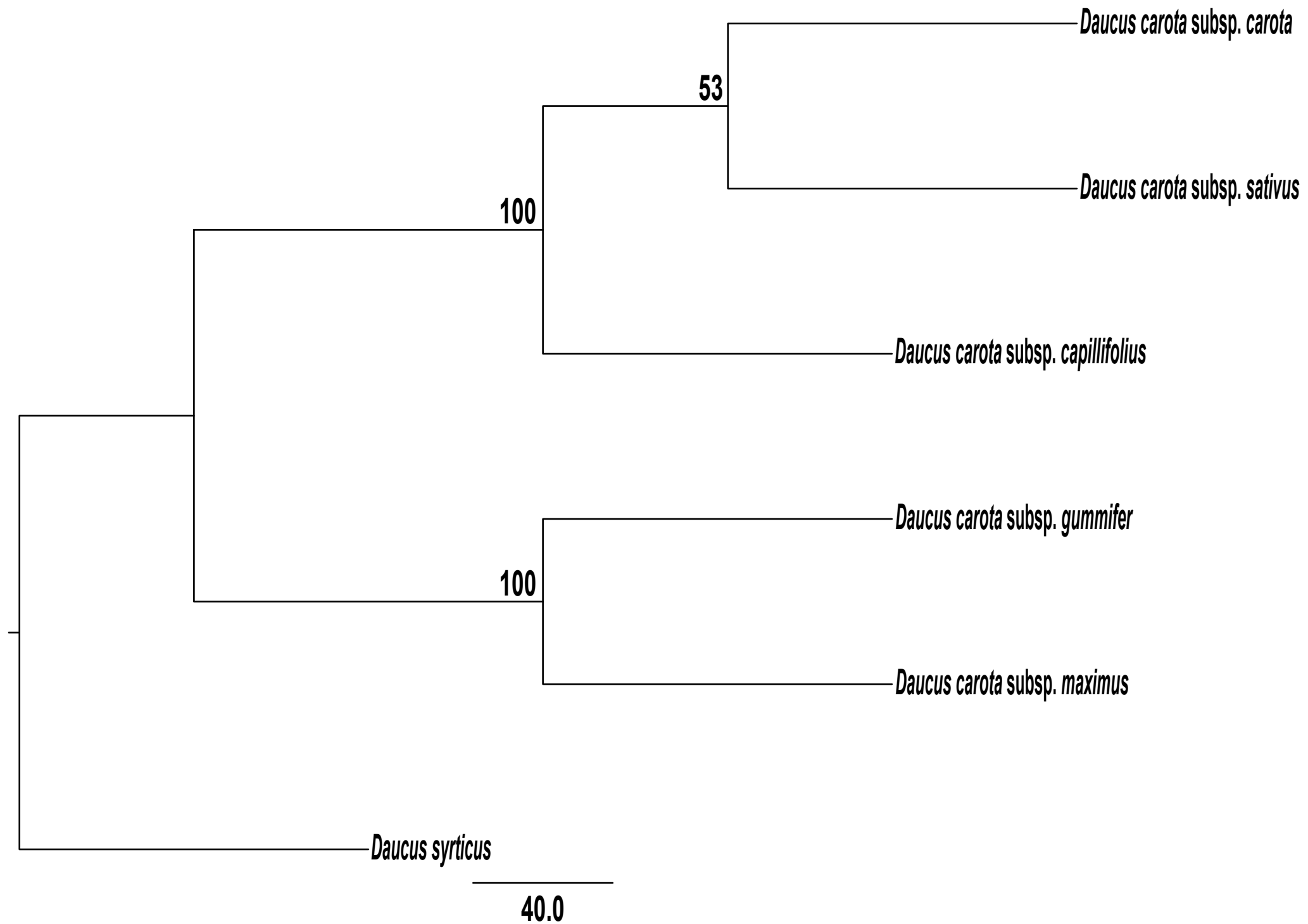

Supplement: Additional file 9: Figure S7. — Species tree of the Daucus carota complex based on a coalescent model using an exhaustive quartet sampling inference and 18,565 SNPs (10% missing imputed genotypes) obtained by GBS. Numbers above the branches represent bootstrap values. The outgroup taxon is D. syrticus. (PDF 104 kb) [file 12862_2016_806_MOESM9_ESM.pdf]

**A**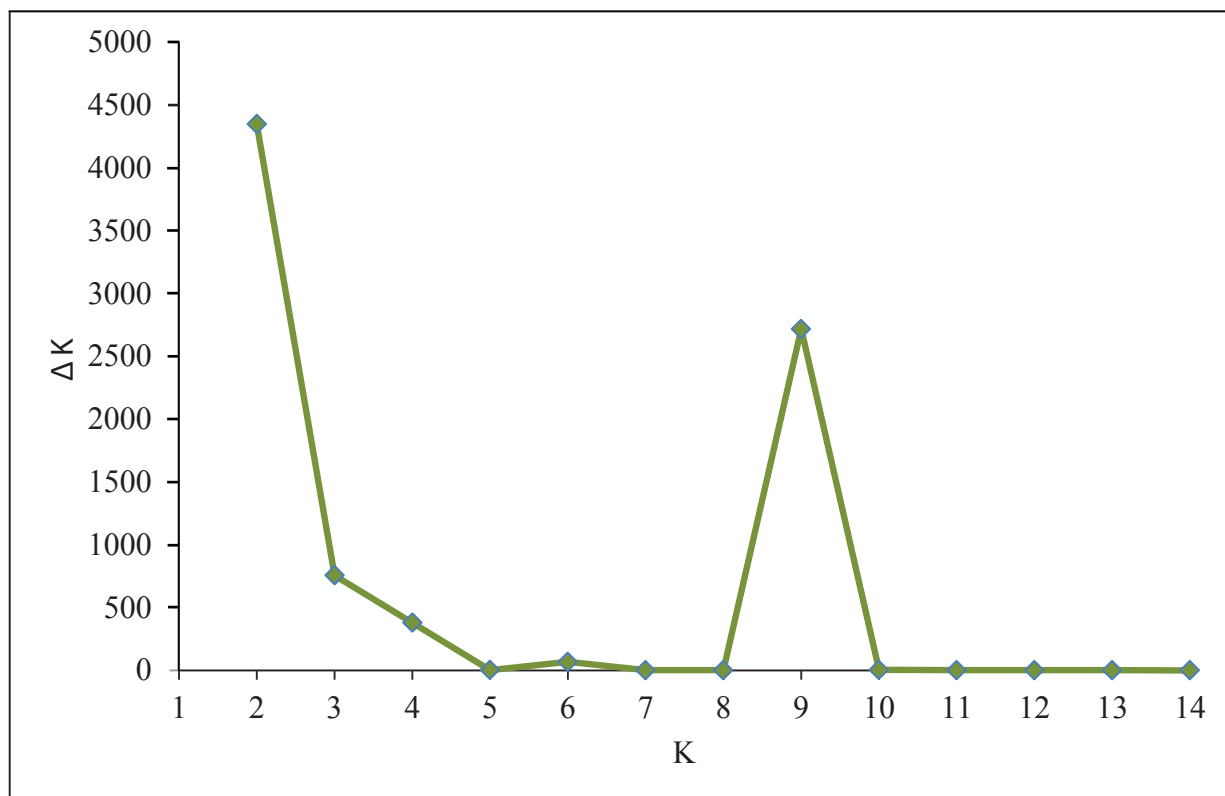**B**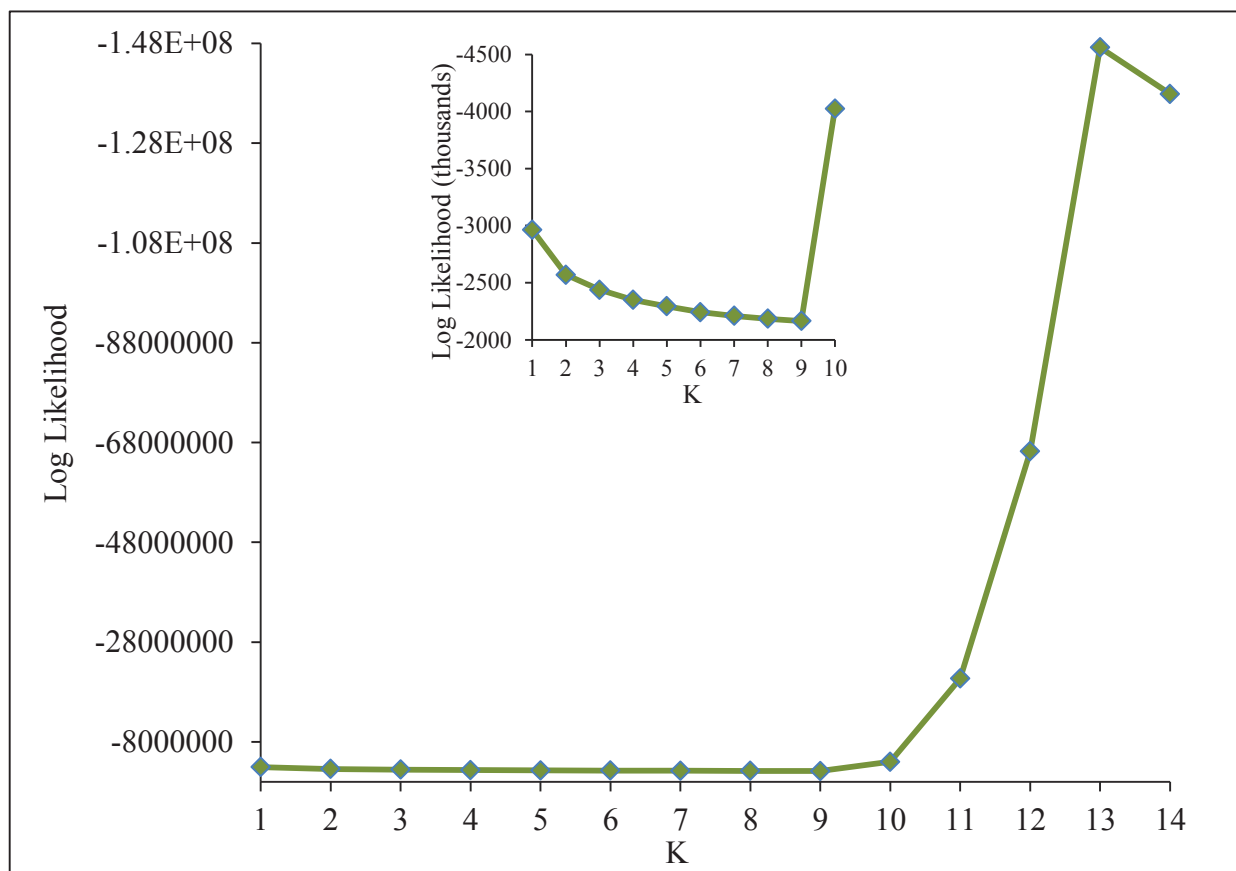

Supplement: Additional file 10: Figure S8. — Number of populations. A. Plot of Delta K (ΔK). B. Plot of the log likelihood; internal plot corresponds to the log likelihood (thousands) for K ranging from 1 to 9. All values were obtained from STRUCTURE HARVESTER analysis. Fourteen populations were considered in a data set of 18,565 SNPs (10% missing imputed genotypes) and 150 samples. (PDF 893 kb) [file 12862_2016_806_MOESM10_ESM.pdf]

**A**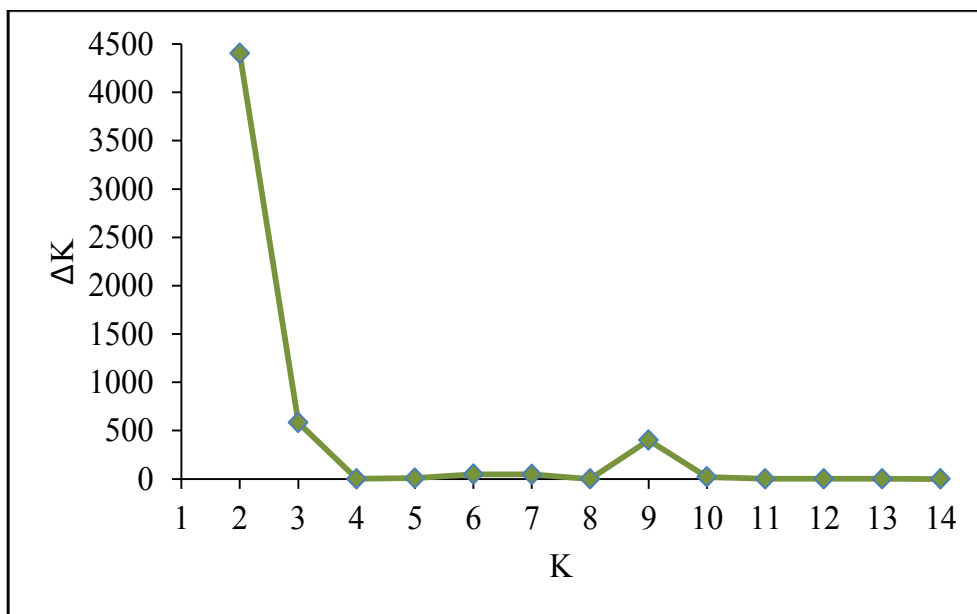**B**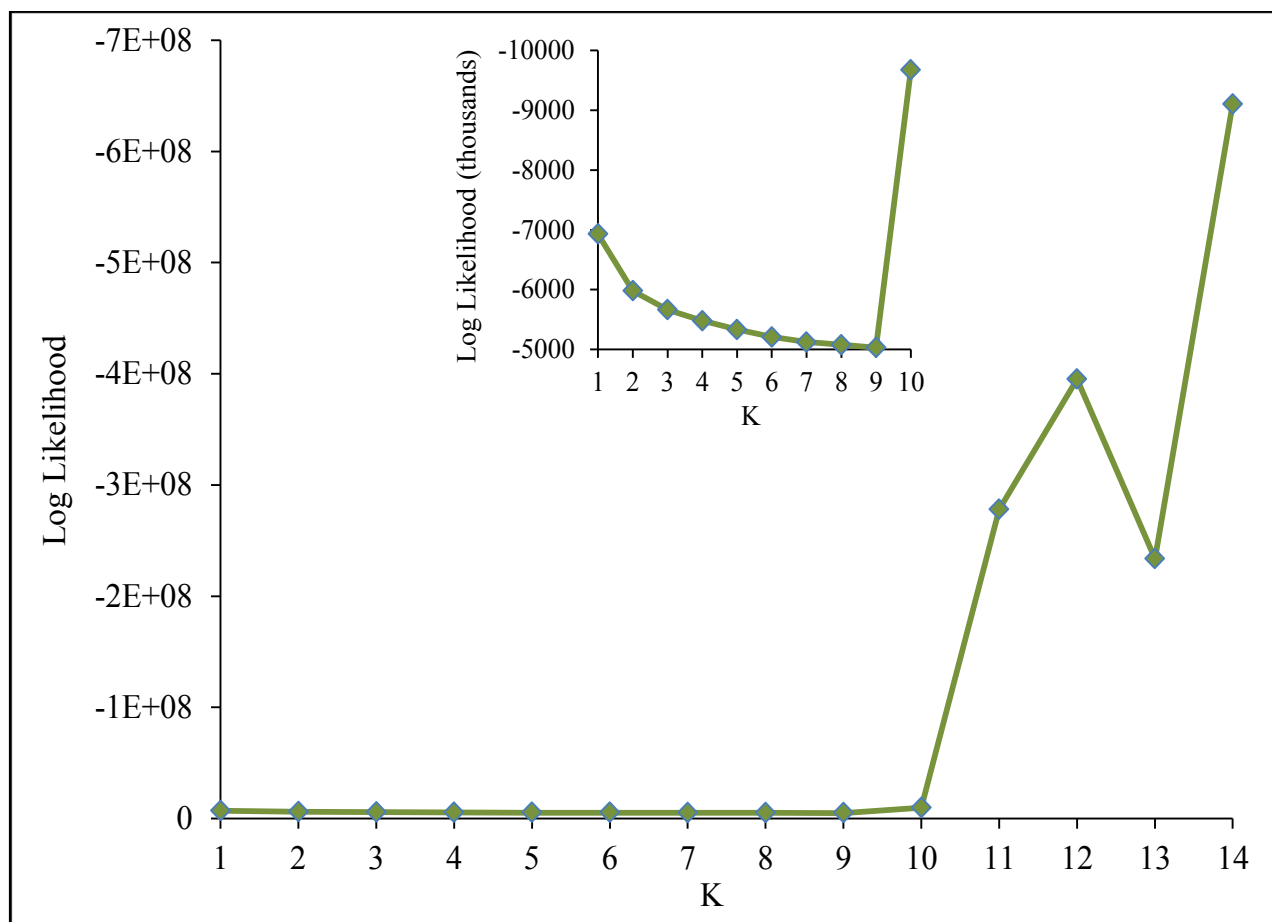

Supplement: Additional file 11: Figure S9. — Number of populations. A. Plot of Delta K (ΔK). B. Plot of the log likelihood; internal plot corresponds to the log likelihood (thousands) for K ranging from 1 to 9. All values were obtained from STRUCTURE HARVESTER analysis. Fourteen populations were considered in a data set of 43,713 SNPs (30% missing imputed genotypes) and 150 samples. (PDF 167 kb) [file 12862_2016_806_MOESM11_ESM.pdf]
