# Supplementary material for: Genotyping-by-sequencing provides the discriminating power to investigate the subspecies of Daucus carota (Apiaceae)
Source: BMC Evol Biol. 2016 Oct 28;16:234. doi: 10.1186/s12862-016-0806-x (PMC5084430; doi:10.1186/s12862-016-0806-x)
Supplement: Additional file 12: Figure S10. — Box plot analyses of the 23 morphological characters examined for members of Daucus carota complex (subsp. sativus not included) in this study. The box plot displays individual plant values for median, 25 and 75% percentile, range, and outliers. (PDF 17.1 mb) [file 12862_2016_806_MOESM12_ESM.pdf]

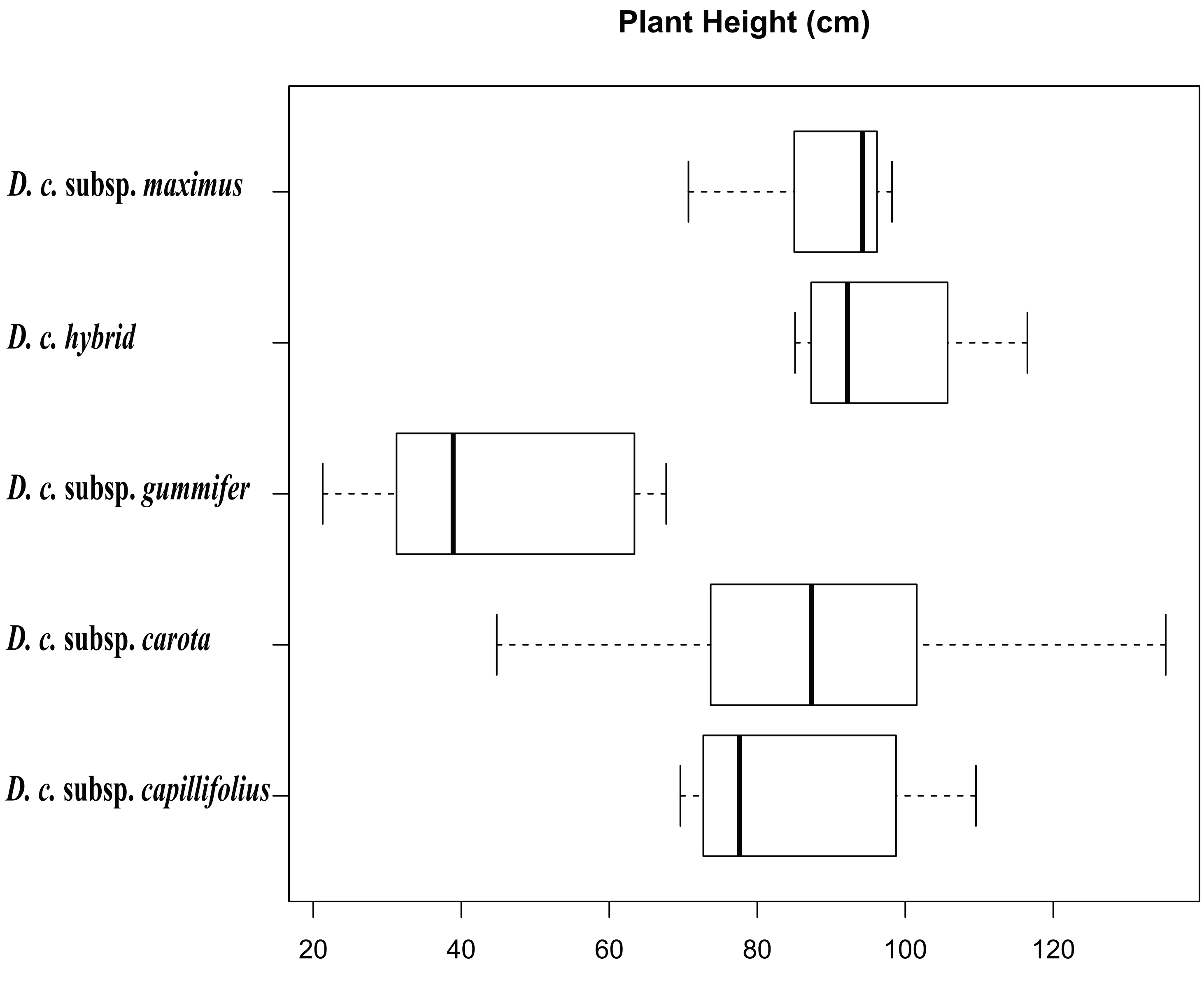

Plant Height (cm)
D. c. subsp. maximus
D. c. hybrid
D. c. subsp. gummifer
D. c. subsp. carota
D. c. subsp. capillifolius
20
40
60
80
100
120

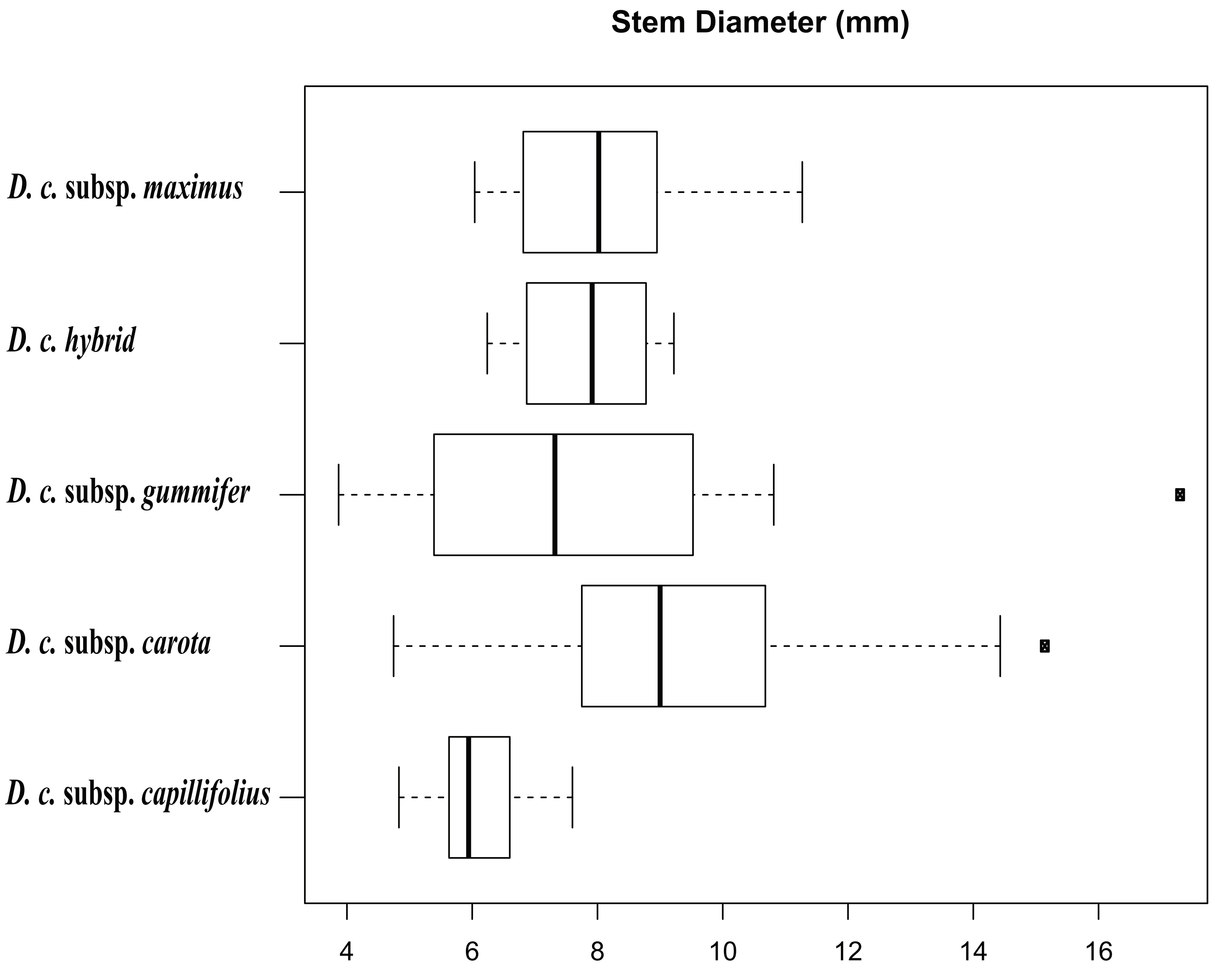
Stem Diameter (mm)
D. c. subsp. maximus
D. c. hybrid
D. c. subsp. gummifer
D. c. subsp. carota
D. c. subsp. capillifolius
4
6
8
10
12
14
16

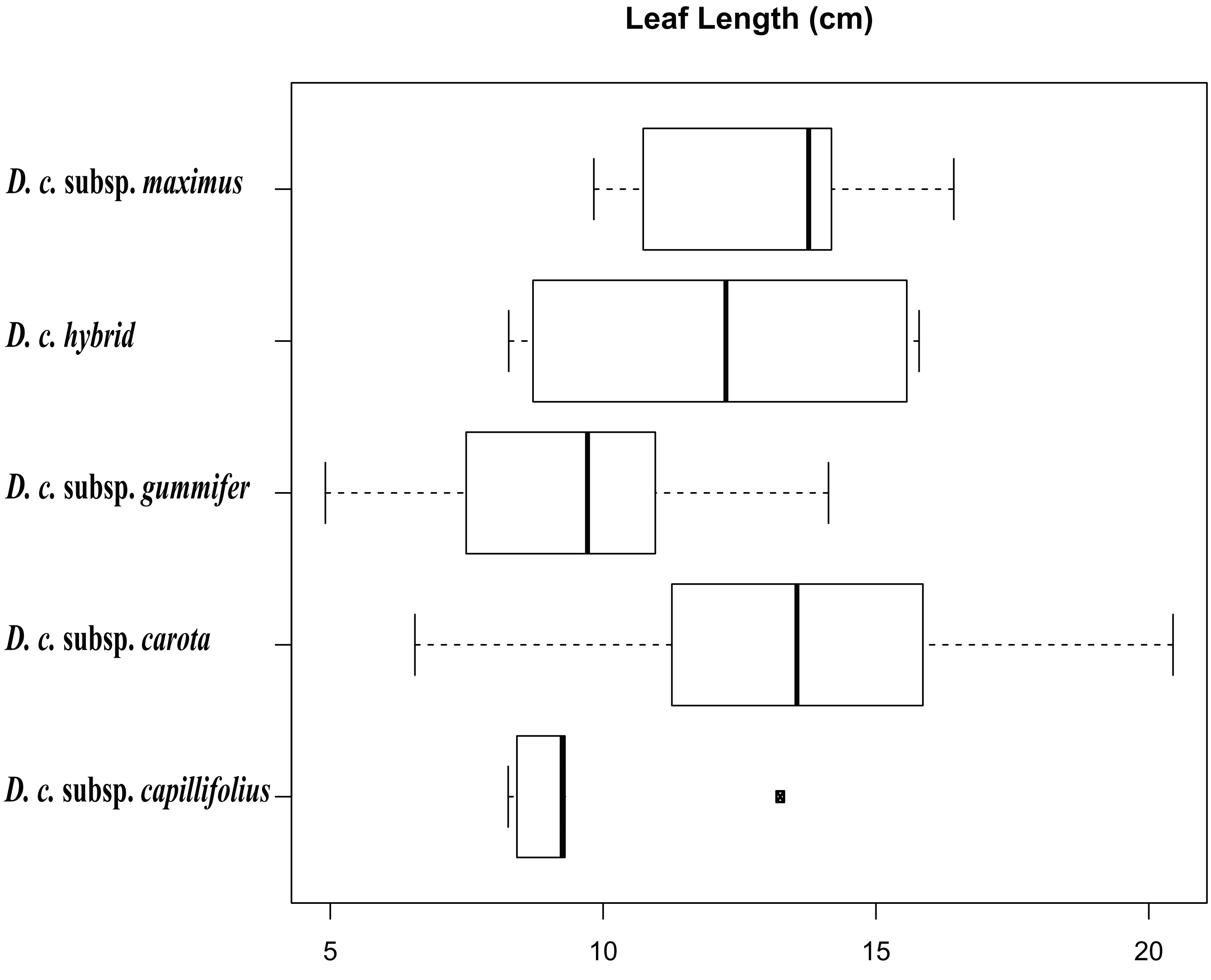

Leaf Length (cm)
D. c. subsp. maximus
D. c. hybrid
D. c. subsp. gummifer
D. c. subsp. carota
D. c. subsp. capillifolius
5
10
15
20

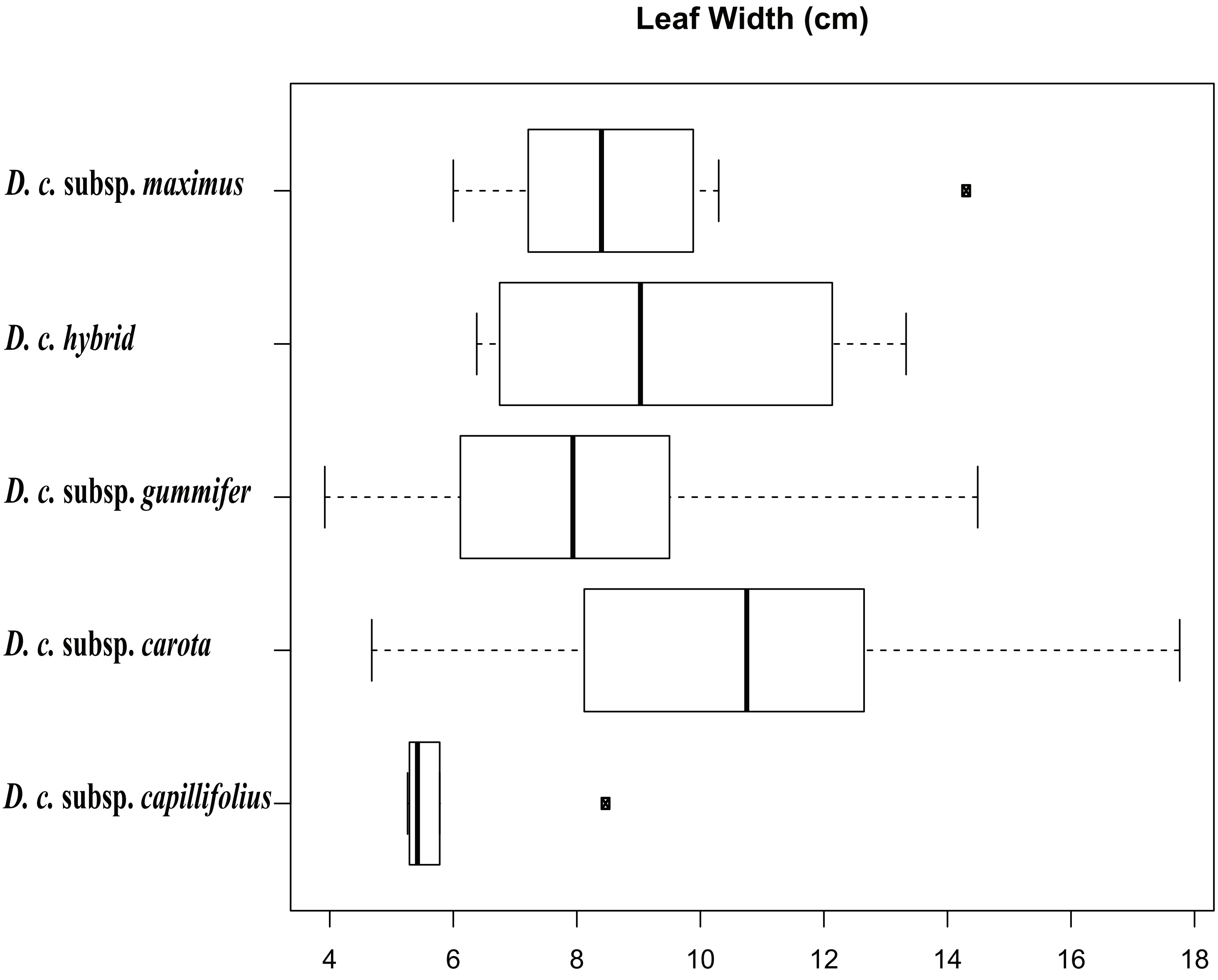

Leaf Width (cm)
D. c. subsp. maximus
D. c. hybrid
D. c. subsp. gummifer
D. c. subsp. carota
D. c. subsp. capillifolius
4
6
8
10
12
14
16
18

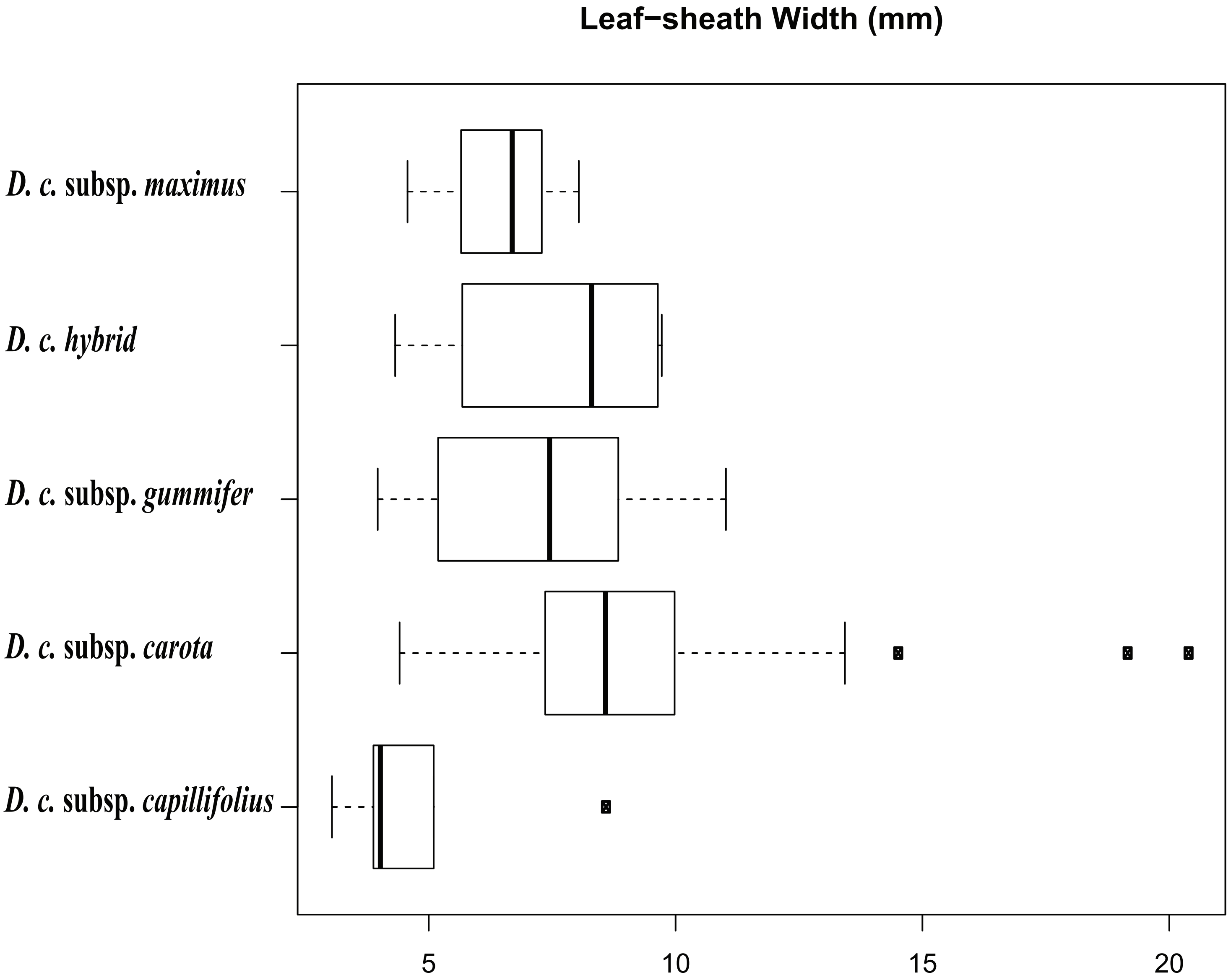

Leaf−sheath Width (mm)
D. c. subsp. maximus
D. c. hybrid
D. c. subsp. gummifer
D. c. subsp. carota
D. c. subsp. capillifolius
5
10
15
20

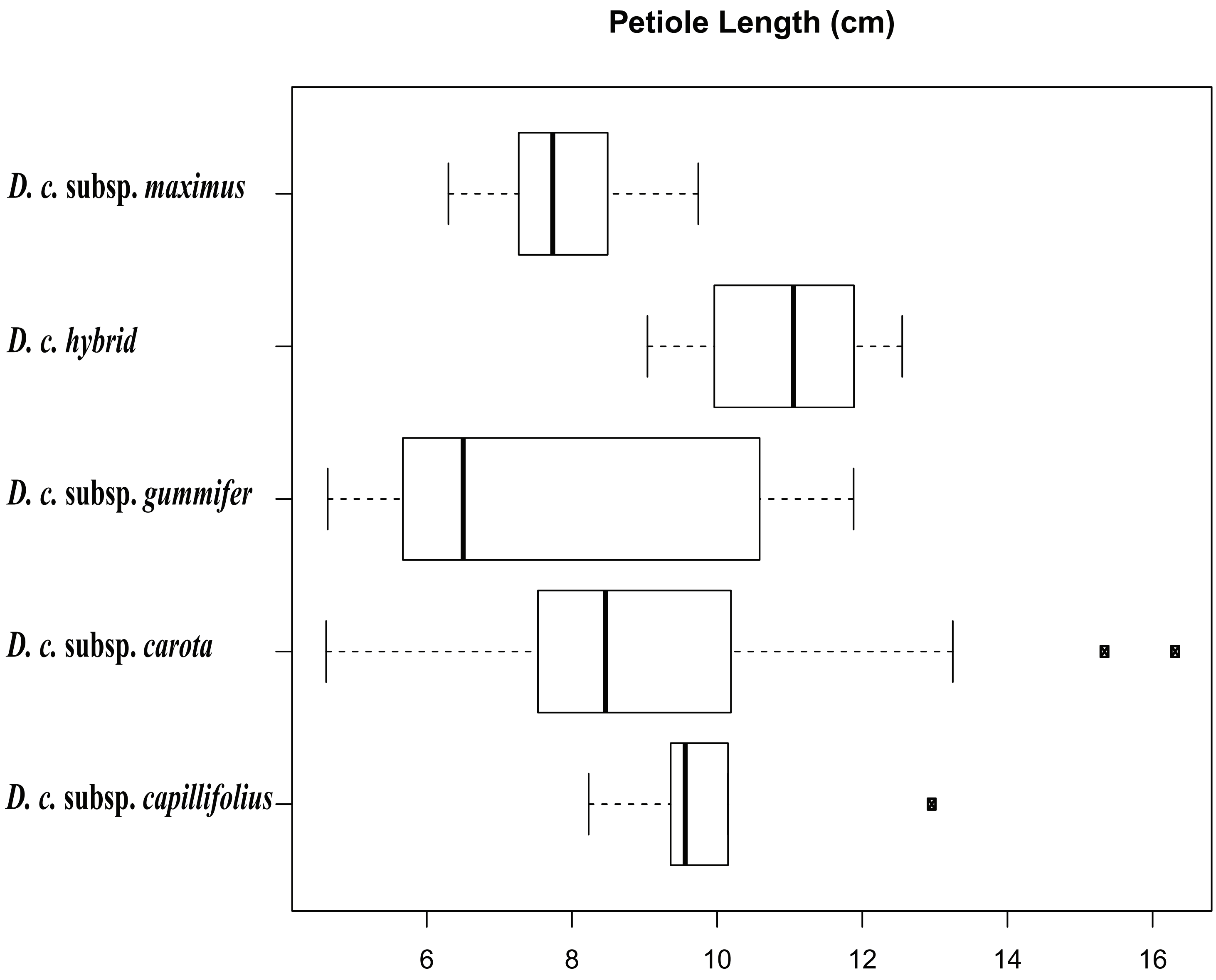
Petiole Length (cm)
D. c. subsp. maximus
D. c. hybrid
D. c. subsp. gummifer
D. c. subsp. carota
D. c. subsp. capillifolius
6
8
10
12
14
16

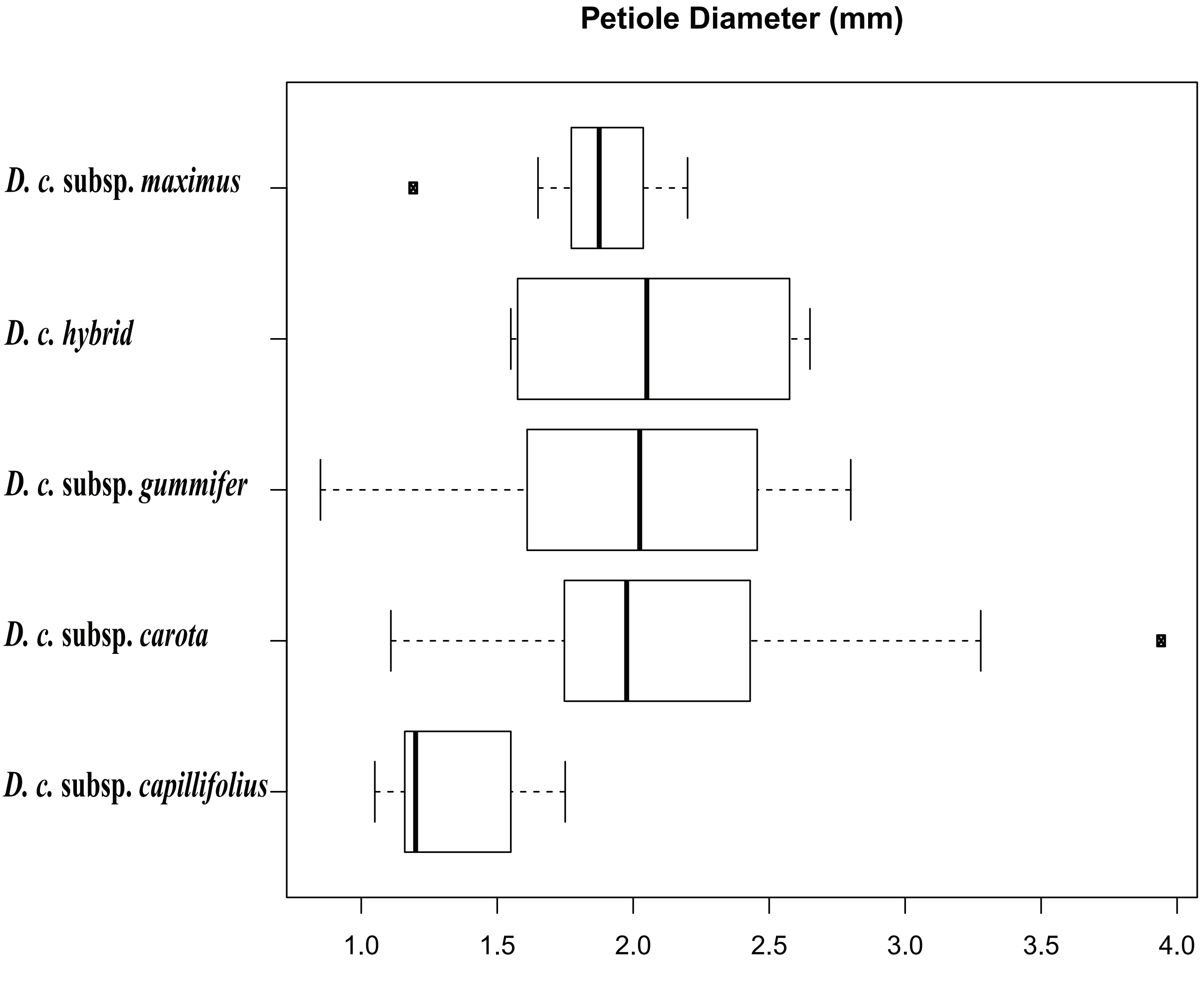

Petiole Diameter (mm)
D. c. subsp. maximus
D. c. hybrid
D. c. subsp. gummifer
D. c. subsp. carota
D. c. subsp. capillifolius
1.0
1.5
2.0
2.5
3.0
3.5
4.0

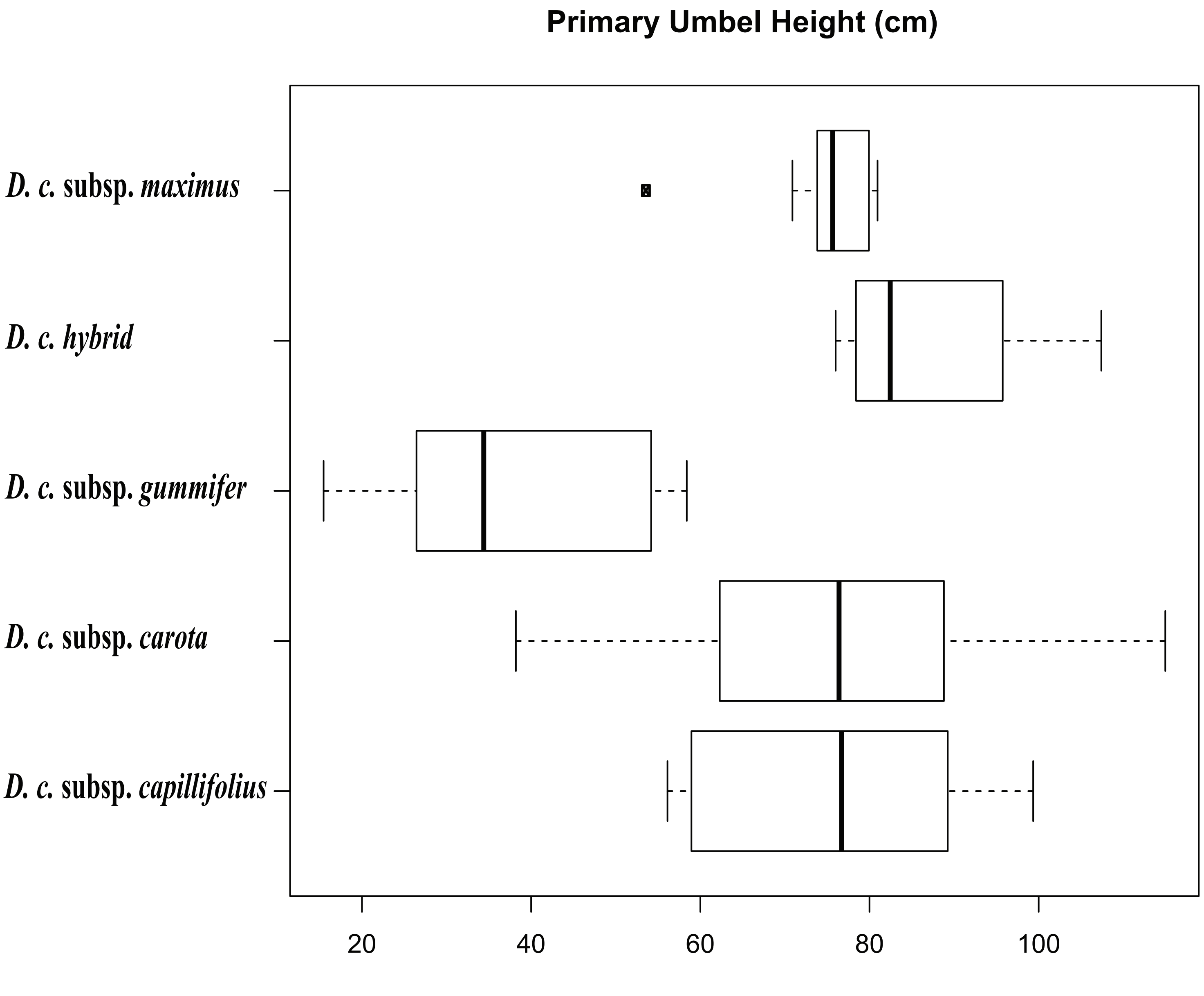
Primary Umbel Height (cm)
D. c. subsp. maximus
D. c. hybrid
D. c. subsp. gummifer
D. c. subsp. carota
D. c. subsp. capillifolius
20
40
60
80
100

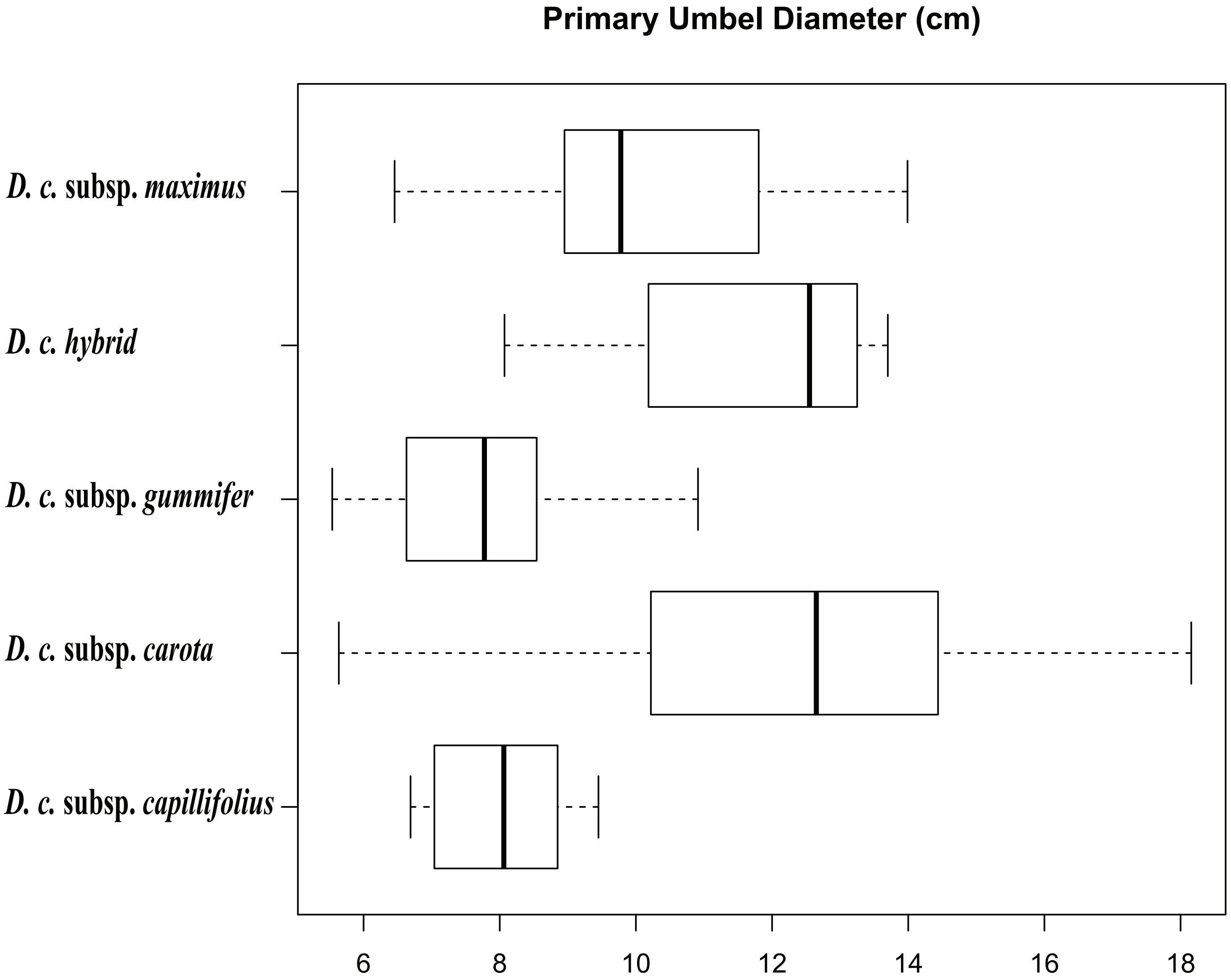
Primary Umbel Diameter (cm)
D. c. subsp. maximus
D. c. hybrid
D. c. subsp. gummifer
D. c. subsp. carota
D. c. subsp. capillifolius
6
8
10
12
14
16
18

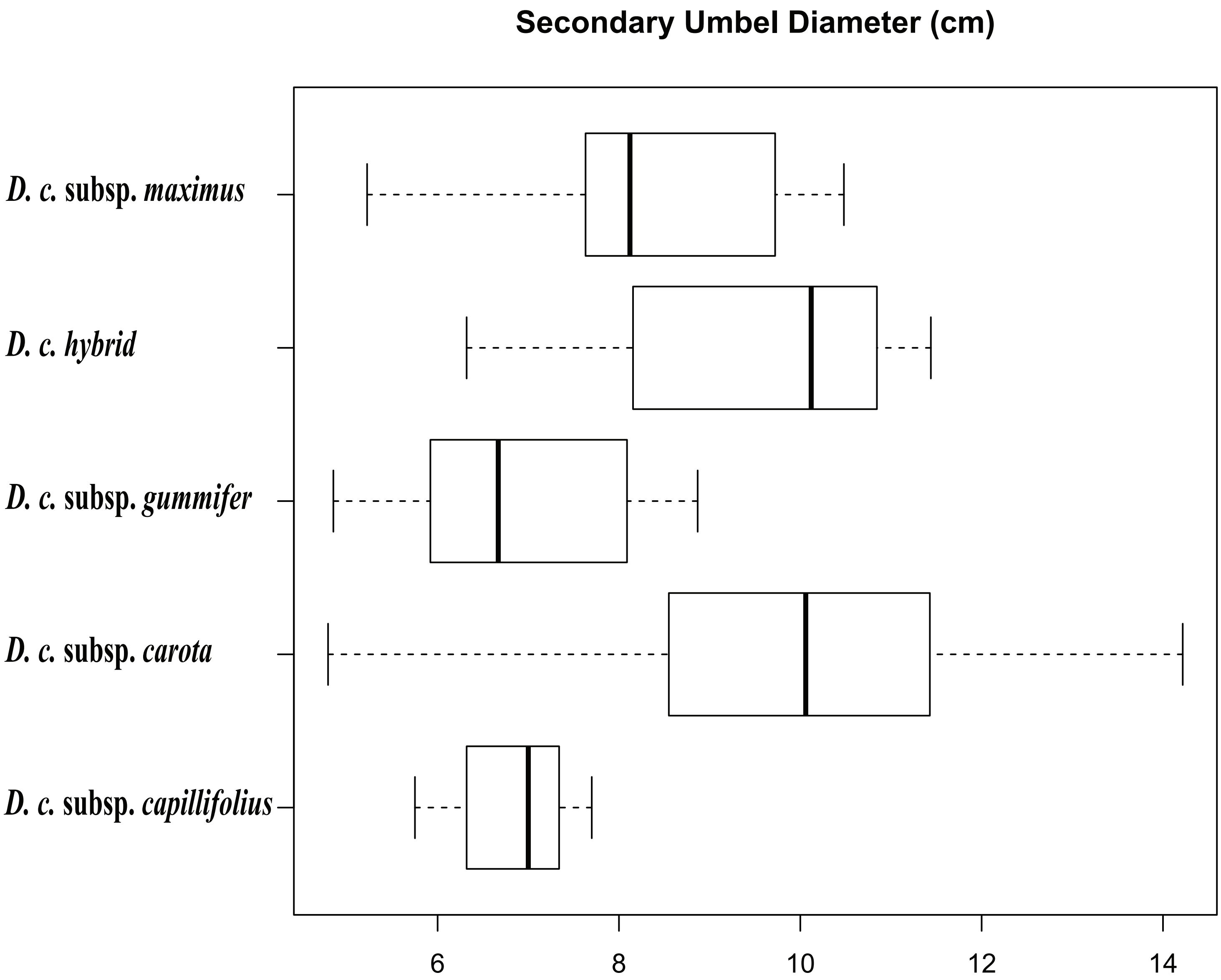

Secondary Umbel Diameter (cm)
D. c. subsp. maximus
D. c. hybrid
D. c. subsp. gummifer
D. c. subsp. carota
D. c. subsp. capillifolius
6
8
10
12
14

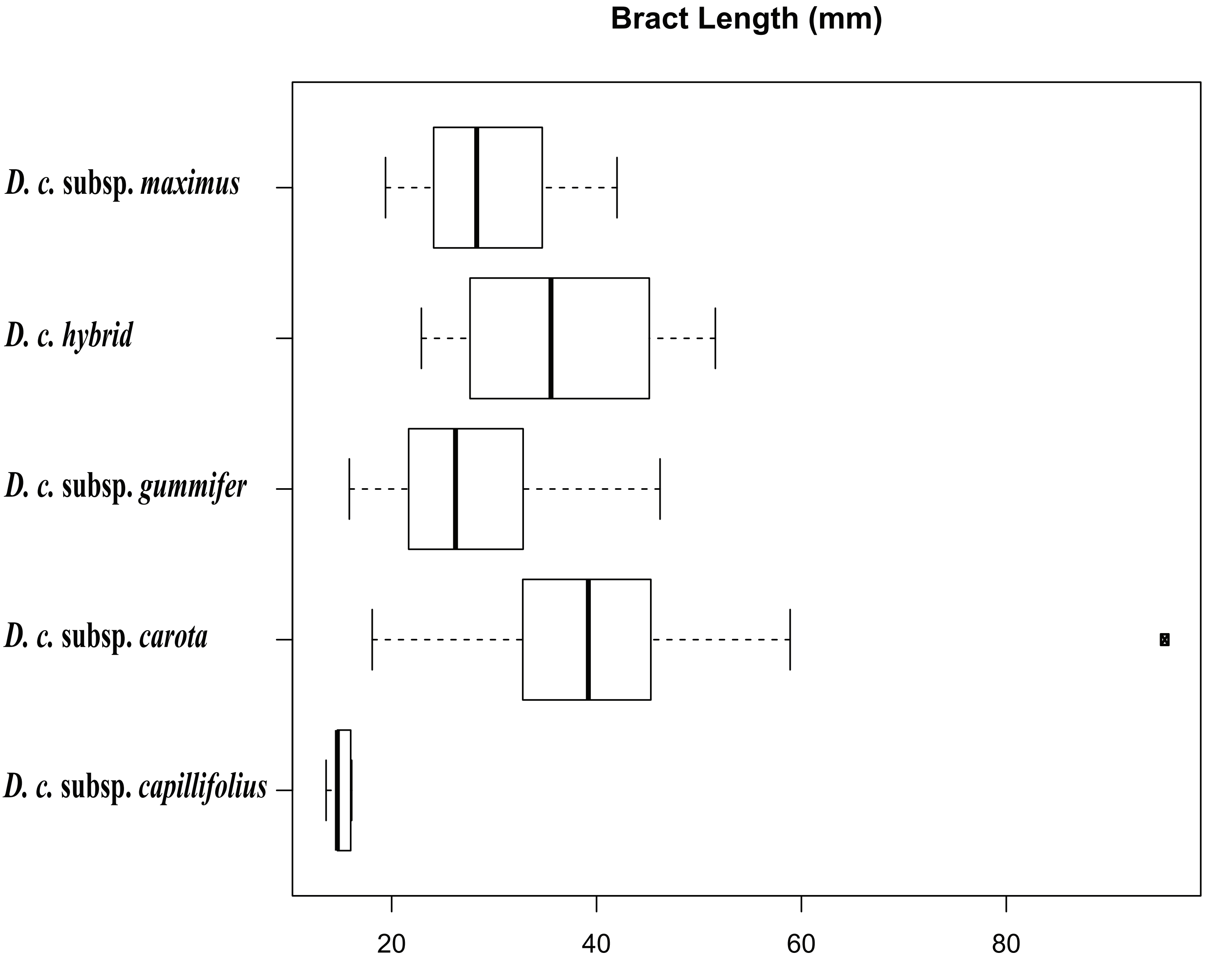

Bract Length (mm)
D. c. subsp. maximus
D. c. hybrid
D. c. subsp. gummifer
D. c. subsp. carota
D. c. subsp. capillifolius
20
40
60
80

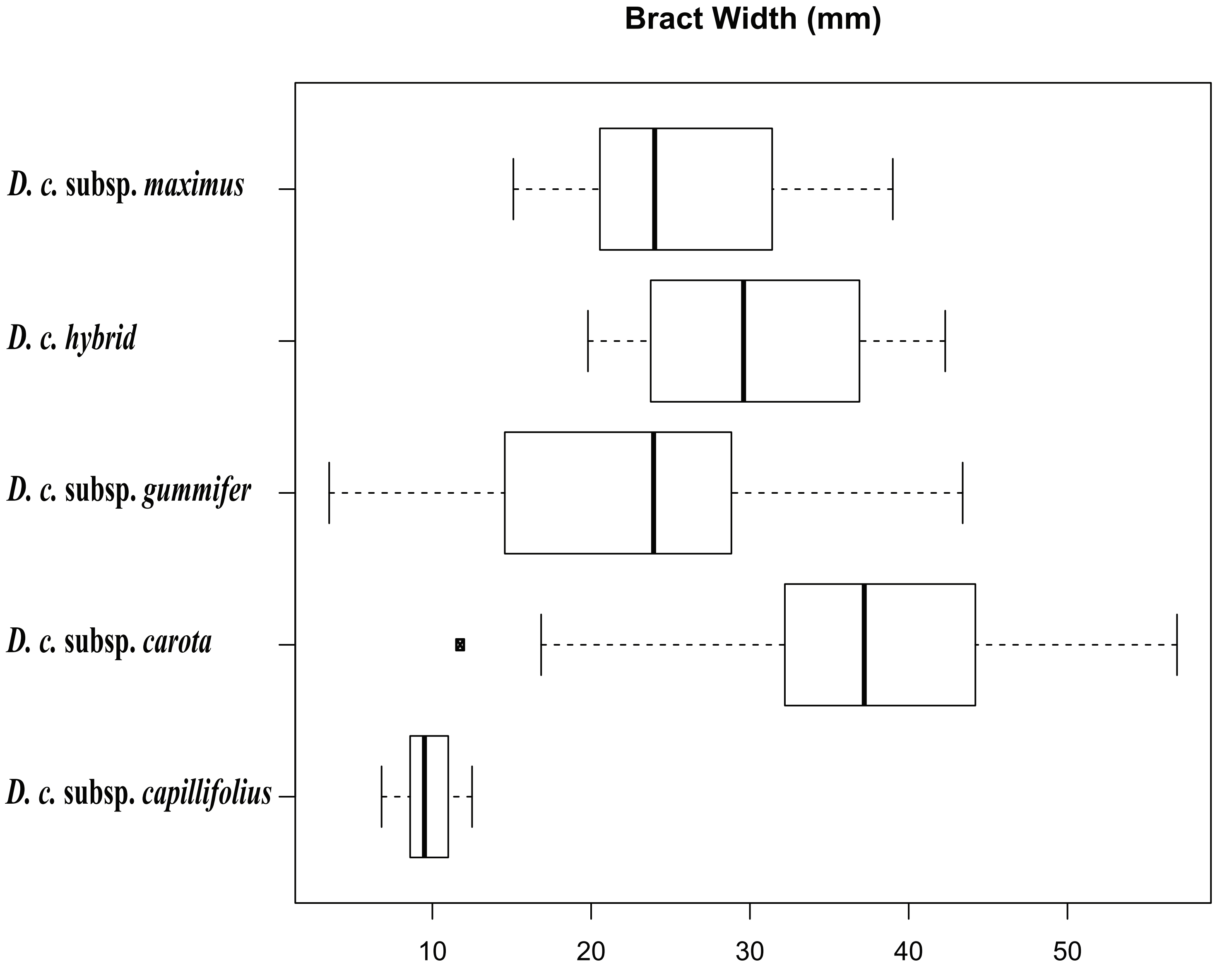

Bract Width (mm)
D. c. subsp. maximus
D. c. hybrid
D. c. subsp. gummifer
D. c. subsp. carota
D. c. subsp. capillifolius
10
20
30
40
50

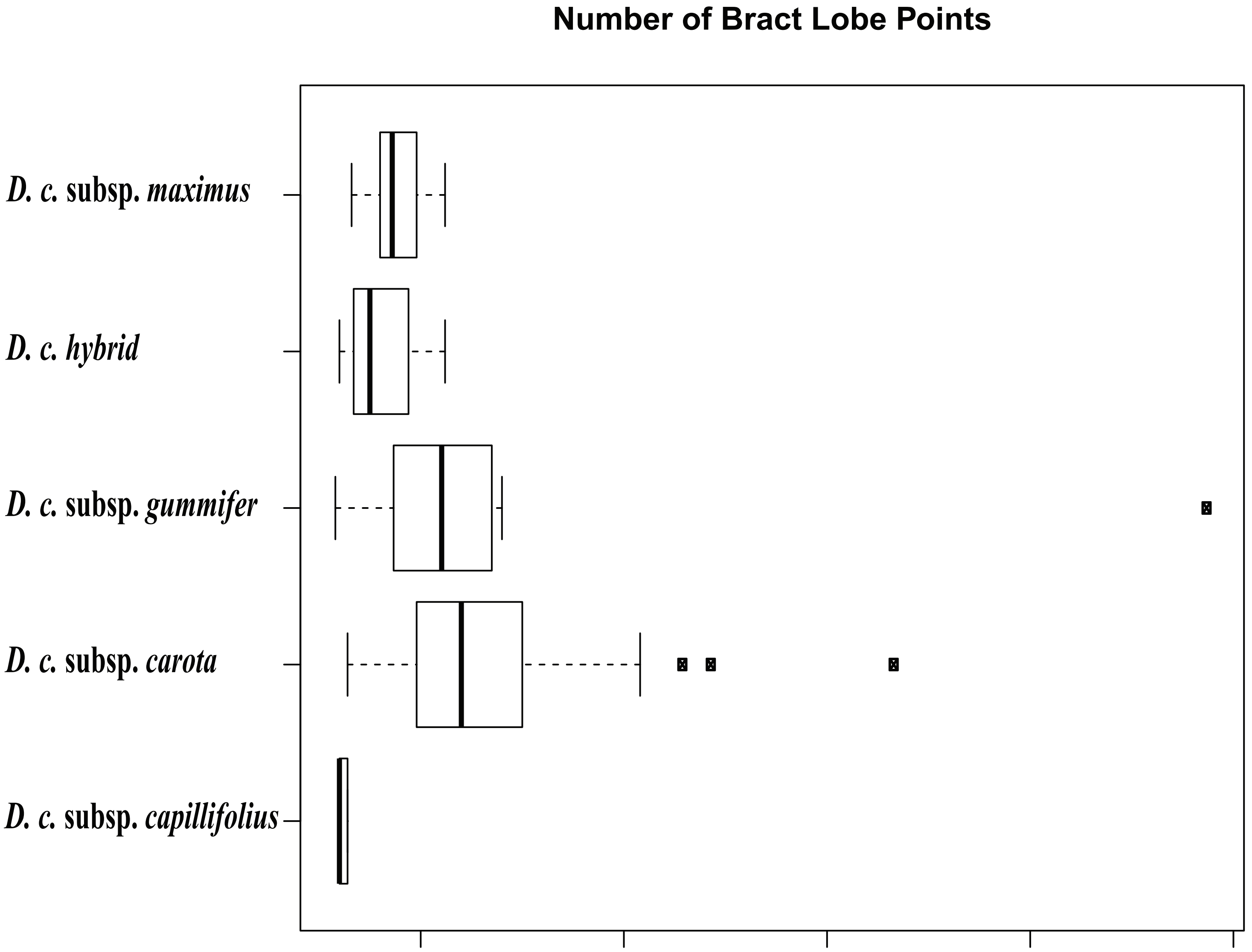
Number of Bract Lobe Points
D. c. subsp. maximus
D. c. hybrid
D. c. subsp. gummifer
D. c. subsp. carota
D. c. subsp. capillifolius
5
10
15
20
25

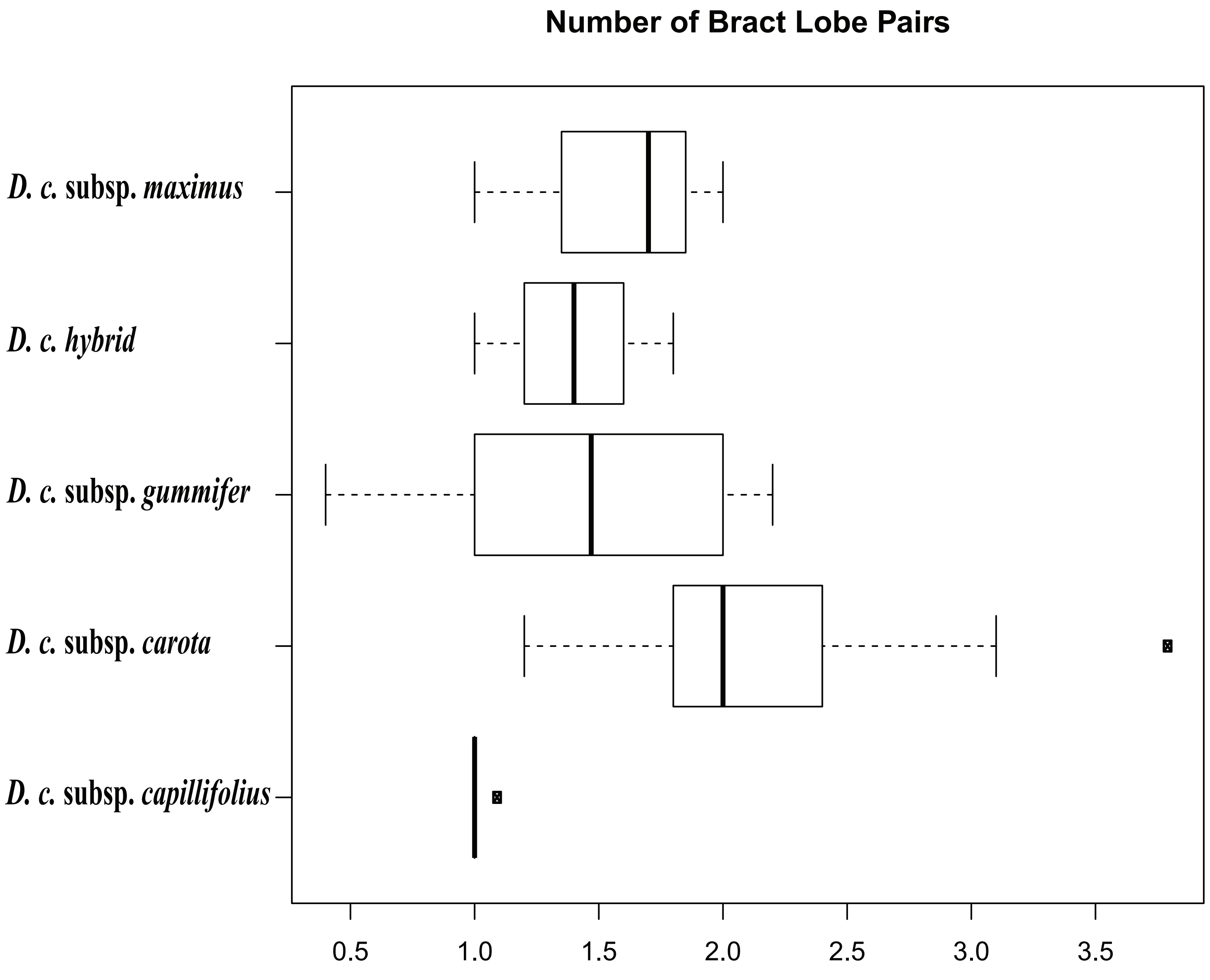

Number of Bract Lobe Pairs
D. c. subsp. maximus
D. c. hybrid
D. c. subsp. gummifer
D. c. subsp. carota
D. c. subsp. capillifolius
0.5
1.0
1.5
2.0
2.5
3.0
3.5

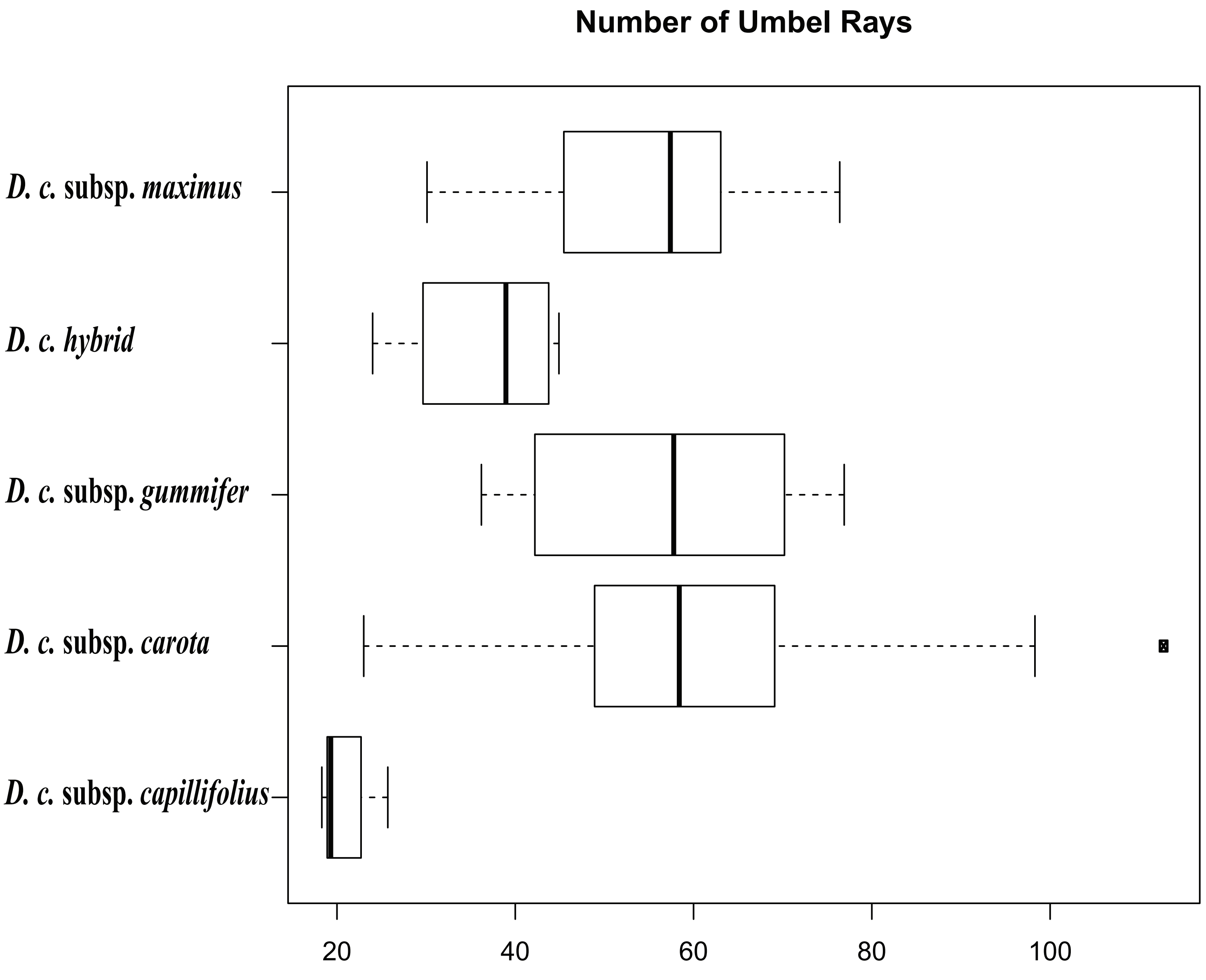

Number of Umbel Rays
D. c. subsp. maximus
D. c. hybrid
D. c. subsp. gummifer
D. c. subsp. carota
D. c. subsp. capillifolius
20
40
60
80
100

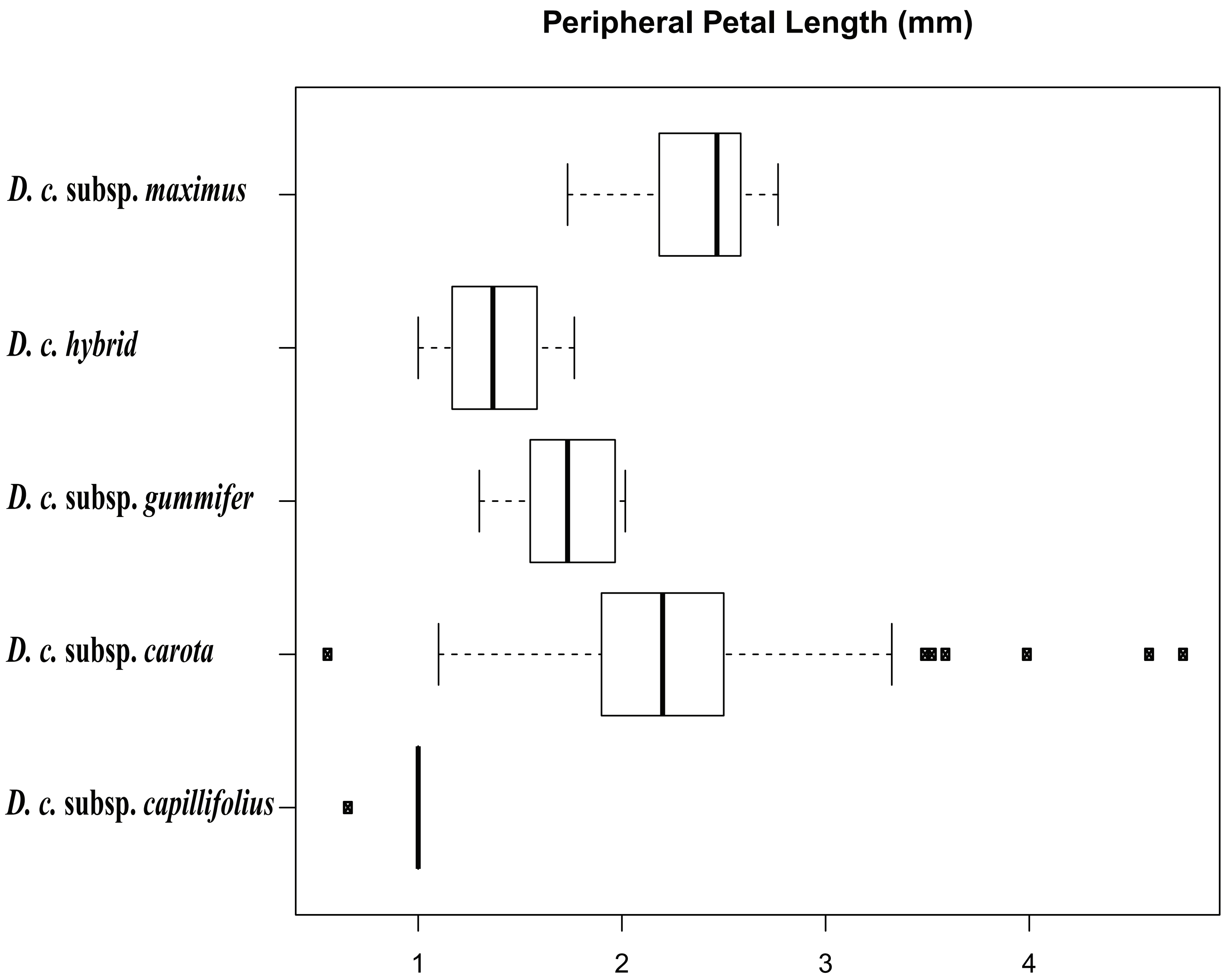

Peripheral Petal Length (mm)
D. c. subsp. maximus
D. c. hybrid
D. c. subsp. gummifer
D. c. subsp. carota
D. c. subsp. capillifolius
1
2
3
4

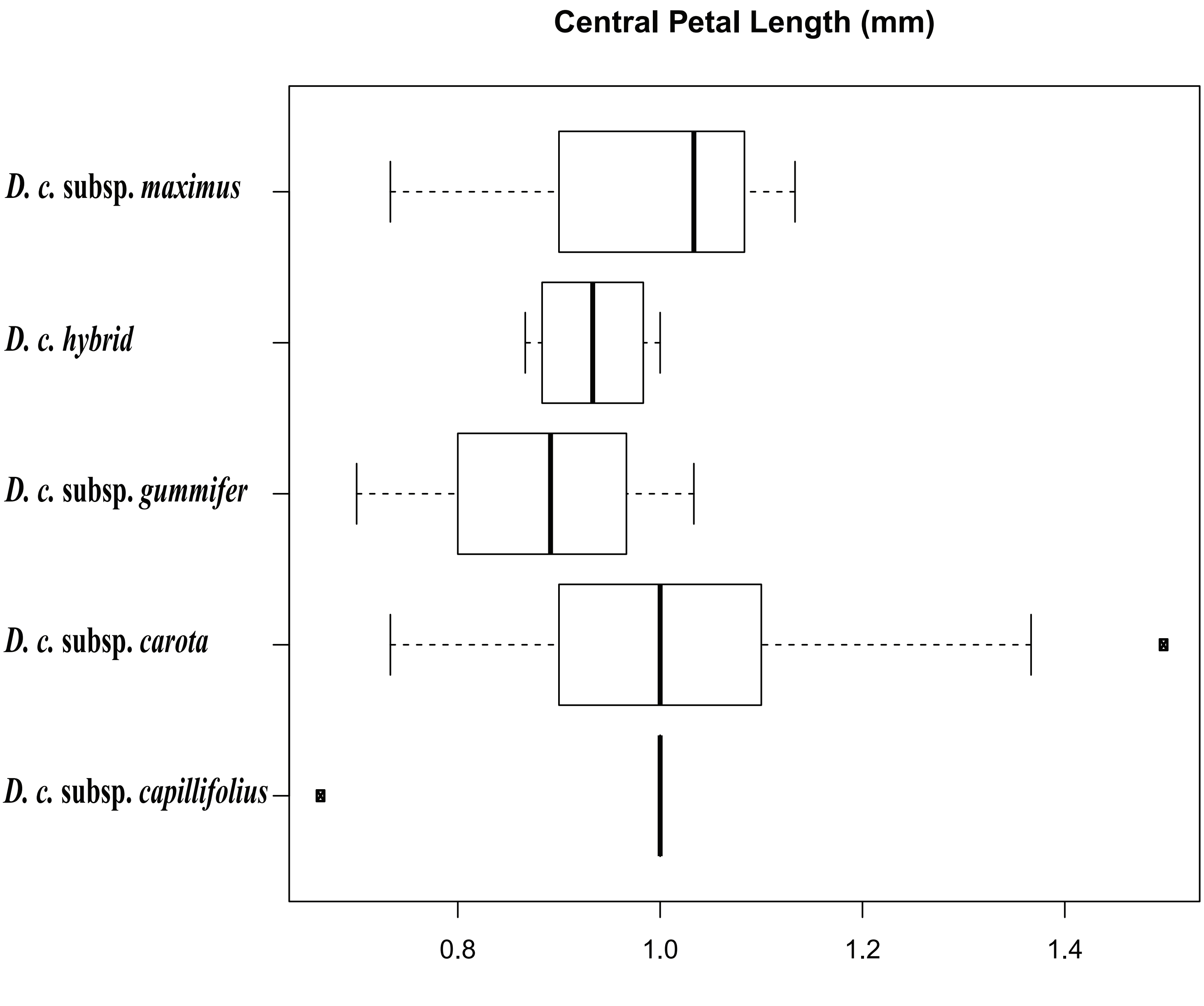

Central Petal Length (mm)
D. c. subsp. maximus
D. c. hybrid
D. c. subsp. gummifer
D. c. subsp. carota
D. c. subsp. capillifolius
0.8
1.0
1.2
1.4

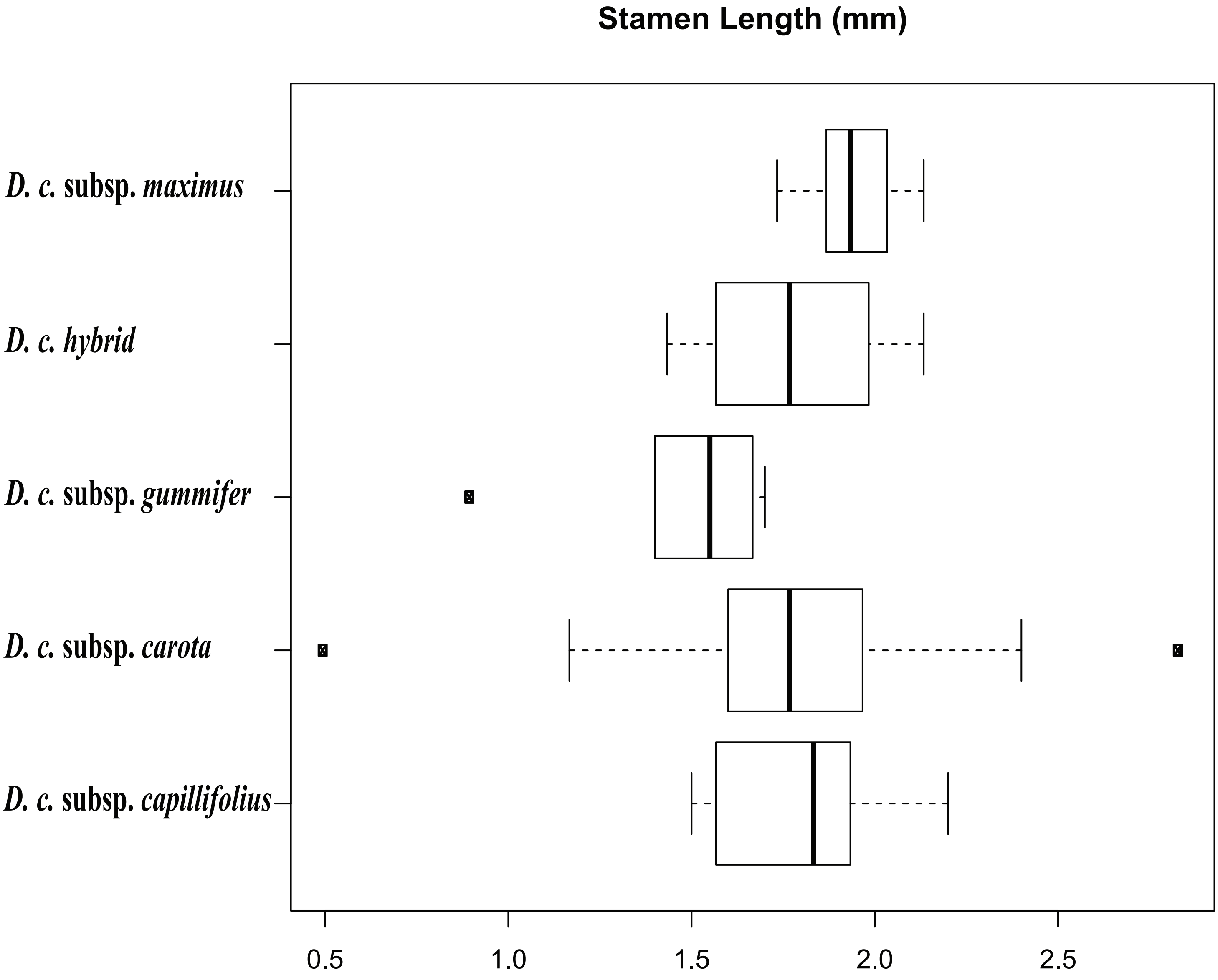
Stamen Length (mm)
D. c. subsp. maximus
D. c. hybrid
D. c. subsp. gummifer
D. c. subsp. carota
D. c. subsp. capillifolius
0.5
1.0
1.5
2.0
2.5

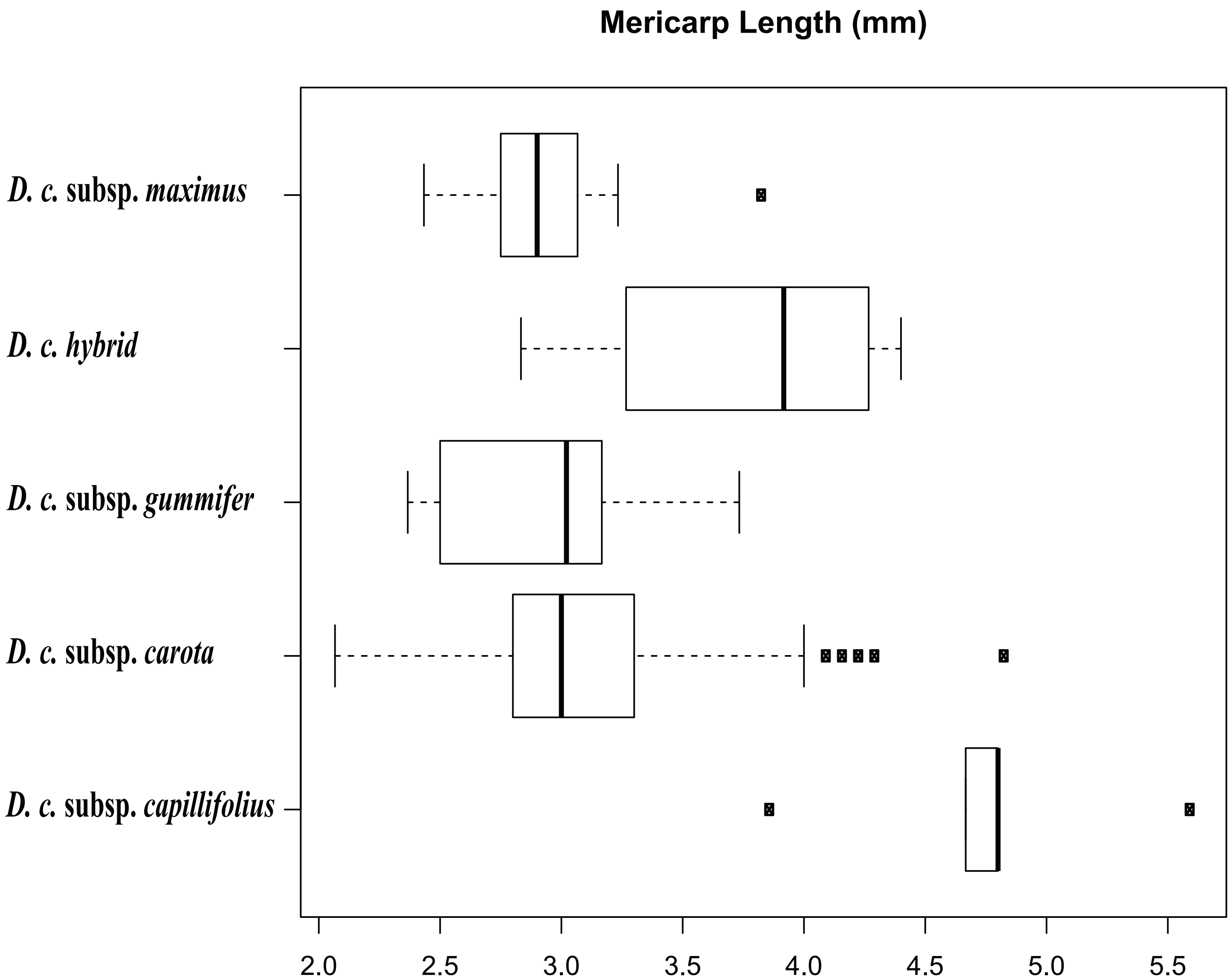
Mericarp Length (mm)
D. c. subsp. maximus
D. c. hybrid
D. c. subsp. gummifer
D. c. subsp. carota
D. c. subsp. capillifolius
2.0
2.5
3.0
3.5
4.0
4.5
5.0
5.5

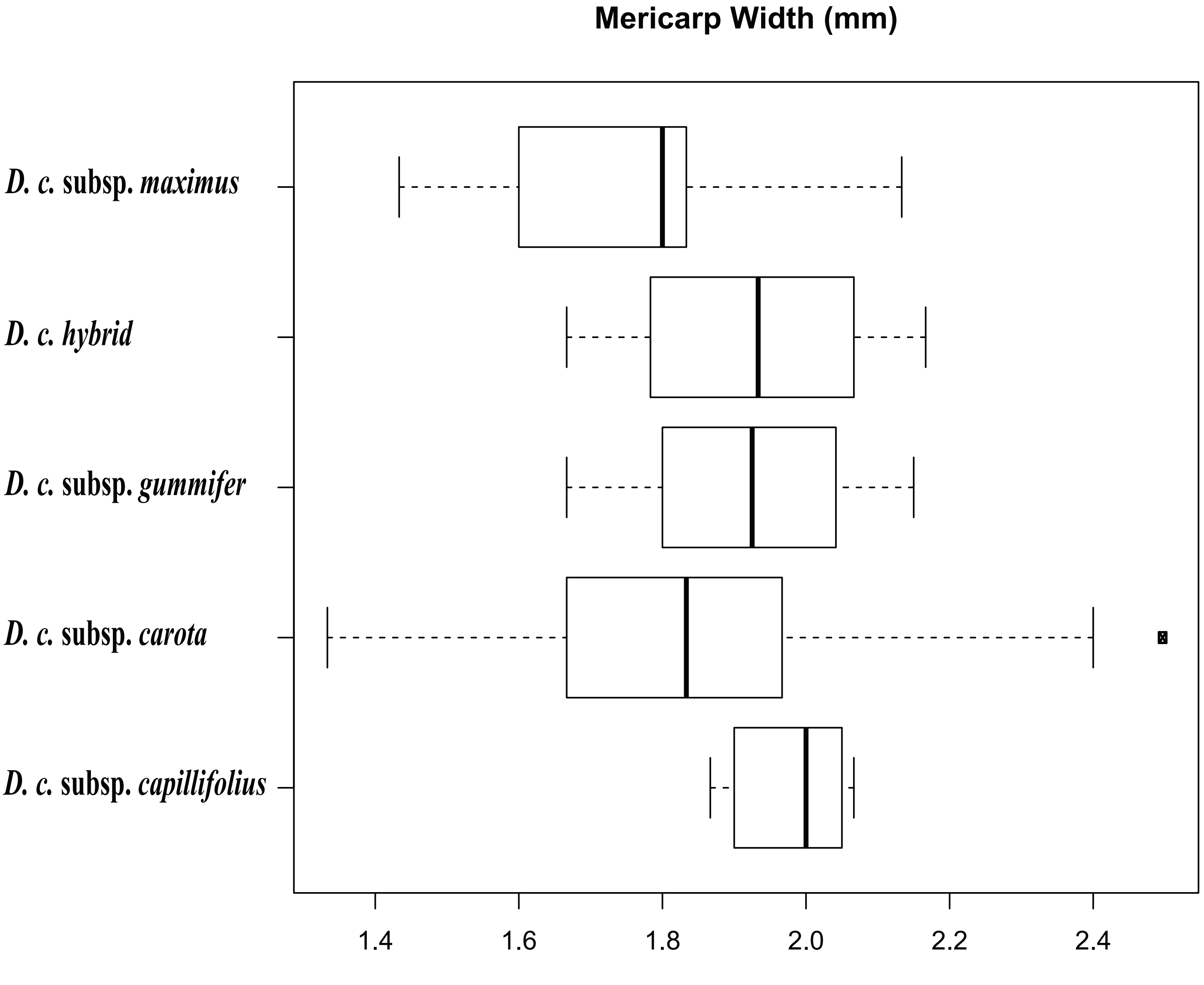
Mericarp Width (mm)
D. c. subsp. maximus
D. c. hybrid
D. c. subsp. gummifer
D. c. subsp. carota
D. c. subsp. capillifolius
1.4
1.6
1.8
2.0
2.2
2.4

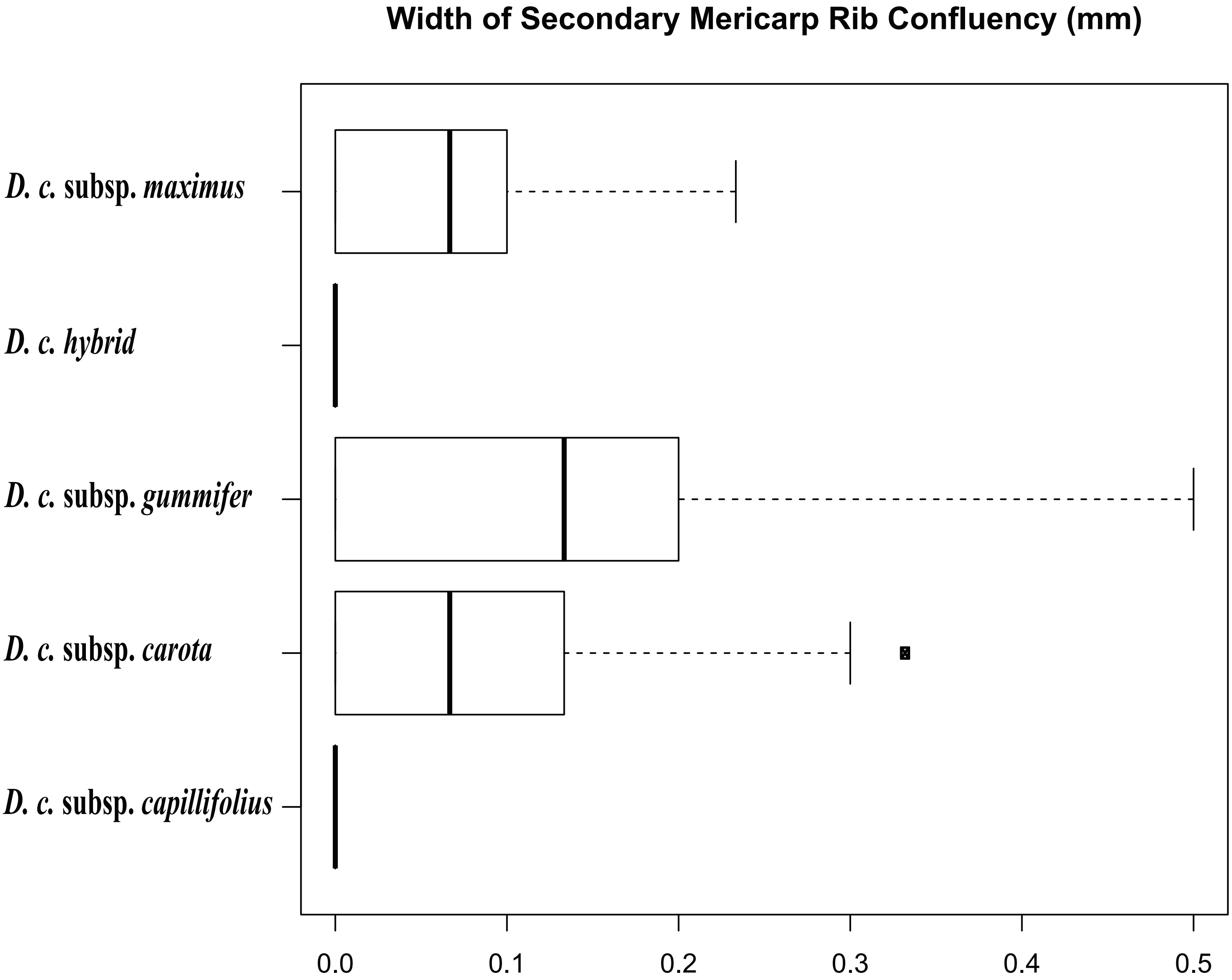

Width of Secondary Mericarp Rib Confluency (mm)
D. c. subsp. maximus
D. c. hybrid
D. c. subsp. gummifer
D. c. subsp. carota
D. c. subsp. capillifolius
0.0
0.1
0.2
0.3
0.4
0.5

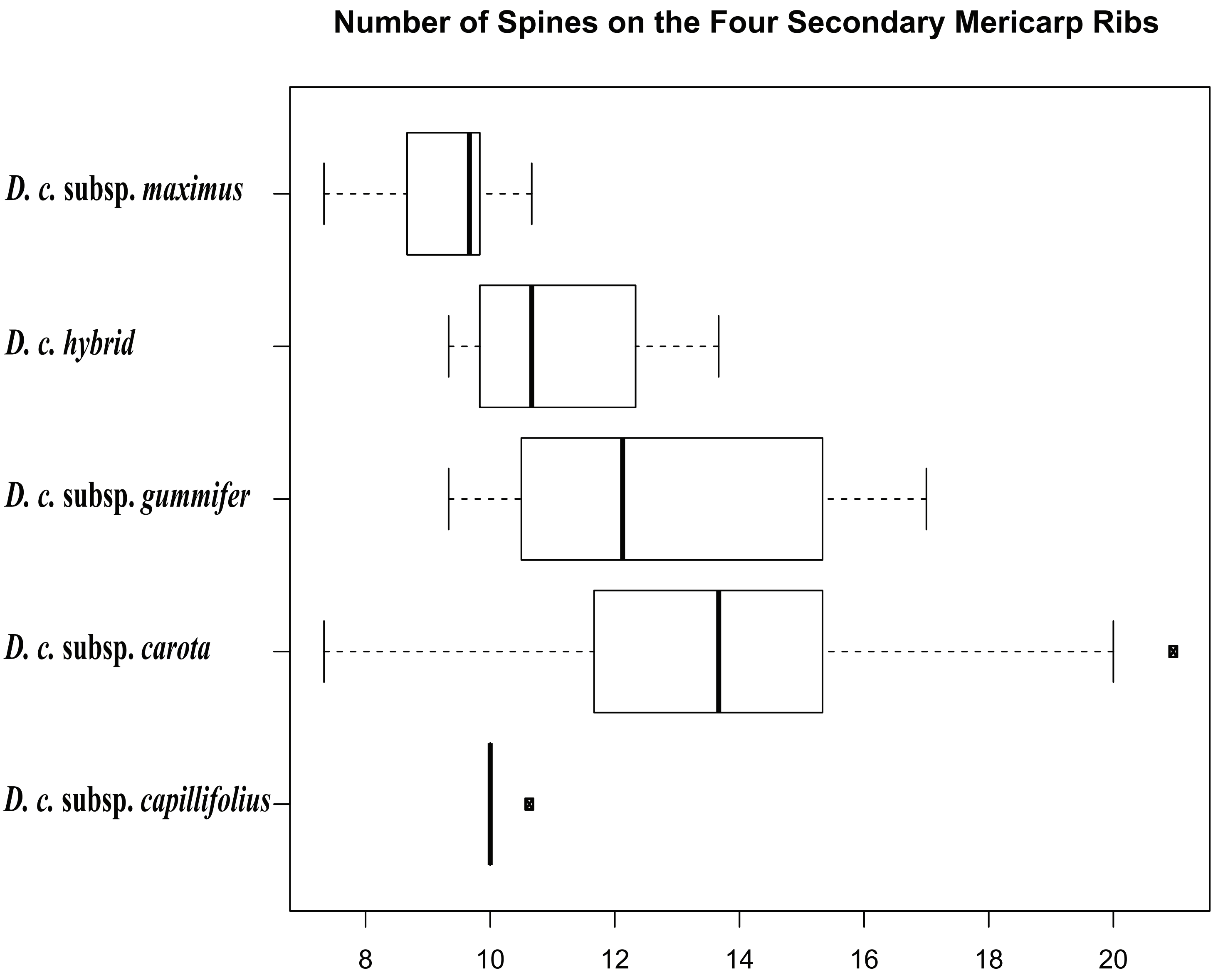

Number of Spines on the Four Secondary Mericarp Ribs
D. c. subsp. maximus
D. c. hybrid
D. c. subsp. gummifer
D. c. subsp. carota
D. c. subsp. capillifolius
8
10
12
14
16
18
20

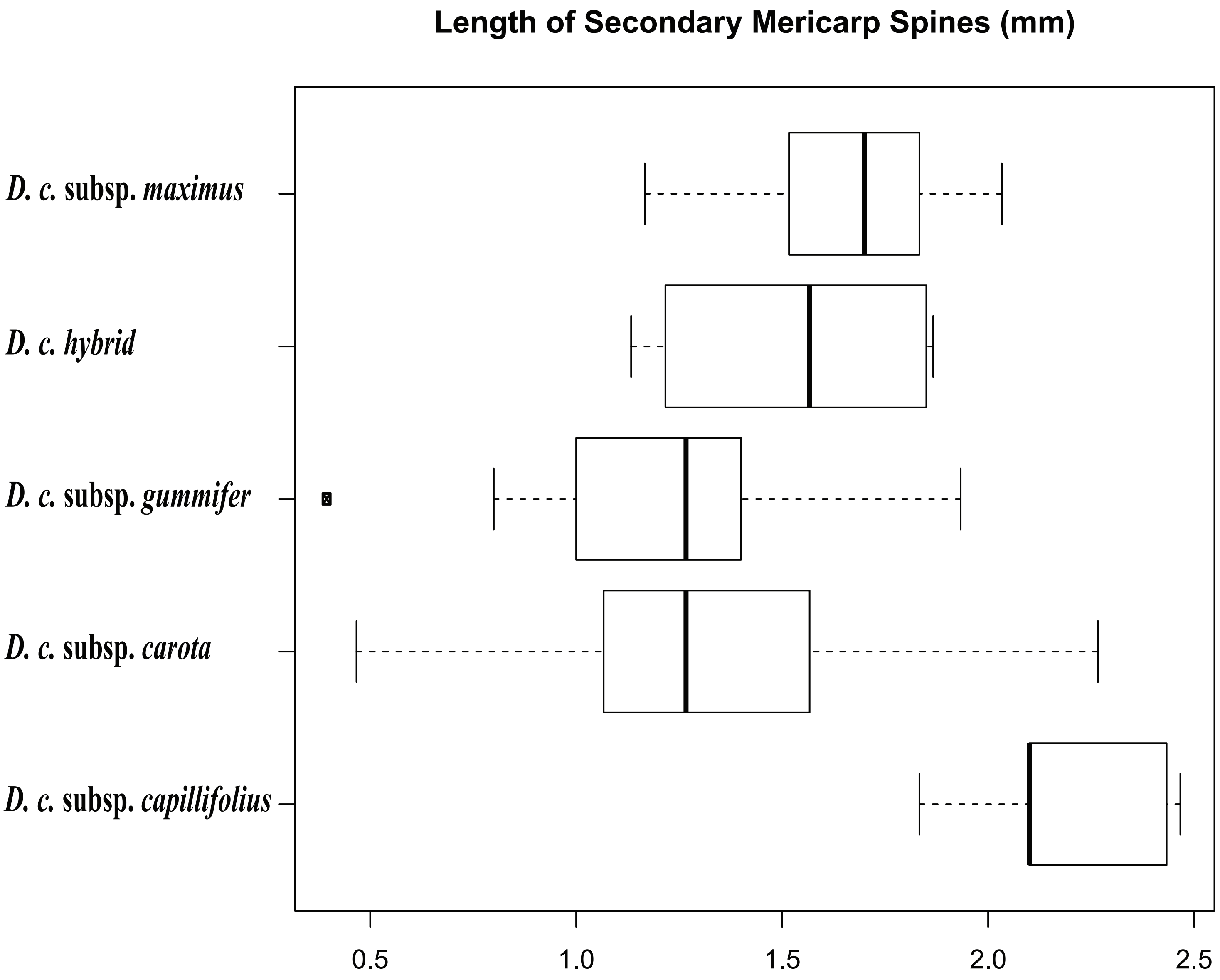

Length of Secondary Mericarp Spines (mm)
D. c. subsp. maximus
D. c. hybrid
D. c. subsp. gummifer
D. c. subsp. carota
D. c. subsp. capillifolius
0.5
1.0
1.5
2.0
2.5
